# Supplementary figures and images for: Pharmacological induction of autophagy reduces inflammation in macrophages by degrading immunoproteasome subunits
Source: PLoS Biol. 2024 Mar 6;22(3):e3002537. doi: 10.1371/journal.pbio.3002537 (PMC10917451; doi:10.1371/journal.pbio.3002537)

# S1 Figure

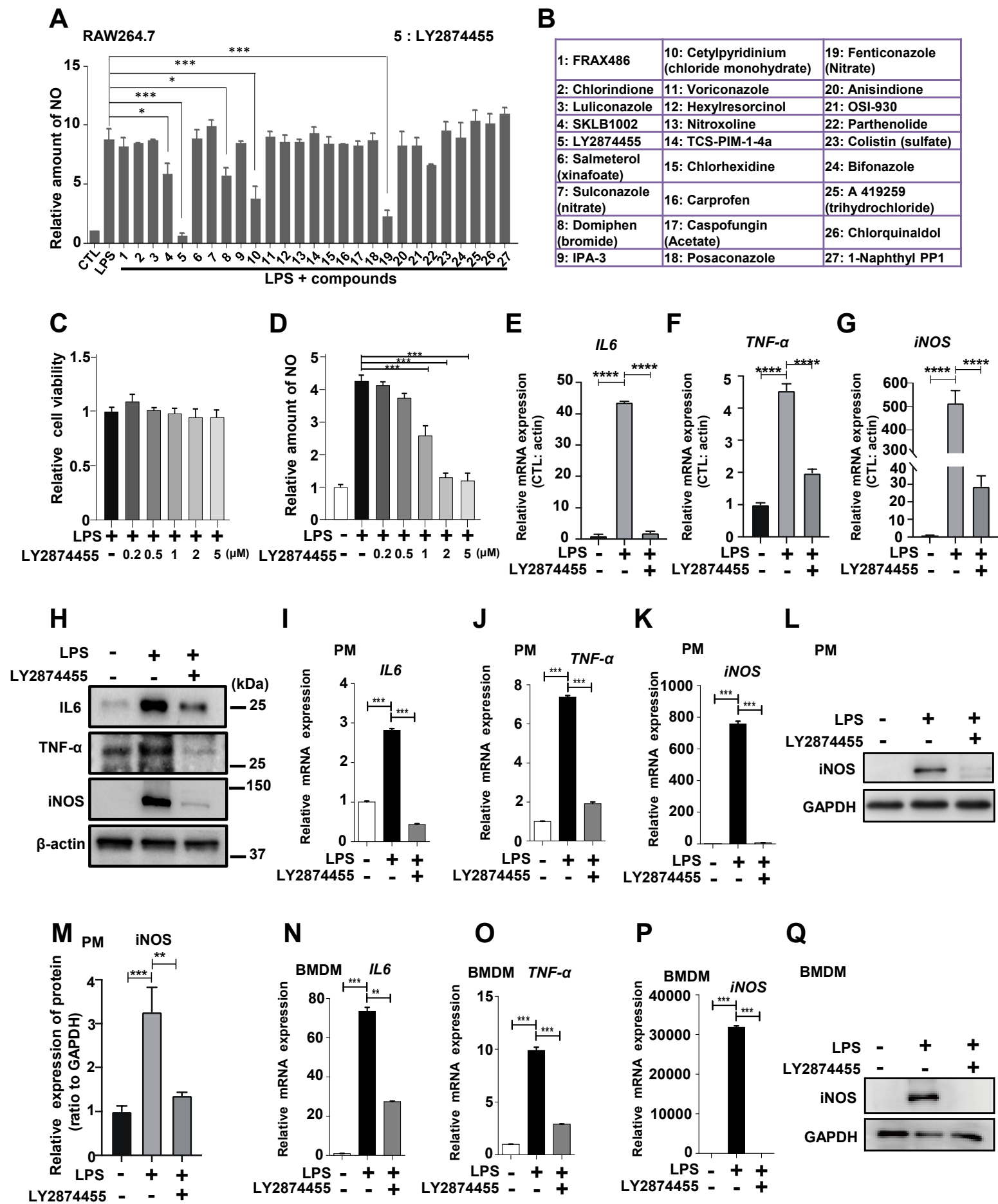

# S1 Figure

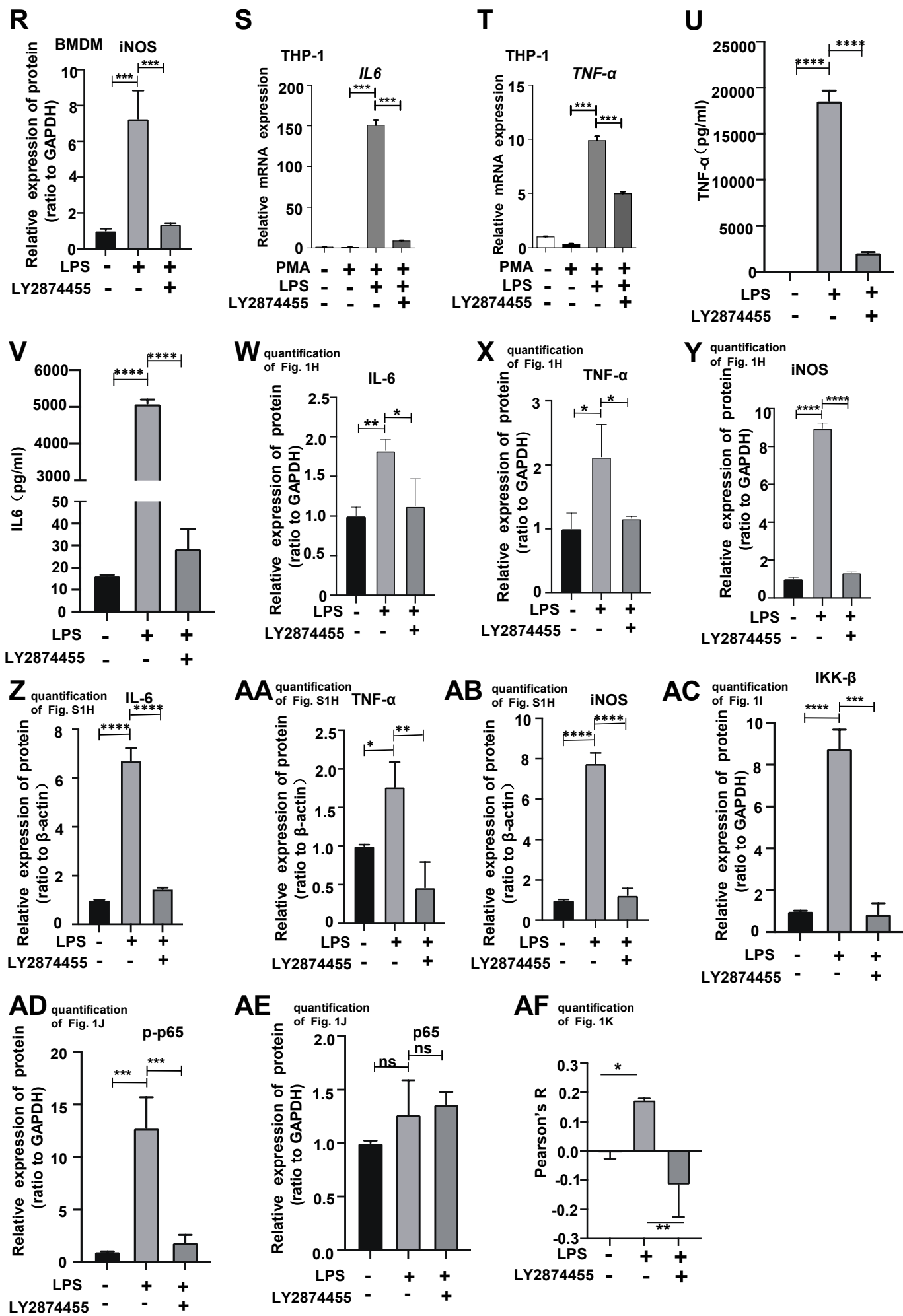

Supplement: S1 Fig — (A) RAW264.7 cells were incubated with 27 chemicals and stimulated with LPS (20 ng/ml) for 24 h. NO concentration in culture supernatant was measured and shown as fold change. (B) The names of the 27 chemicals. (C, D) Different concentrations of LY2874455 were applied to LPS (20 ng/ml)-treated RAW264.7 cells and cell viabilities and levels of NO were measured. (E–G) The expression of proinflammatory genes (IL-6, TNF-α) and iNOS was checked by qRT-PCR treated with LY2874455 (2 μm) and LPS (20 ng/ml) for 24 h. (H) Protein levels of IL-6, TNF-α, iNOS, and β-actin were detected by western blot in RAW264.7 cells. The blot was quantified with densitometric values and statistical significance was analyzed. (I–K) The expression of proinflammatory genes (IL-6, TNF-α) and iNOS was checked by qRT-PCR in PMs treated with LY2874455 (2 μm) and LPS (20 ng/ml) for 24 h. (L) Protein levels of iNOS and GAPDH were detected by western blot in PM. (M) The blot was quantified with densitometric values and statistical significance was analyzed. (N–P) The expression of proinflammatory genes (IL-6, TNF-α) and iNOS was checked by qRT-PCR in BMDMs treated with LY2874455 (2 μm) and LPS (20 ng/ml) for 24 h. (Q, R) Expression levels of iNOS were detected by in BMDM. The blot was quantified with densitometric values and statistical significance was analyzed. (S, T) The expression of proinflammatory genes (IL-6 and TNF-α) was checked by qRT-PCR in human THP-1 monocytes treated with LY2874455 (2 μm) and LPS (20 ng/ml) for 24 h. Phorbol12-myristate13-acetate (PMA) was used to induce THP-1 monocyte to macrophages. (U, V) The cytokine of IL-6 and TNF-α was detected by elisa in in RAW264.7 cells treated with LY2874455 (2 μm) and LPS (20 ng/ml) for 24 h. (W–Y) The protein expression of IL-6, TNF-α, and iNOS in Fig 1H were quantified with densitometric values and statistical significance was analyzed. (Z–AB) The protein expression of IL-6, TNF-α, and iNOS in S1H Fig were quantified with densitometric val [file pbio.3002537.s001.pdf]

# S2 Figure

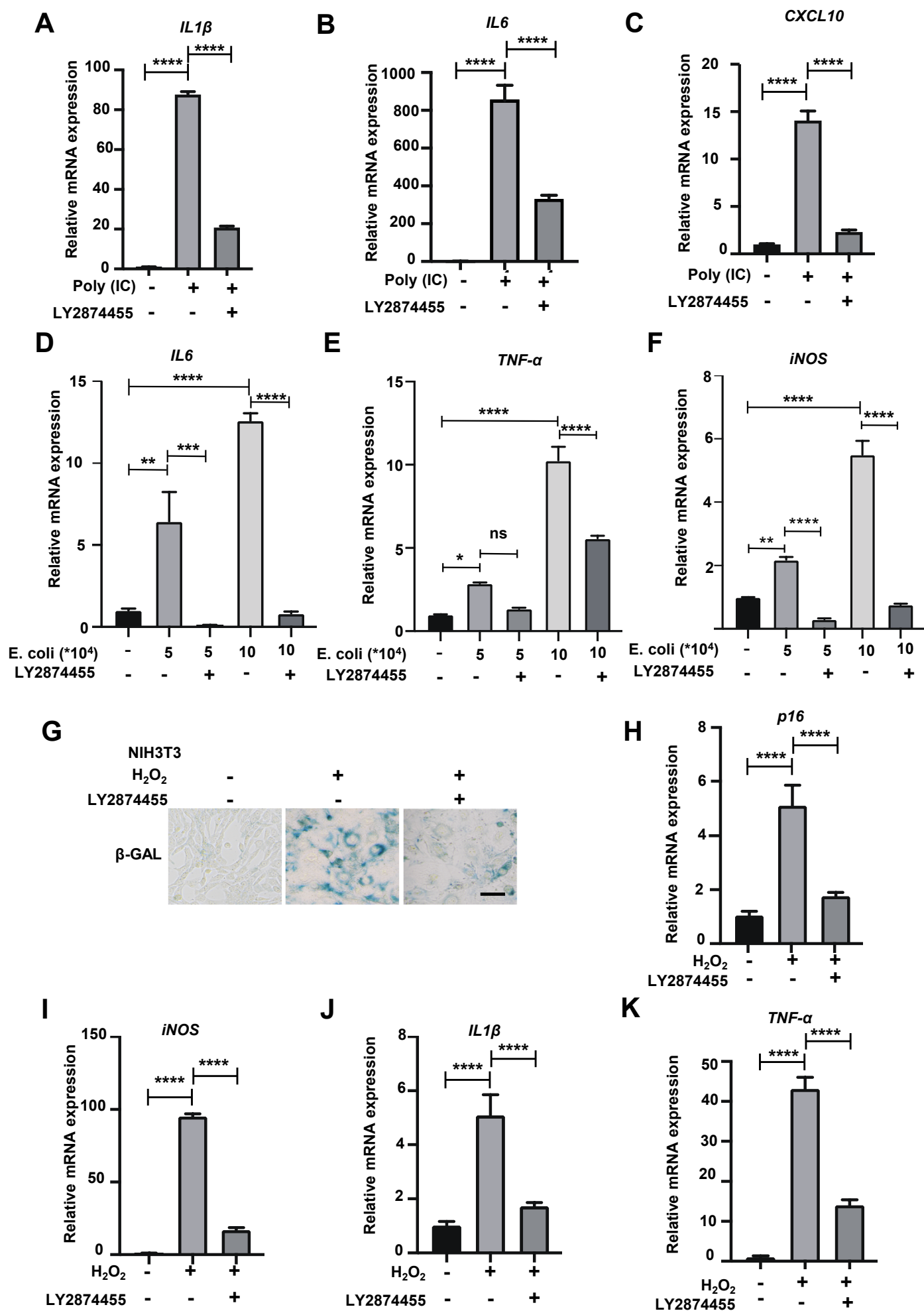

# S2 Figure

L

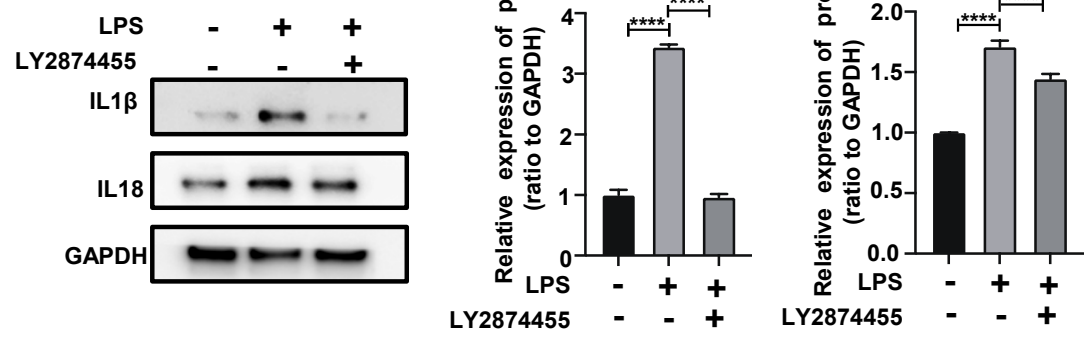

Supplement: S2 Fig — (A–C) LY2874455 inhibited viral infection-induced inflammation. Polyinosinic-polycytidylic acid (poly(I:C)) (50 μg/ml) and LY2874455 (2 μm) were applied to BMDM for 24 h and expression levels of inflammatory cytokines were analyzed qRT-PCR. (D–F) LY2874455 inhibited the bacteria (E. coli) infection-induced inflammation. E. coli and LY2874455 (2 μm) were applied to BMDM for 24 h and expression levels of inflammatory cytokines were analyzed qRT-PCR. (G–K) LY2874455 inhibited H2O2-induced cell aging. NIH3T3 cells were treated with H2O2 (400 μm for 2 h) for induction of cell aging followed by LY2874455 (2 μm) treatment. Representative images of β-GAL staining of the cells were shown (G) and the expression levels of p16 (H) and inflammatory cytokines (I–K) were analyzed by qRT-PCR. (L) The protein expression of IL1β, IL18, and GAPDH were detected by western blot. And the protein expression was quantified with densitometric values and statistical significance was analyzed. *: P < 0.05, **: P < 0.01, ***: P < 0.001, ****: P < 0.0001, NS: no statistical difference. The data underlying the graphs shown in the figure can be found in S1 Data. (PDF) [file pbio.3002537.s002.pdf]

# S3 Figure

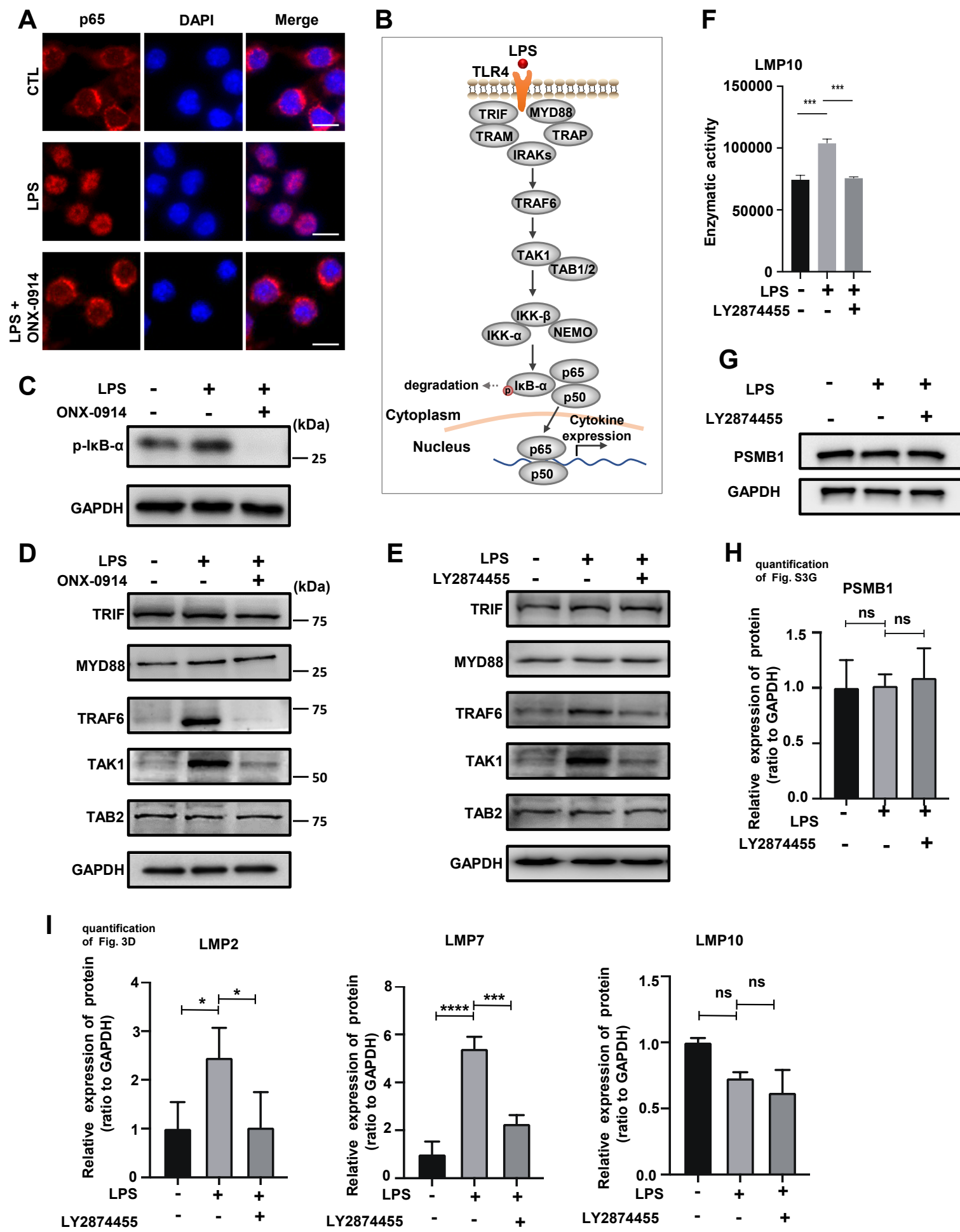

# S3 Figure

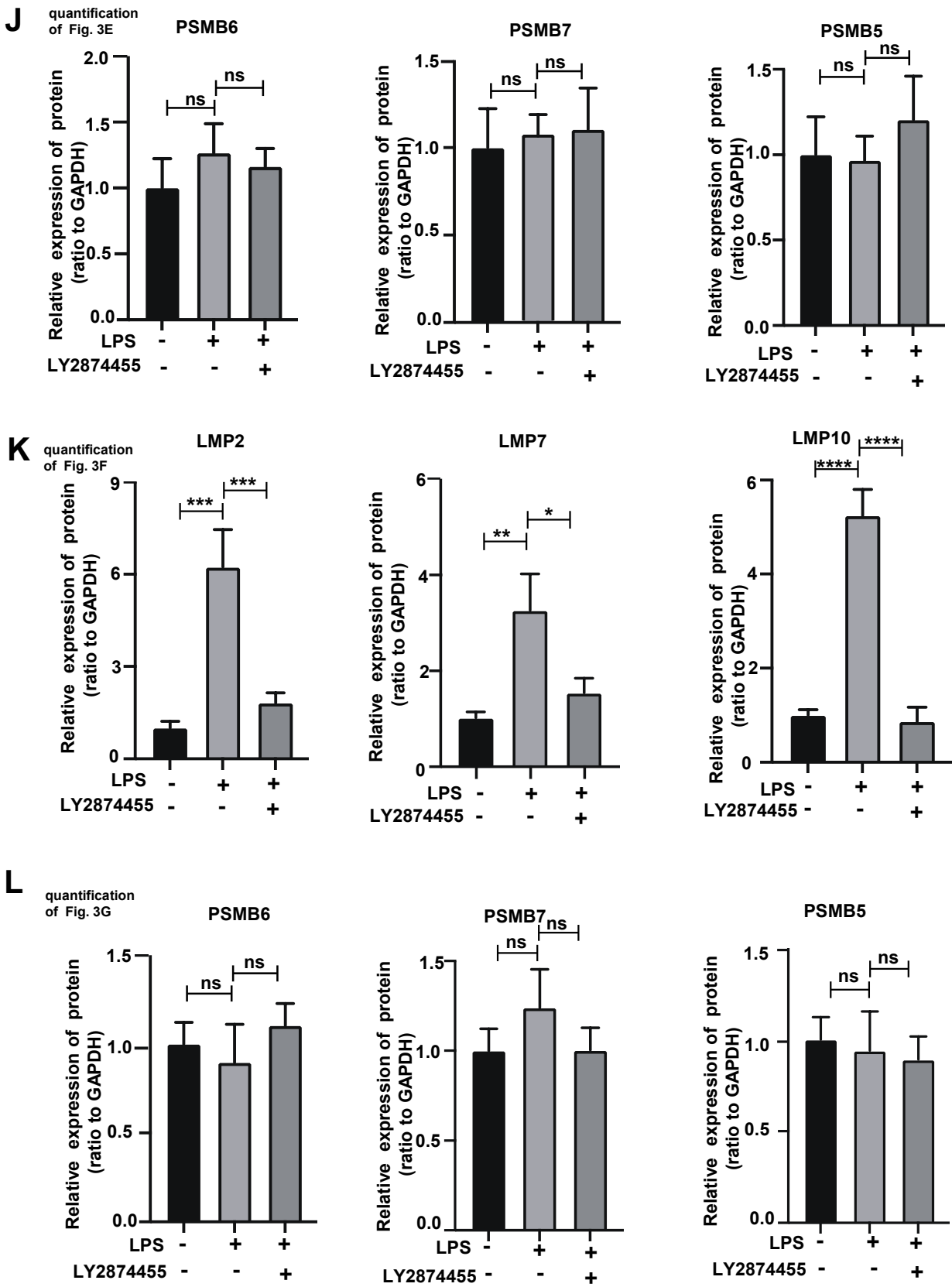

# S3 Figure

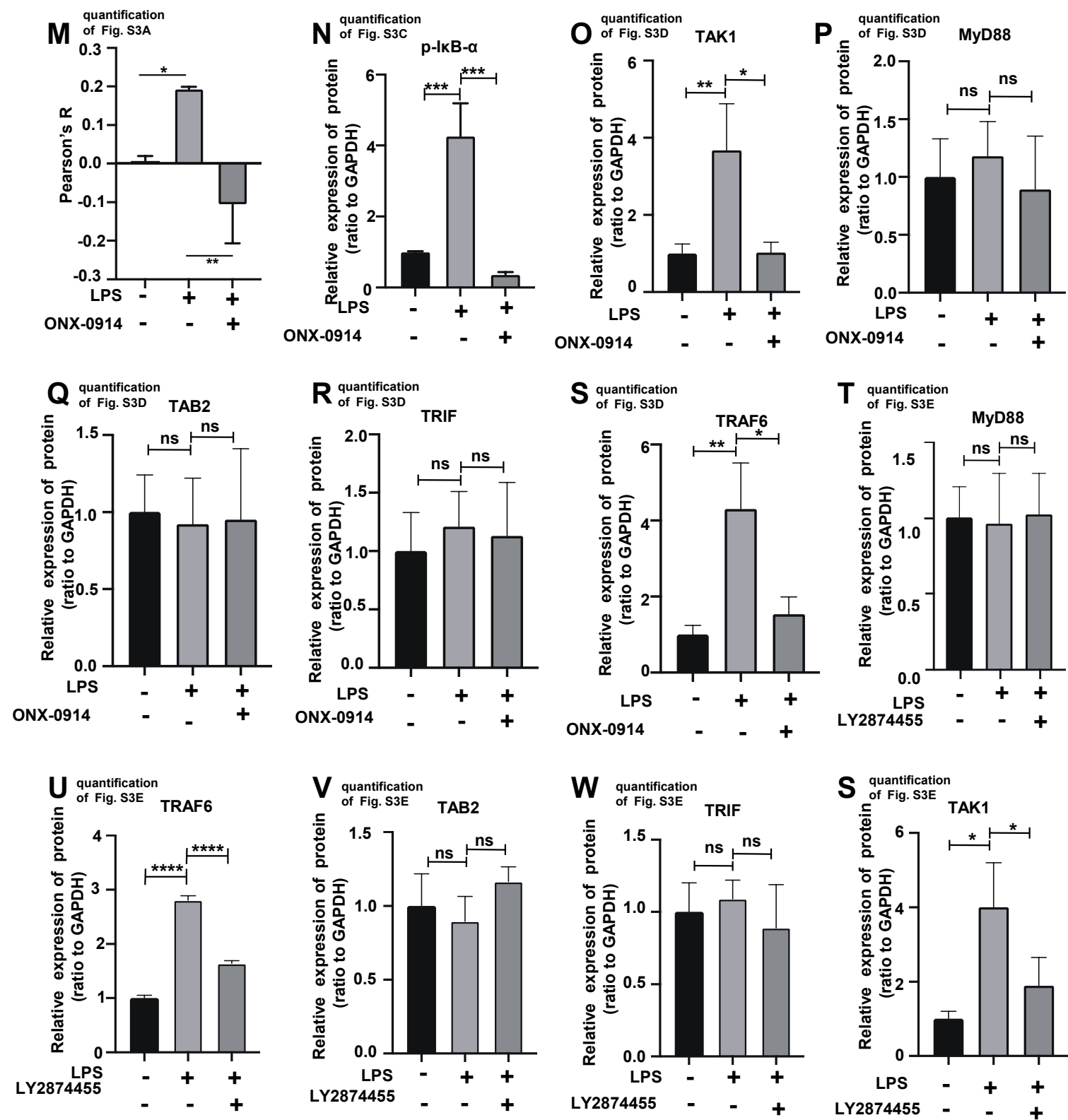

Supplement: S3 Fig — (A) Immunoproteasome inhibitor ONX-0914 suppressed the nuclear translocation of NF-κB factor p65 in macrophages stimulated with LPS. RAW264.7 cells were stimulated with LPS (20 ng/ml) for 2 h with or without ONX-0914 (1 μm). Representative images of 3 independent replicates were shown. Scale bars: 10 μm. (B) Schematic diagram of NF-κB inflammation signaling pathway. (C) Total proteins were extracted from RAW264.7 macrophage cells treated with LPS (20 ng/ml) together with ONX-0914 (1 μm) and used for western blot analysis of protein levels of phosphorylated IκB-α (p-IκB-α). The blot was quantified with densitometric values and statistical significance was analyzed. (D, E) Total proteins were extracted from RAW264.7 macrophage cells treated with LPS (20 ng/ml) together with ONX-0914 (1 μm) or LY2874455 (2 μm) and used for western blot analysis of protein levels of indicated factors function upstream of the NF-κB signaling pathway. The blots were quantified with densitometric values and statistical significance was analyzed. (F) The enzymatic activities of immunoproteasome subunits LMP10 were measured in RAW264.7 cells. ***: P < 0.001. (G) Total proteins were extracted from RAW264.7 macrophage cells treated with LPS (20 ng/ml) together with LY2874455 (2 μm) and used for western blot analysis of protein levels of PSMB1 and GAPDH. (H) The protein expression of PSMB1 in was quantified with densitometric values and statistical significance was analyzed. (I) The protein expression of LMP2, LMP7, and LMP10 were quantified with densitometric values and statistical significance was analyzed. (J) The protein expression of PSMB6, PSMB7, and PSMB5 in Fig 3E were quantified with densitometric values and statistical significance was analyzed. (K) The protein expression of LMP2, LMP7, and LMP10 in Fig 3F was quantified with densitometric values and statistical significance was analyzed. (L) The protein expression of PSMB6, PSMB7, and PSMB5 in Fig 3G were quantified with densitometri [file pbio.3002537.s003.pdf]

# S5 Figure

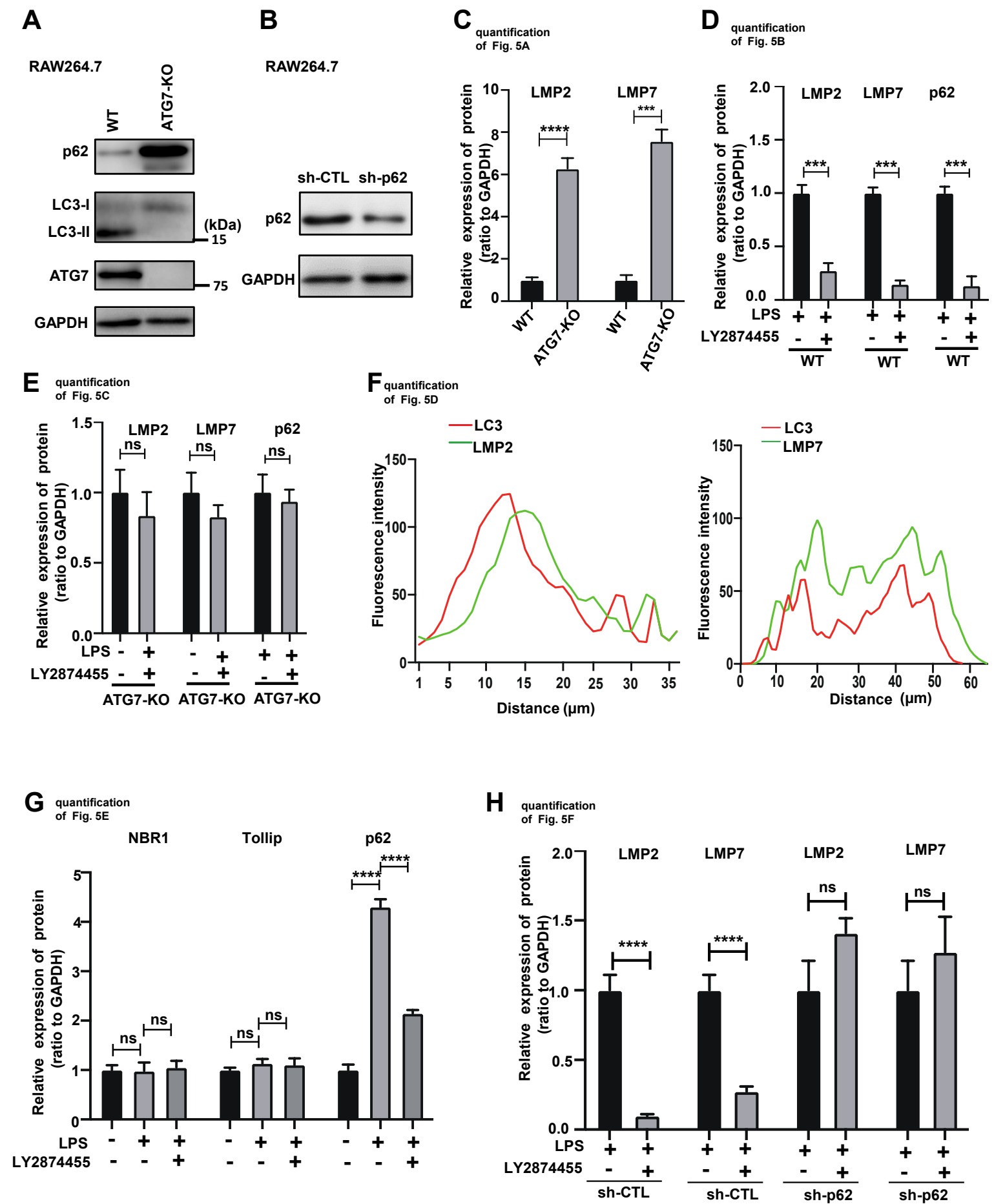

Supplement: S5 Fig — (A) Confirmation of ATG7 gene knock out in macrophages. ATG7 gene was deleted through CRISPR-CAS9 assay in RAW264.7 cells. Clones of gene deletion cells were cultured and detected for protein levels of p62, LC3, ATG7, and intrinsic control GAPDH. The blots were quantified with densitometric values and statistical significance was analyzed. (B) Confirmation of the decrease of p62 protein levels in p62 knock down RAW264.7 cells. The blot was quantified with densitometric values and statistical significance was analyzed. (C) The protein expression of LMP2 and LMP7 in Fig 5A were quantified with densitometric values and statistical significance was analyzed. (D) The protein expression of LMP2, LMP7, and p62 in Fig 5B were quantified with densitometric values and statistical significance was analyzed. (E) The protein expression of LMP2, LMP7, and p62 in Fig 5C were quantified with densitometric values and statistical significance was analyzed. (F) The immune fluorescence micrographs in Fig 5D was quantified by ImageJ. (G) The protein expression of NRB1, Tollip, and p62 in Fig 5E were quantified with densitometric values and statistical significance was analyzed. (H) The protein expression of LMP2 and LMP7 in Fig 5F were quantified with densitometric values and statistical significance was analyzed. *: P < 0.05, **: P < 0.01, ***: P < 0.001, ****: P < 0.0001, NS: no statistical difference. The data underlying the graphs shown in the figure can be found in S1 Data. (PDF) [file pbio.3002537.s005.pdf]

# S6 Figure

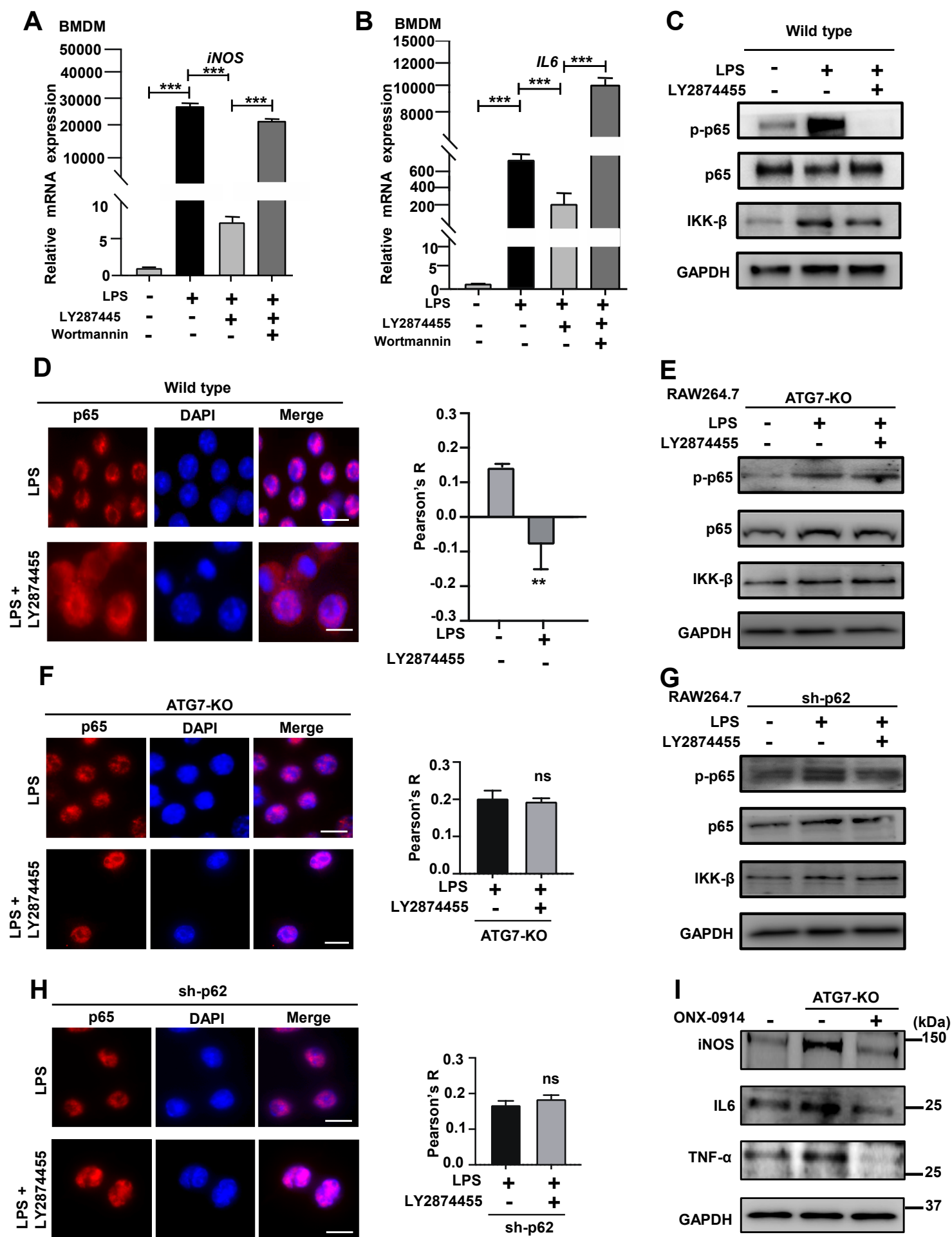

# S6 Figure

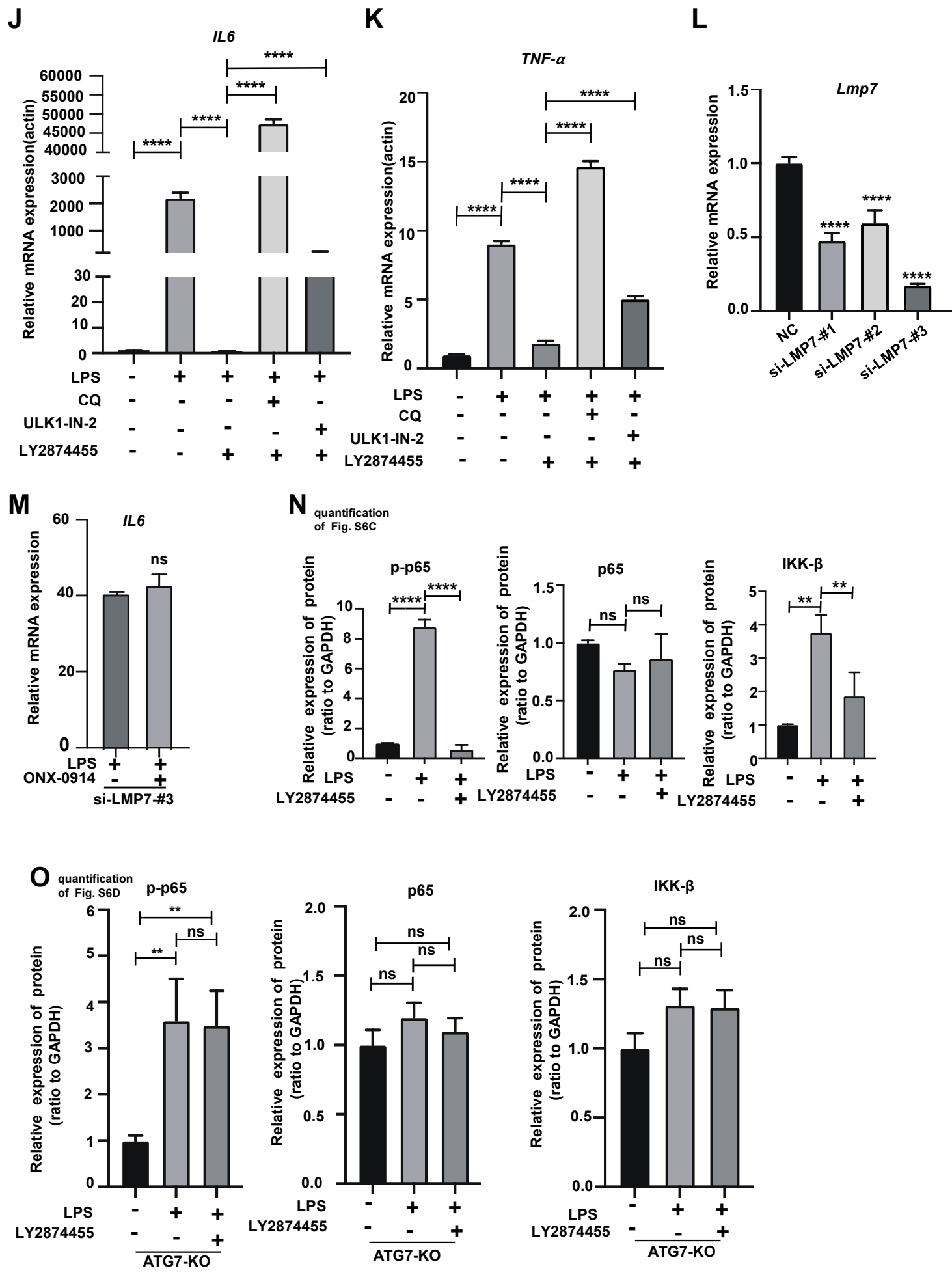

# S6 Figure

**P**

quantification  
of Fig. S6G

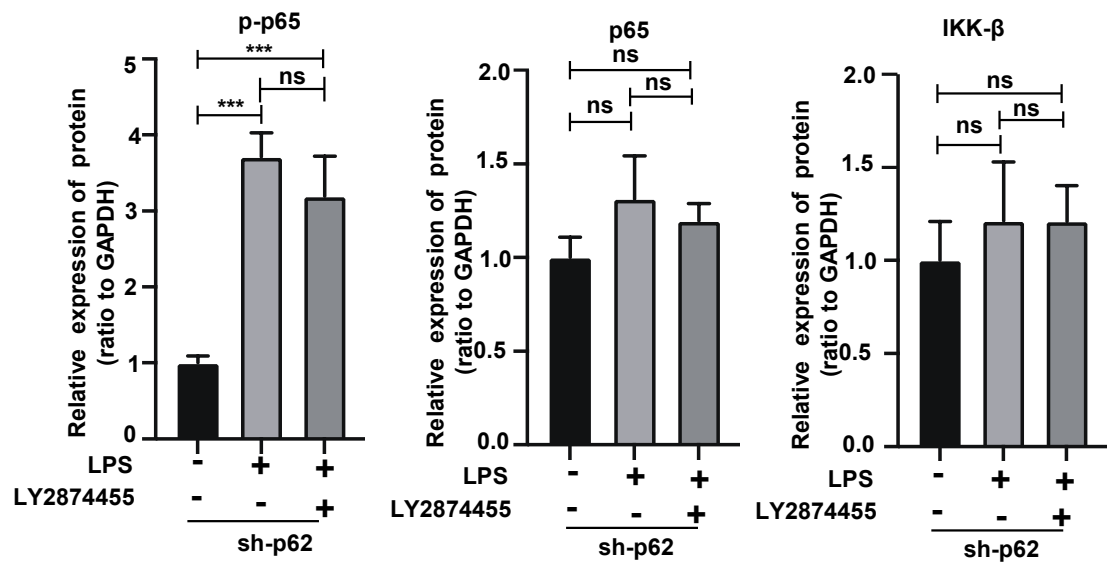

**Q**

quantification  
of Fig. S6I

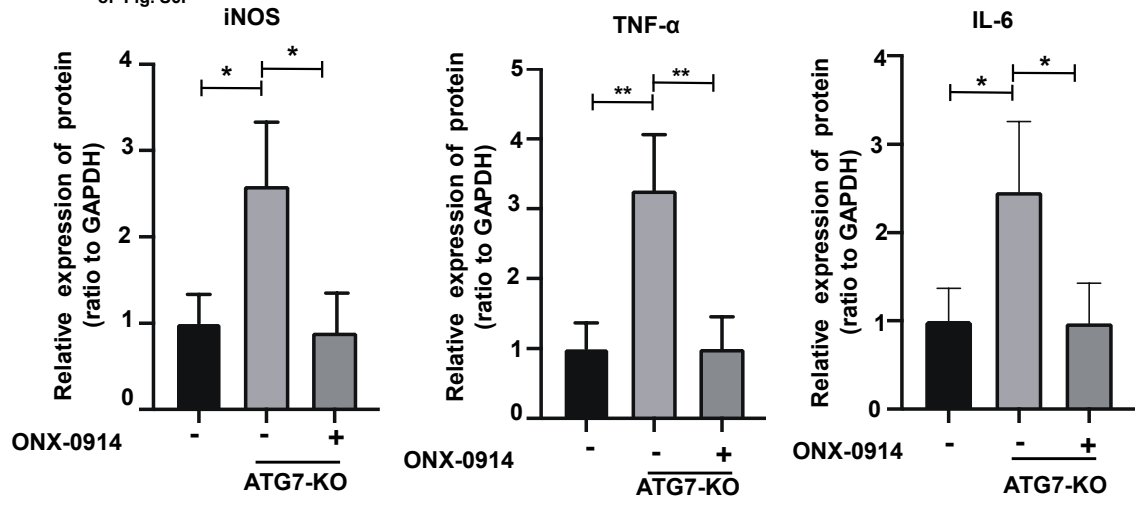

**R**

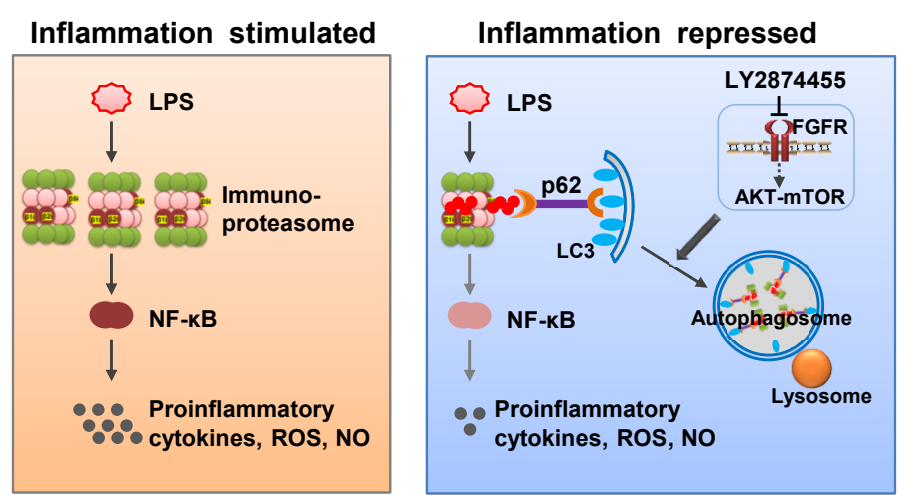

Supplement: S6 Fig — (A, B) Autophagy inhibitor wortmannin reversed the effect of LY2874455 in LPS-stimulated macrophages. BMDMs were treated with LPS (20 ng/ml), LY2874455 (2 μm), and wortmannin (100 nM) as indicated for 24 h. The expression of iNOS and inflammatory cytokine genes IL-6 was checked by qRT-PCR. (C) RAW264.7 cells and used for western blot analysis of protein levels of p65, phosphorylated p65 (p-p65), and IKK-β. The blots were quantified with densitometric values and statistical significance was analyzed. (D) RAW264.7 cells were stimulated with LPS (20 ng/ml) for 2 h with or without LY2874455 (2 μm). Representative images of double immunostaining for p65 and nucleus (DAPI) were shown. Scale bars: 10 μm. (D, F) Deficiency of autophagy factor ATG7 or receptor p62 caused blockage of LY2874455 function in suppression of NF-κB factor p65 (phosphorylation and nucleus translocation). (E, G) Total proteins were extracted from ATG7 knockout or p62 knockdown RAW264.7 cells and used for western blot analysis of protein levels of p65, phosphorylated p65 (p-p65), and IKK-β. The blots were quantified with densitometric values and statistical significance was analyzed. (F, H) ATG7 knockout or p62 knockdown RAW264.7 cells were stimulated with LPS (20 ng/ml) for 2 h with or without LY2874455 (2 μm). Representative images of double immunostaining for p65 and nucleus (DAPI) were shown. Scale bars: 10 μm. (I) The enhanced inflammatory capacity in autophagy-deficient macrophages was suppressed by immunoproteasome inhibitor ONX-0914. Wild-type (WT) or ATG7 knockout (ATG7-KO) RAW264.7 cells treated with LPS (20 ng/ml) and ONX-0914 (1 μm). The protein levels of iNOS and inflammatory cytokine genes (IL-6 and TNF-α) were checked. The blots were quantified with densitometric values and statistical significance was analyzed. (J, K) Autophagy inhibitors (ULK1-IN-2 and chloroquine (CQ)) reversed the effect of LY2874455 in LPS-stimulated macrophages. RAW264.7 cells were harvest after treatment with LPS [file pbio.3002537.s006.pdf]
