# Supplementary material for: Pharmacological induction of autophagy reduces inflammation in macrophages by degrading immunoproteasome subunits
Source: PLoS Biol. 2024 Mar 6;22(3):e3002537. doi: 10.1371/journal.pbio.3002537 (PMC10917451; doi:10.1371/journal.pbio.3002537)
Supplement: S4 Fig — (A) Inhibitors MG132, chloroquine, or wortmannin were used at different concentrations to analyze their effects on macrophage viabilities in the dose- and time-dependent cell death assays. (B) Inhibitor of constitutive proteasome, MG132, could not block the reduction of immunoproteasome subunits induced by LY2874455. RAW264.7 cells were treated with LPS (20 ng/ml), LY2874455 (2 μm), and proteasome inhibitor MG132 (5 μm). Protein levels of LMP2, LMP7, and GAPDH were detected by western blot. The blots were quantified with densitometric values and statistical significance was analyzed. (C) Autophagy inhibitor wortmannin blocked the reduction of immunoproteasome subunits induced by LY2874455. Protein levels of LMP2, LMP7, and GAPDH were analyzed in RAW264.7 cells treated with LPS (20 ng/ml), LY2874455 (2 μm), and autophagy inhibitor wortmannin (100 nM). The blots were quantified with densitometric values and statistical significance was analyzed. (D) Model scheme of LY2874455 effect on activation of autophagy. FGFRs are transmembrane RTKs and activated by FGF to form dimers, leading to the activation of downstream FRS2 complex and subsequent activation of PI3K/AKT/mTOR signaling pathway. Activated mTOR then phosphorylates and inhibits autophagy initiating ULK1 kinase complex, leading to suppression of autophagy. LY2874455 suppresses the FGFR activities and the downstream PI3K/AKT/mTOR signaling pathway, leading to the release and activation of autophagy. (E) NIH3T3 cells were treated with LPS (20 ng/ml) and LY2874455 (0.2, 0.5, 1, 2, 5 μm) for 24 h and protein levels of p62, LC3, and GAPDH were analyzed by western blot. The blots were quantified with densitometric values and statistical significance was analyzed. (F) AZD4547, an inhibitor of FGFR, was used to detect the effect on autophagy and immunoproteasome. RAW264.7 cells were treated with LPS (20 ng/ml) and ADZ4547 (10, 50, 100 nM) for 24 h and protein levels of p62, LC3, and GAPDH were analyzed by western blot. T [file pbio.3002537.s004.pdf]

S4 Figure

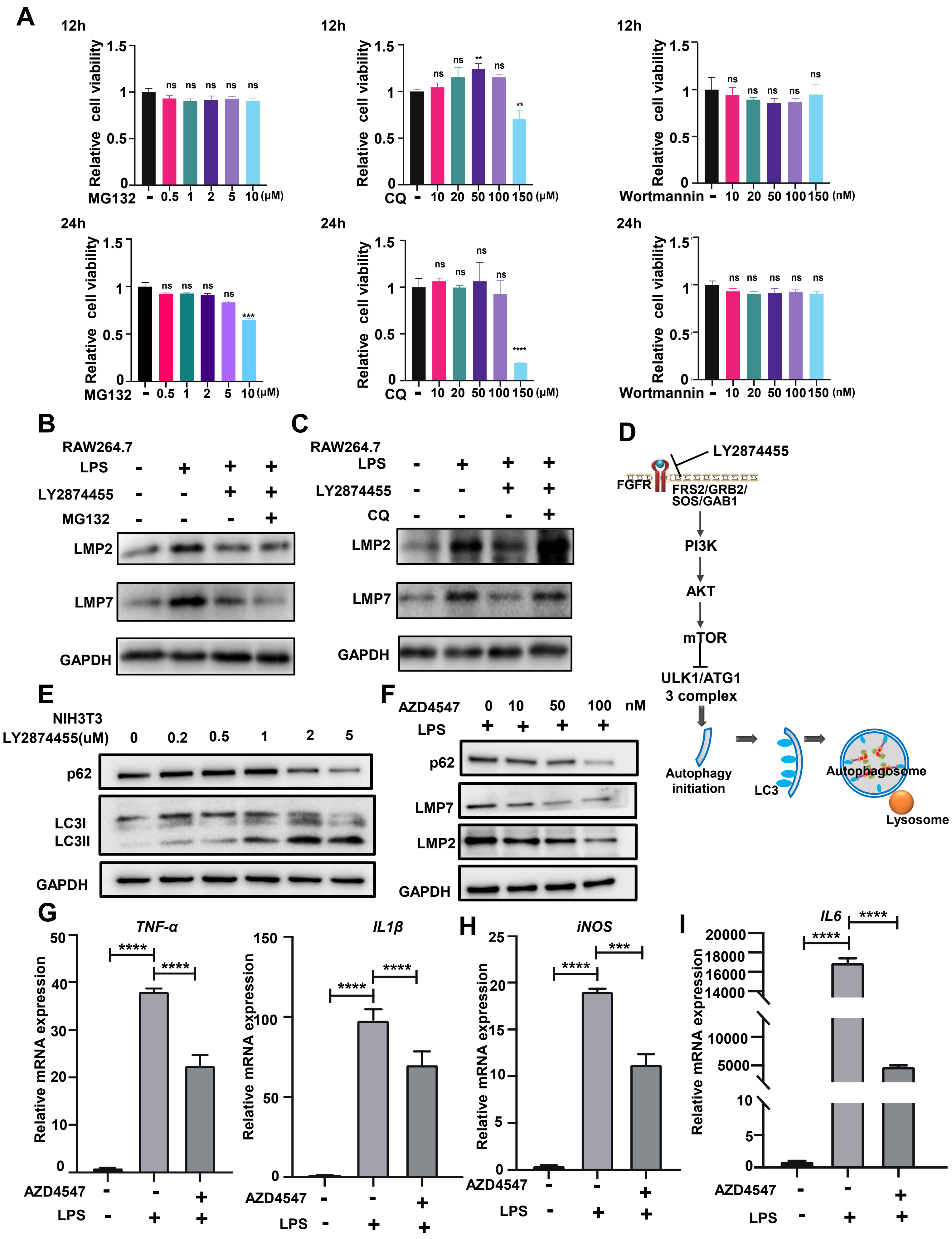
A
12h
Relative cell viability
MG132 - 0.5 1 2 5 10 (μM)
CQ - 10 20 50 100 150 (μM)
Wortmannin - 10 20 50 100 150 (nM)
24h
B
RAW264.7
LPS
LY2874455
MG132
LMP2
LMP7
GAPDH
C
CQ
D
LY2874455
FGFR
FRS2/GRB2/SOS/GAB1
PI3K
AKT
mTOR
ULK1/ATG13 complex
Autophagy initiation
LC3
Autophagosome
Lysosome
E
NIH3T3
LY2874455(uM) 0 0.2 0.5 1 2 5
p62
LC3I
LC3II
F
AZD4547 0 10 50 100 nM
G
TNF-α
IL1β
Relative mRNA expression
H
iNOS
I
IL6

S4 Figure

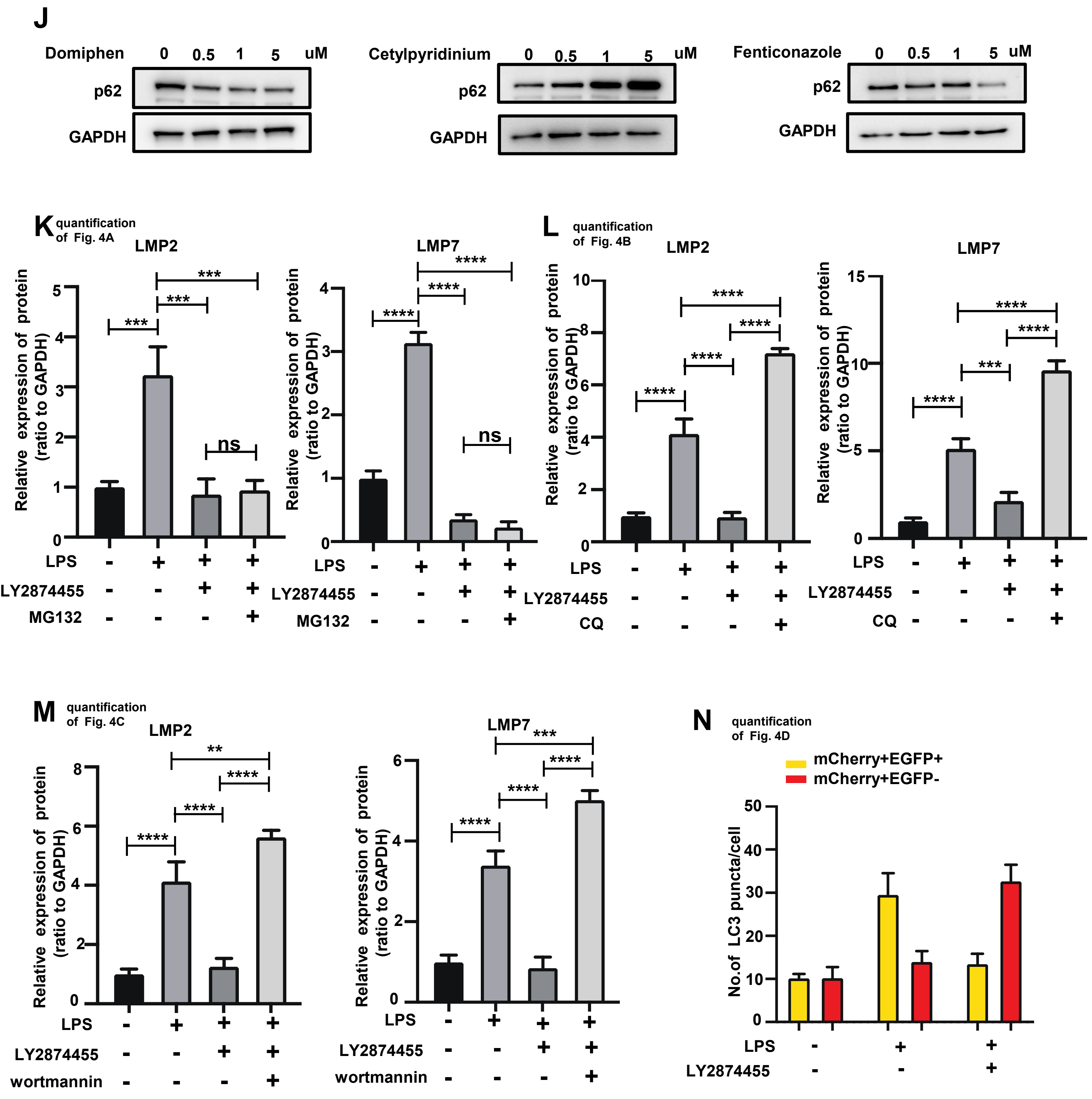
J
Domiphen 0 0.5 1 5 uM
p62
GAPDH
Cetylpyridinium 0 0.5 1 5 uM
p62
GAPDH
Fenticonazole 0 0.5 1 5 uM
p62
GAPDH
K quantification of Fig. 4A
LMP2
LMP7
Relative expression of protein (ratio to GAPDH)
ns
LPS
LY2874455
MG132
L quantification of Fig. 4B
LMP2
LMP7
CQ
M quantification of Fig. 4C
LMP2
LMP7
wortmannin
N quantification of Fig. 4D
mCherry+EGFP+
mCherry+EGFP-
No.of LC3 puncta/cell

S4 Figure

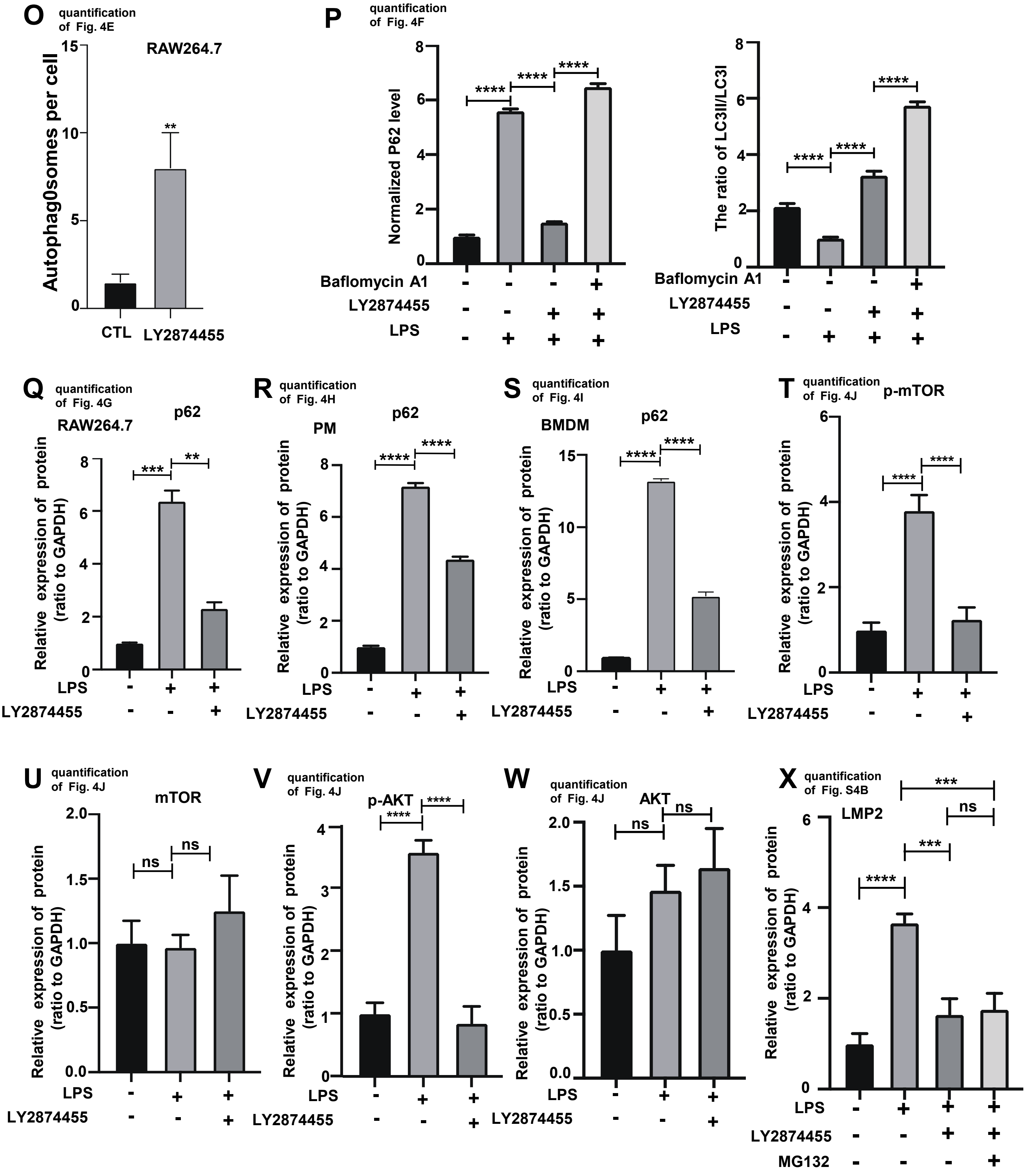

O
quantification of Fig. 4E
RAW264.7
Autophag0somes per cell
**
CTL
LY2874455
P
quantification of Fig. 4F
Normalized P62 level
The ratio of LC3II/LC3I
****
Baflomycin A1
LY2874455
LPS
Q
quantification of Fig. 4G
RAW264.7
p62
R
quantification of Fig. 4H
PM
p62
S
quantification of Fig. 4I
BMDM
p62
T
quantification of Fig. 4J
p-mTOR
Relative expression of protein (ratio to GAPDH)
***
**
U
quantification of Fig. 4J
mTOR
ns
V
quantification of Fig. 4J
p-AKT
W
quantification of Fig. 4J
AKT
X
quantification of Fig. S4B
LMP2
MG132

S4 Figure

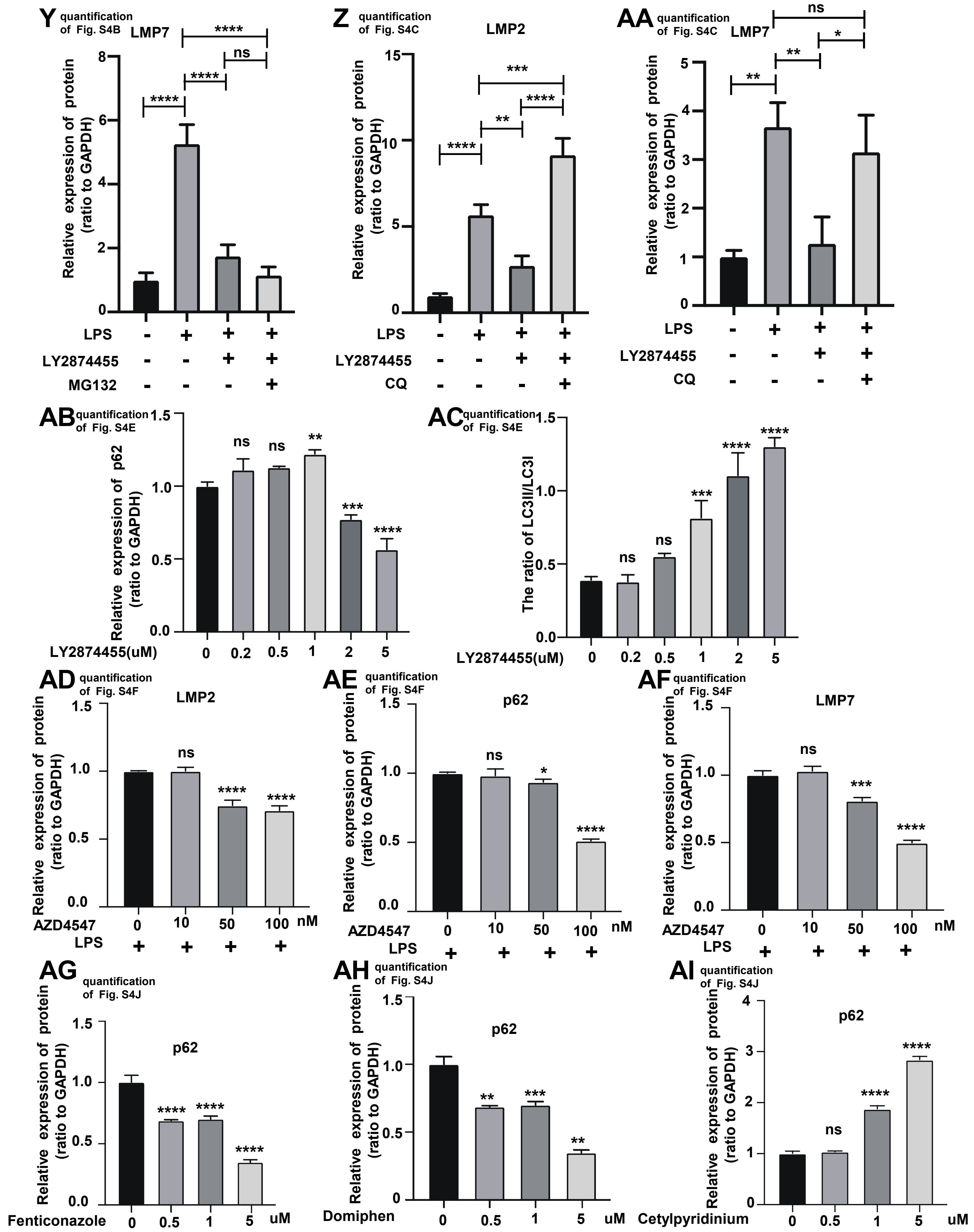
Y
quantification of Fig. S4B
LMP7
Relative expression of protein (ratio to GAPDH)
LPS
LY2874455
MG132
Z
quantification of Fig. S4C
LMP2
CQ
AA
quantification of Fig. S4C
LMP7
AB
quantification of Fig. S4E
Relative expression of p62 (ratio to GAPDH)
LY2874455(uM)
AC
quantification of Fig. S4E
The ratio of LC3II/LC3I
AD
quantification of Fig. S4F
LMP2
AZD4547
nM
AE
quantification of Fig. S4F
p62
AF
quantification of Fig. S4F
LMP7
AG
quantification of Fig. S4J
p62
Fenticonazole
uM
AH
quantification of Fig. S4J
Domiphen
AI
quantification of Fig. S4J
Cetylpyridinium
