# Supplementary material for: Pharmacological induction of autophagy reduces inflammation in macrophages by degrading immunoproteasome subunits
Source: PLoS Biol. 2024 Mar 6;22(3):e3002537. doi: 10.1371/journal.pbio.3002537 (PMC10917451; doi:10.1371/journal.pbio.3002537)
Supplement: S1 Table — (PDF) [file pbio.3002537.s007.pdf]

Supplementary S1 Table

Primers used for qRT-PCR assays in this study.

mRNA	forward (5'-3')	reverse (5'-3')
hIL6	AATAACCACCCCTGACCCAAC	ACATTTGCCGAAGAGCCCT
hTNF-α	GAGGCCAAGCCCTGGTATG	CGGGCCGATTGATCTCAGC
hGAPDH	ACAACTTTGGTATCGTGGAAGG	GCCATCACGCCACAGTTTC
mIL-17	TTTAACTCCCTTGGCGCAAAA	CTTTCCCTCCGCATTGACAC
mTNF-a	AATGGCCTCCCTCTCATCAGTT	CGAATTTTGAGAAGATGATCTGAGTGT
miNOS	CAGCACAGGAAATGTTTC AGC	TAGCCA GCG TAC CGG ATG A
mIL6	GTTGCCTTCTTGGGA CTG AT	CTGGCTTTGTCTTTCTTGTTAT
mGAPDH	AGGTCGGTGTGAACGGATTTG	TGTAGACCATGTAGTTGAGGTCA

Dilutions for antibodies used in this study.

Primary antibody	company	dilution
anti-iNOS	CST	WB(1:1000)
anti-PSMB1	Proteintech	WB(1:1000)
anti-PSMB2	Huabio	WB(1:1000)
anti-PSMB5	Affinity	WB(1:1000)
anti-LMP2	Abcam	WB(1:1000)
anti-LMP7	Abcam	WB(1:1000)
anti-LMP10	Abcam	WB(1:1000)
anti-mTOR	Proteintech	WB(1:1000)
anti-phospho-mTOR (Ser248)	Abcam	WB(1:1000)
anti-AKT	CST	WB(1:1000)
anti-phospho-Akt (Ser473)	CST	WB(1:1000)
anti-ATG7	Abcam	WB(1:10000)
anti-SQSTM/p62	Proteintech	WB(1:5000)
anti-Phospho-NF-κB p65 (Ser536)	Bioss	WB(1:1000)
anti-Phospho-IκB-α (Ser32)	ABclone	WB(1:1000)
TRIF	ABclone	WB(1:1000)
MYD88	ABclone	WB(1:1000)
TRAF6	ABclone	WB(1:1000)
TAK1	ABclone	WB(1:1000)
TAB2	ABclone	WB(1:1000)
anti-NBR1	Abcam	WB(1:1000)
anti-GAPDH	Proteintech	WB(1:5000)
HRP-conjugated Anti-Rabbit	BBI Life Sciences	WB(1:5000)
HRP-conjugated Anti-mouse	BBI Life Sciences	WB(1:5000)
