# Supplementary material for: Pharmacological induction of autophagy reduces inflammation in macrophages by degrading immunoproteasome subunits
Source: PLoS Biol. 2024 Mar 6;22(3):e3002537. doi: 10.1371/journal.pbio.3002537 (PMC10917451; doi:10.1371/journal.pbio.3002537)

Fig. 1

Fig. 1H:

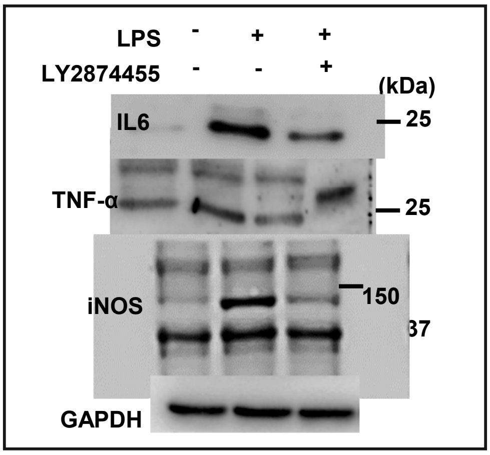

Fig. 1J:

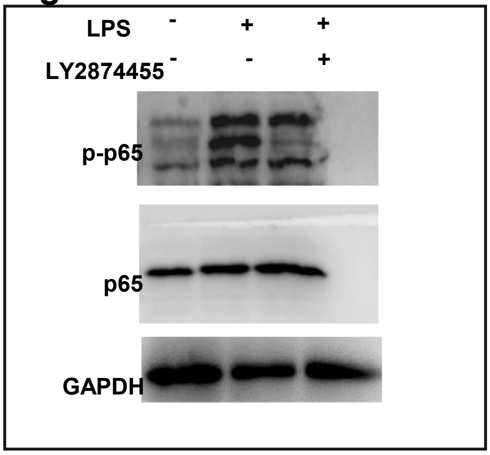

Fig. 1H:

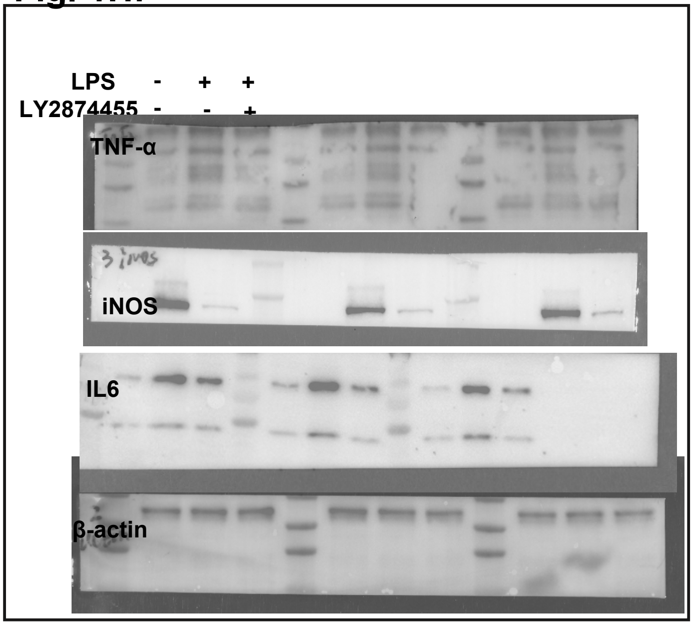

Fig. 1L:

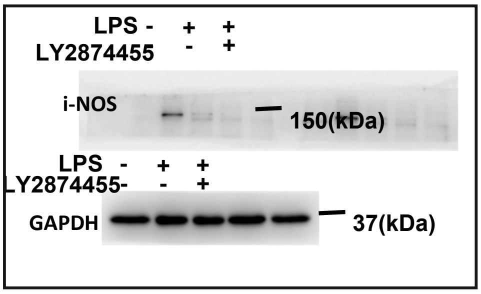

Fig. 1Q:

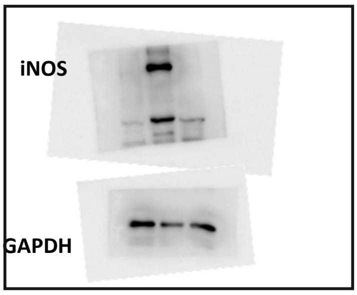

Fig. S2

Fig. S6L:

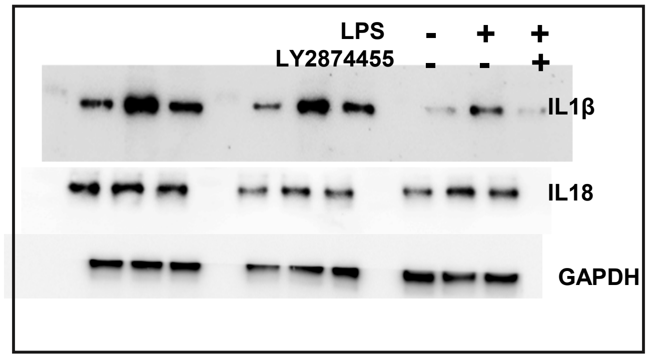

Fig.3

Fig. 3D:

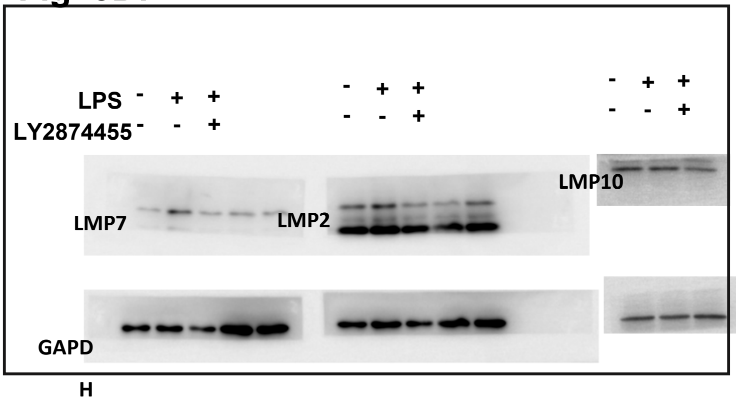

Fig. 3E:

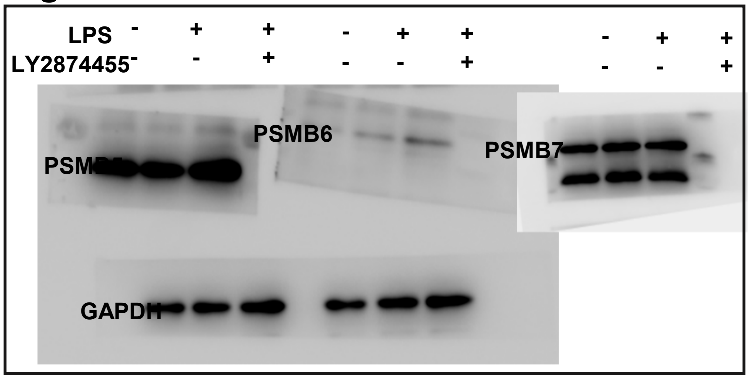

Fig. 3F:

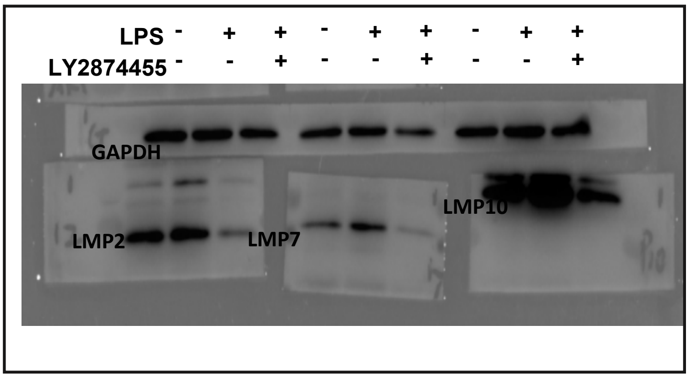

Fig. 3G:

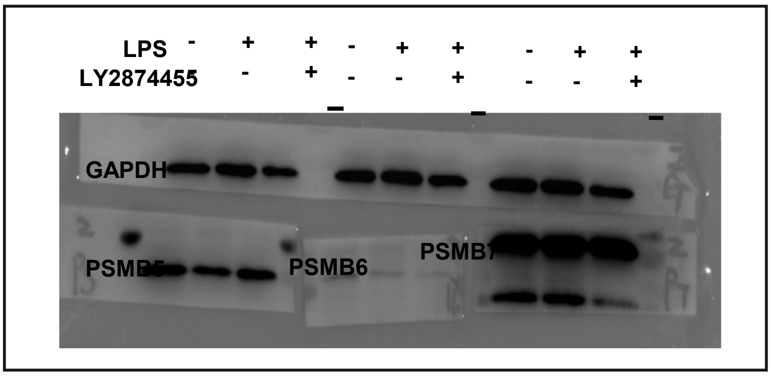

Fig. S3C:

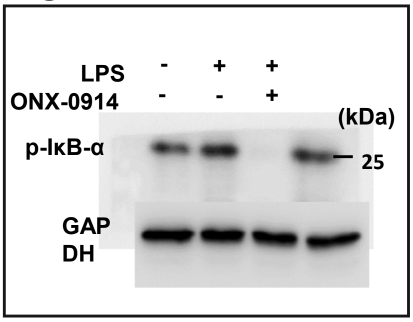

Fig. S3D-S3E:

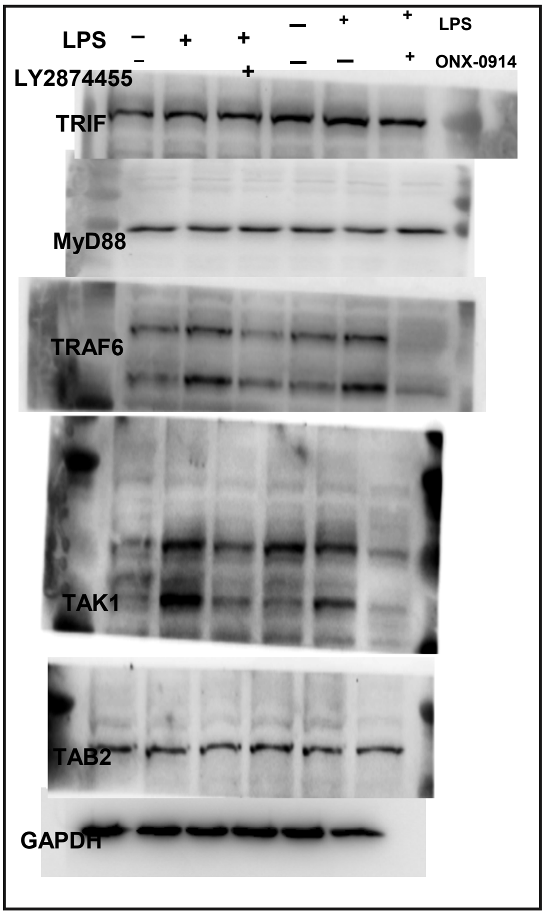

Fig. S2G:

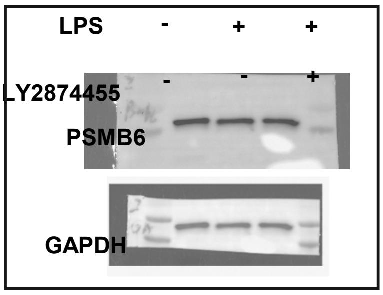

Fig.4

Fig. 4A:

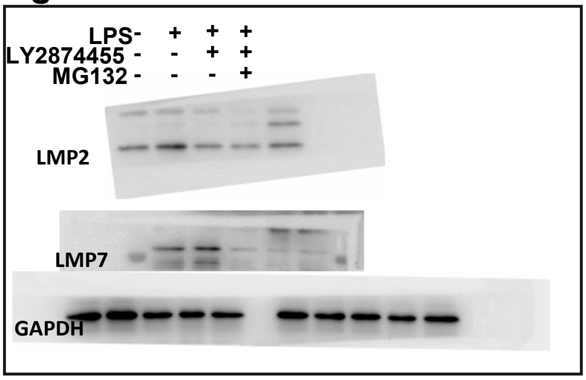

Fig. 4B:

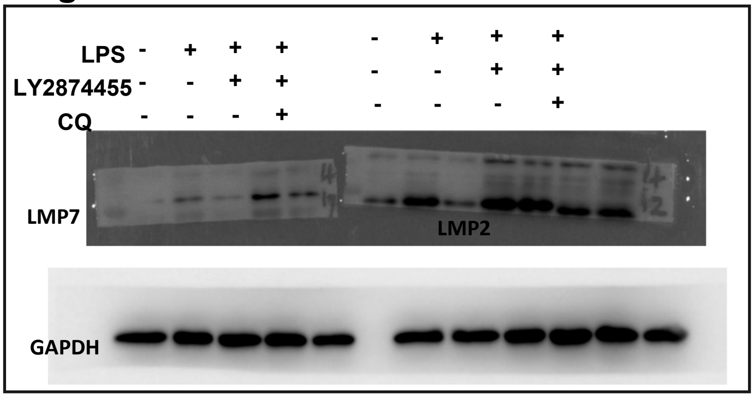

Fig. 4C:

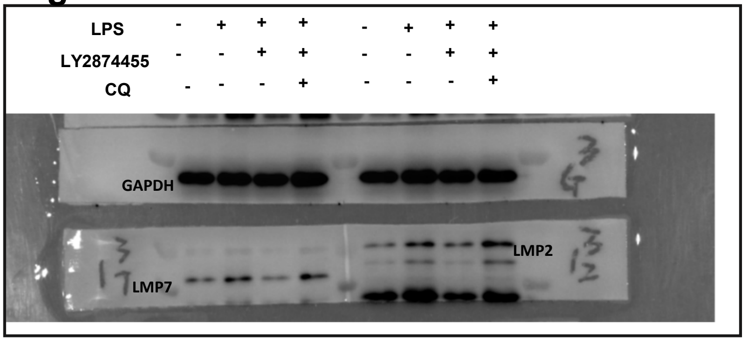

Fig. 4J:

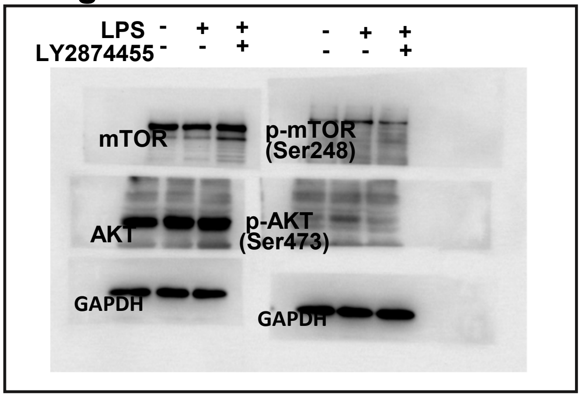

Fig. 4F:

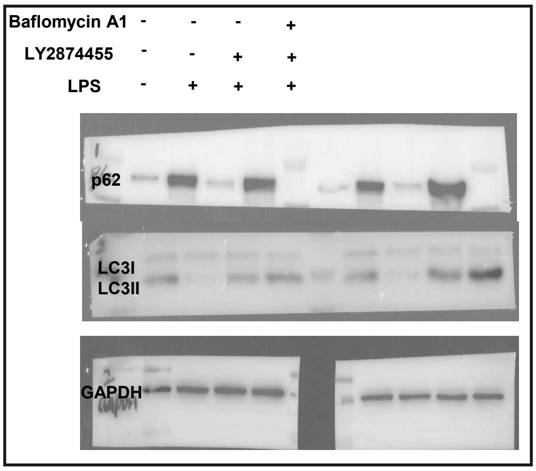

Fig. 4G:

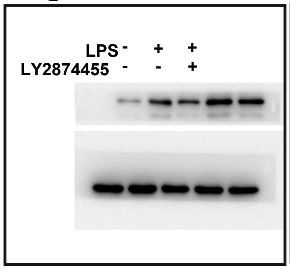

Fig. 4H:

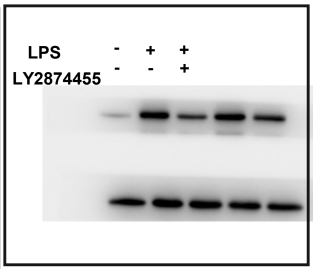

Fig. 4I:

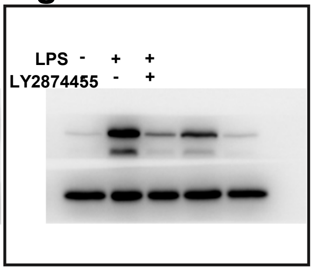

Fig. S4B

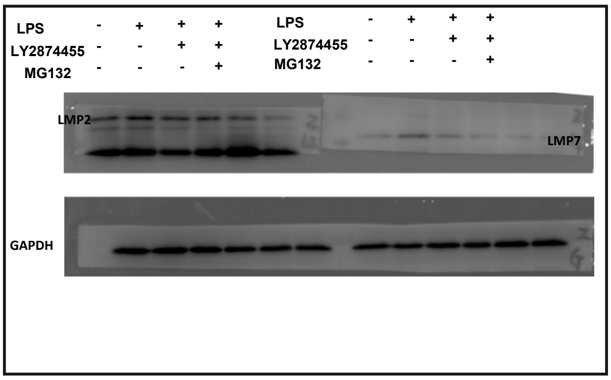

Fig. S4C

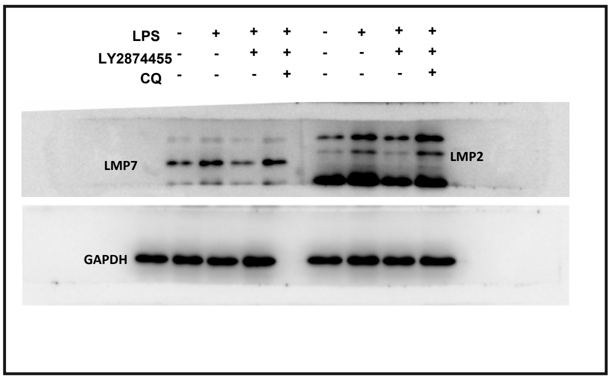

Fig. S4E

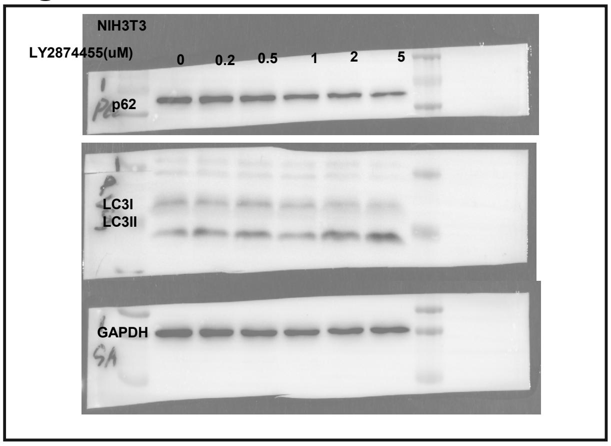

Fig. S4F

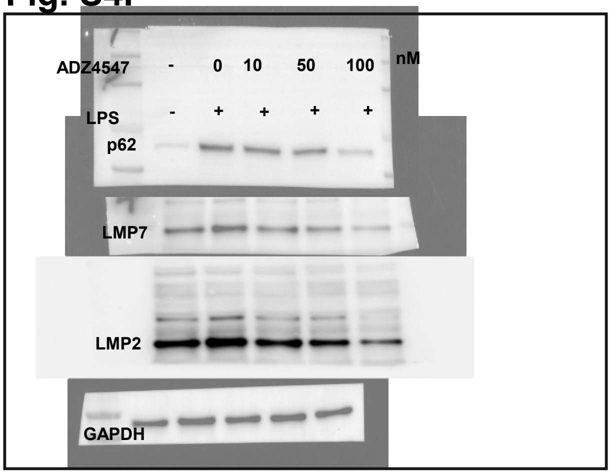

Fig. S4J

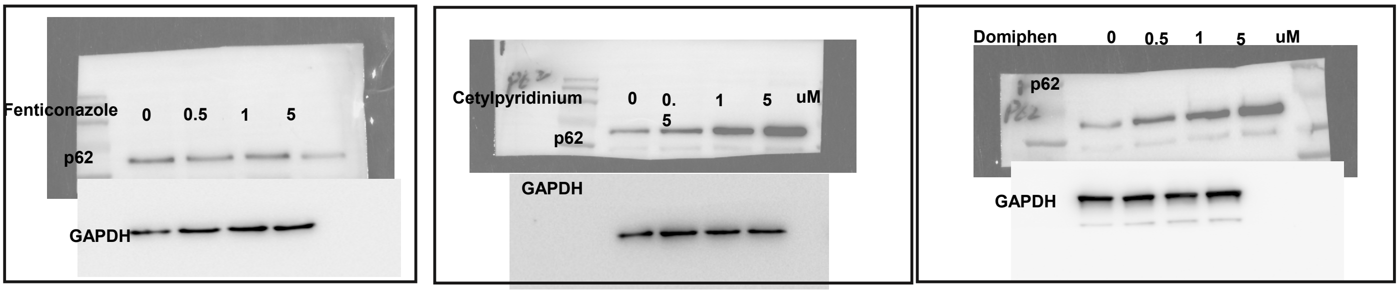

Fig. 5

Fig. 5 A:

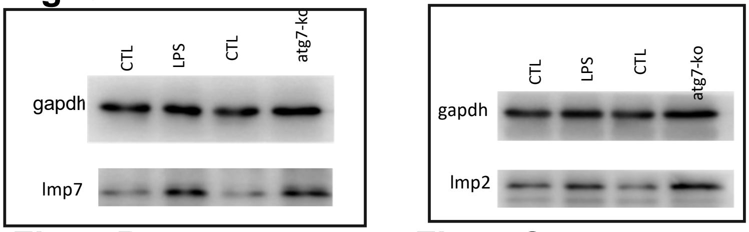

Fig. 5 B:

Fig. 5 C:

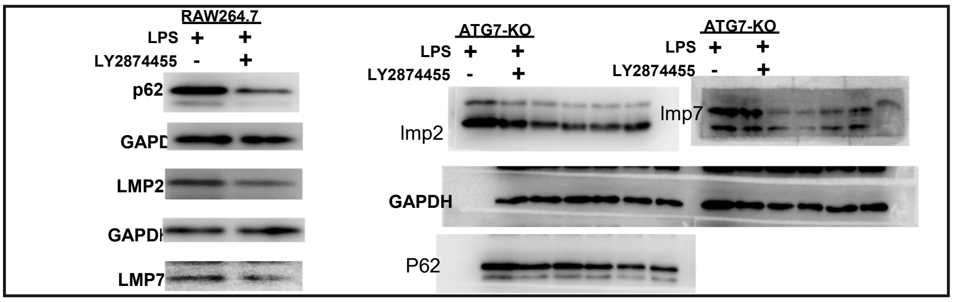

Fig. 5 G:

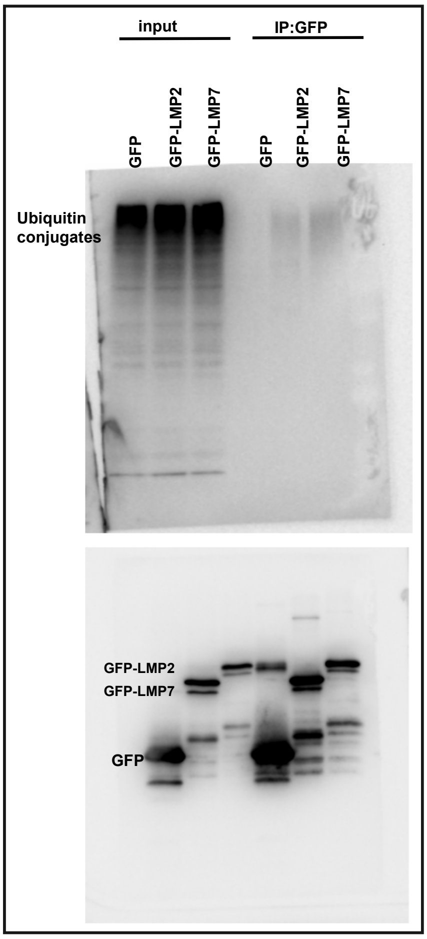

Fig. 5 H,I:

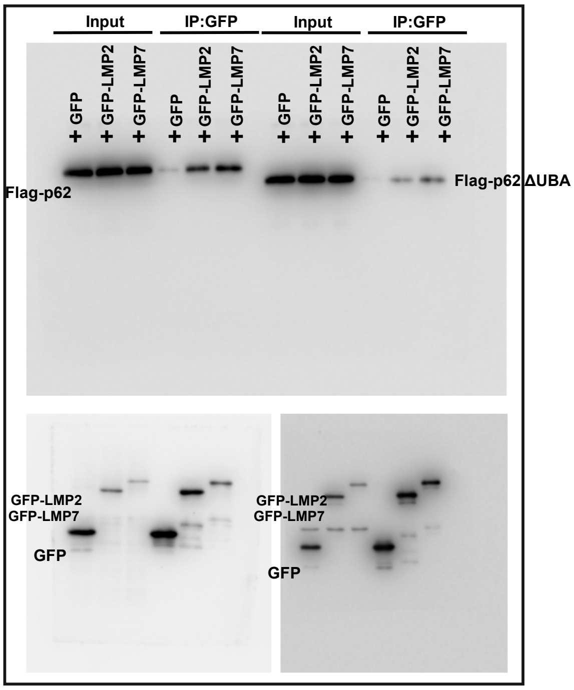

Fig. S5 A:

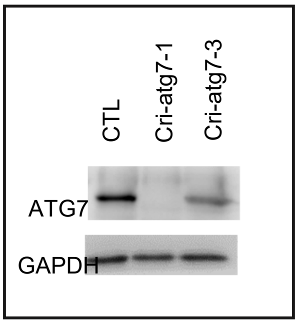

Fig. S5 B:

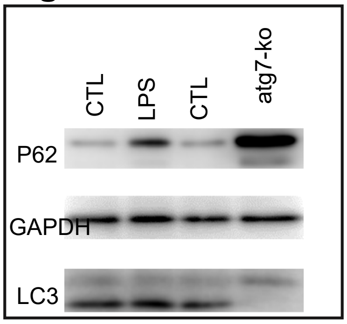

Fig. S6

Fig.S6C:

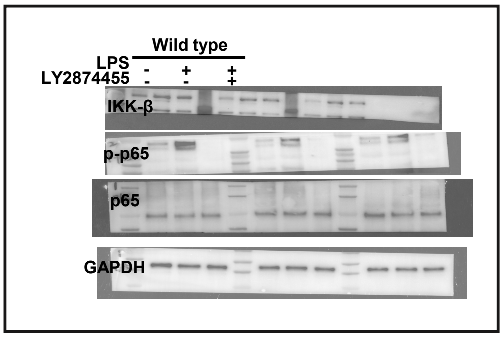

Fig. S6E:

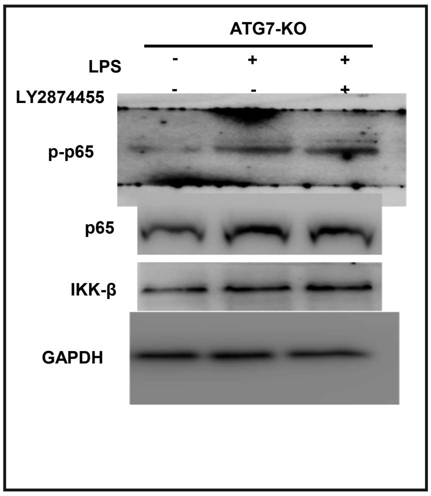

Fig. S6G:

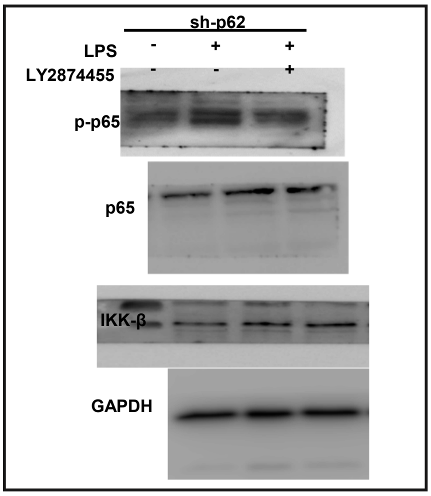

Fig. S6I:

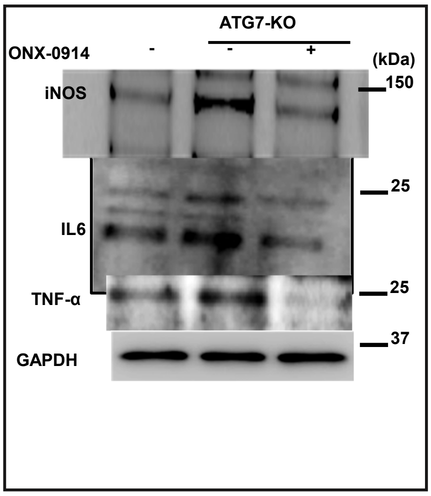

Supplement: S1 Raw Images — (PDF) [file pbio.3002537.s010.pdf]
