# Supplementary material for: Pharmacological induction of autophagy reduces inflammation in macrophages by degrading immunoproteasome subunits
Source: PLoS Biol. 2024 Mar 6;22(3):e3002537. doi: 10.1371/journal.pbio.3002537 (PMC10917451; doi:10.1371/journal.pbio.3002537)
Supplement: Attachment — Submitted filename: PBIOLOGY-D-23-00322_R1_EG.pdf [file pbio.3002537.s011.pdf]

PLOS Biology

Selective Autophagy of the Immunoproteasome Suppresses Innate Inflammatory Signalling

--Manuscript Draft--

**Manuscript Number:**	PBIOLOGY-D-23-00322R1
**Full Title:**	Selective Autophagy of the Immunoproteasome Suppresses Innate Inflammatory Signalling
**Short Title:**	a novel mechanism of inflammation-suppression by selective autophagy of the immunoproteasome
**Article Type:**	Initial Research Submission
**Keywords:**	
**Abstract:**	Defective autophagy is linked to proinflammatory diseases. However, the mechanisms by which autophagy limits inflammation remain elusive. Here, we found that the pan-FGFR inhibitor LY2874455 efficiently activated autophagy and suppressed innate inflammation in macrophages stimulated by lipopolysaccharide (LPS). Multiplex proteomic profiling identified the immunoproteasome, a specific isoform of the 20s constitutive proteasome, as substrates that are degraded by selective autophagy. SQSTM1/p62 was found to be a selective autophagy-related receptor that mediated this degradation. Autophagy deficiency or p62 knockdown blocked the effects of LY2874455, leading to the accumulation of immunoproteasomes and increases in inflammatory reactions. Inflammation in autophagy-deficient macrophages could be reversed by immunoproteasome inhibitors, confirming the pivotal role of immunoproteasome turnover in autophagy-mediated inflammation suppression. In mice, LY2874455 protected against LPS-induced acute lung injury and dextran sulfate sodium-induced colitis and caused low levels of proinflammatory cytokines and immunoproteasomes. These findings suggested that selective autophagy of the immunoproteasome was a key regulator of signaling via the innate immune system.
**Additional Information:**	
**Question**	**Response**
**Competing Interests**<br><br>You are responsible for recognizing and disclosing on behalf of all authors any competing interest that could be perceived to bias their work, acknowledging all financial support and any other relevant financial or non-financial competing interests.<br><br>Do any authors of this manuscript have competing interests (as described in the PLOS Policy on Declaration and Evaluation of Competing Interests)?<br><br>**If yes**, please provide details about any	The authors have declared that no competing interests exist.

and all competing interests in the box below. Your response should begin with this statement: *I have read the journal's policy and the authors of this manuscript have the following competing interests:*<br><br>**If no** authors have any competing interests to declare, please enter this statement in the box: "*The authors have declared that no competing interests exist.*"<br><br>* typeset<br><br>This statement is required for submission and **will appear in the published article** if the submission is accepted. Please make sure it is accurate and that any funding sources listed in your Funding Information later in the submission form are also declared in your Financial Disclosure statement.	
**Ethics Statement**<br><br>You must include an Ethics Statement in the Methods section of your manuscript if your study involved:<br>• Human participants<br>• Human specimens or tissue<br>• Vertebrate animals or cephalopods<br>• Vertebrate embryos or tissues<br>• Field research<br><br>**Not including a statement when required will delay the review process.**<br><br>General guidance is provided below. Please consult the guidelines on human subjects research and animal research for detailed instructions.	I confirm that I have read and understood PLOS’ requirements for an Ethics Statement.

Powered by Editorial Manager® and ProduXion Manager® from Aries Systems Corporation

**Format for specific study types**<br><br>**Human Subject Research (involving human participants and/or tissue)**<br>• Give the name of the institutional review board or ethics committee that approved the study<br>• Include the approval number and/or a statement indicating approval of this research<br>• Indicate the form of consent obtained (written/oral) or the reason that consent was not obtained (e.g. the data were analyzed anonymously)<br><br>**Animal Research (involving vertebrate animals, embryos or tissues)**<br>• Provide the name of the Institutional Animal Care and Use Committee (IACUC) or other relevant ethics board that reviewed the study protocol, and indicate whether they approved this research or granted a formal waiver of ethical approval<br>• Include an approval number if one was obtained<br>• If the study involved *non-human primates*, add *additional details* about animal welfare and steps taken to ameliorate suffering<br><br>**Field Research**<br><br>Include the following details if this study involves the collection of plant, animal, or other materials from a natural setting:<br>• Field permit number<br>• Name of the institution or relevant body that granted permission<br><br>Please check the box to confirm your understanding of this policy, then read and agree to one of the statements.	
Please respond:<br>as follow-up to "**Ethics Statement**<br><br>You must include an Ethics Statement in the Methods section of your manuscript if	My study requires an ethics statement. I confirm I have included my statement in the Methods section of my uploaded manuscript file.

your study involved:<br>• Human participants<br>• Human specimens or tissue<br>• Vertebrate animals or cephalopods<br>• Vertebrate embryos or tissues<br>• Field research<br><br>**Not including a statement when required will delay the review process.**<br><br>General guidance is provided below. Please consult the guidelines on human subjects research and animal research for detailed instructions.	

Powered by Editorial Manager® and ProduXion Manager® from Aries Systems Corporation

**Format for specific study types**<br><br>**Human Subject Research (involving human participants and/or tissue)**<br>• Give the name of the institutional review board or ethics committee that approved the study<br>• Include the approval number and/or a statement indicating approval of this research<br>• Indicate the form of consent obtained (written/oral) or the reason that consent was not obtained (e.g. the data were analyzed anonymously)<br><br>**Animal Research (involving vertebrate animals, embryos or tissues)**<br>• Provide the name of the Institutional Animal Care and Use Committee (IACUC) or other relevant ethics board that reviewed the study protocol, and indicate whether they approved this research or granted a formal waiver of ethical approval<br>• Include an approval number if one was obtained<br>• If the study involved *non-human primates*, add *additional details* about animal welfare and steps taken to ameliorate suffering<br><br>**Field Research**<br><br>Include the following details if this study involves the collection of plant, animal, or other materials from a natural setting:<br>• Field permit number<br>• Name of the institution or relevant body that granted permission<br><br>Please check the box to confirm your understanding of this policy, then read and agree to one of the statements. "	
**Data Availability**<br><br>PLOS journals require authors to make all data underlying the findings described in their manuscript fully available, without restriction and from the time of	Yes - all data are fully available without restriction

publication, with only rare exceptions to address legal and ethical concerns (see the PLOS Data Policy and FAQ for further details). When submitting a manuscript, authors must provide a Data Availability Statement that describes where the data underlying their manuscript can be found.<br><br>Your answers to the following constitute your statement about data availability and will be included with the article in the event of publication. **Please note that simply stating 'data available on request from the author' is not acceptable.** ***If, however, your data are only available upon request from the author(s), you must answer "No" to the first question below, and explain your exceptional situation in the text box provided.***<br><br>Do the authors confirm that all data underlying the findings described in their manuscript are fully available without restriction?	
Please describe where your data may be found, writing in full sentences. **Your answers should be entered into the box below and will be published in the form you provide them, if your manuscript is accepted.** If you are copying our sample text below, please ensure you replace any instances of XXX with the appropriate details.<br><br>• If your data are all contained within the paper and/or Supporting Information files, please state this in your answer below. For example, "All relevant data are within the paper and its Supporting Information files."<br>• If your data are held or will be held in a public repository, include URLs, accession numbers or DOIs. For example, "All XXX files are available from the XXX database (accession	All relevant data are within the paper and its Supporting Information files.

number(s) XXX, XXX)." If this information will only be available after acceptance, please indicate this by ticking the box below.<br>• If neither of these applies but you are able to provide details of access elsewhere, with or without limitations, please do so in the box below. For example:<br><br>"Data are available from the XXX Institutional Data Access / Ethics Committee for researchers who meet the criteria for access to confidential data."<br><br>"Data are from the XXX study whose authors may be contacted at XXX."<br><br>* typeset	
Additional data availability information:	

Manuscript Click here to access/download;Manuscript;Combined manuscript.pdf

Selective Autophagy of the Immunoproteasome Suppresses Innate Inflammatory Signalling

Jiao Zhou[1,2,#], Chunxia Li[1,#], Meng Lu[1], Gaoyue Jiang[1], Shanze Chen[3], Huihui Li[4], and Kefeng Lu[1,*]

[1]Department of Neurosurgery, State Key Laboratory of Biotherapy, West China Hospital, Sichuan University and The Research Units of West China, Chinese Academy of Medical Sciences, Chengdu 610041, China

[2]National Clinical Research Center for Geriatrics, West China Hospital, Sichuan University and Collaborative Innovation Center of Biotherapy, Chengdu 610041, China

[3]Department of Respiratory and Critical Care Medicine, First Affiliated Hospital of Southern University of Science and Technology, Second Clinical Medical College of Jinan University, Shenzhen People's Hospital, Shenzhen Institute of Respiratory Diseases, Shenzhen 518020, China

[4]West China Second University Hospital, Sichuan University, Chengdu 610041, China

[#]These authors contributed equally

[*]Correspondence: Kefeng Lu (lukf@scu.edu.cn)

Abstract

Defective autophagy is linked to proinflammatory diseases. However, the mechanisms by which autophagy limits inflammation remain elusive. Here, we found that the pan-FGFR inhibitor LY2874455 efficiently activated autophagy and suppressed innate inflammation in macrophages stimulated by lipopolysaccharide (LPS). Multiplex proteomic profiling identified the immunoproteasome, a specific isoform of the 20s constitutive proteasome, as substrates that are degraded by selective autophagy. SQSTM1/p62 was found to be a selective autophagy-related receptor that mediated this degradation. Autophagy deficiency or p62 knockdown blocked the effects of LY2874455, leading to the accumulation of immunoproteasomes and increases in inflammatory reactions. Inflammation in autophagy-deficient macrophages could be reversed by immunoproteasome inhibitors, confirming the pivotal role of immunoproteasome turnover in autophagy-mediated inflammation suppression. In mice, LY2874455 protected against LPS-induced acute lung injury and dextran sulfate sodium-induced colitis and caused low levels of proinflammatory cytokines and immunoproteasomes. These findings suggested that selective autophagy of the immunoproteasome was a key regulator of signaling via the innate immune system.

Introduction

Inflammation is an essential immune response that enables survival during pathogenic microorganism infection or tissue injury[1,2]. Various extrinsic and intrinsic inflammatory stimuli include infectious microorganisms (such as viruses and bacteria), cancer cells or dead cells[3-6]. Although inflammation is a vital component of a healthy immune response in the host, rampant inflammation results in various pathologies such as sepsis, rheumatoid arthritis, acute lung injury, chronic respiratory diseases, inflammatory bowel disease (IBD), cancer, aging, or even death[7-16]. One very recent example of inflammation-related disease is coronavirus disease 2019 (COVID-19), which is caused by severe acute respiratory syndrome coronavirus 2 (SARS-CoV-2). Serious COVID-19 is associated with inflammation, with an emphasis on cytokine storm syndrome[17-25]. Uncontrolled macrophage and monocyte activation in response to SARS-CoV-2 infection causes a rampant subsequent inflammatory response that results in acute respiratory distress syndrome and end-organ injuries including lung fibrosis[20,26,27]. Anti-inflammatory factor therapies have been used to inhibit the development of cytokine storms in COVID-19 patients[28-32]. In addition to viruses, pathogenic bacterial infection is also a robust cause of inflammation and death. Approximately 50% of patients in intensive care units (ICUs) develop severe sepsis caused by bacterial infection, and a 29% mortality rate has been shown in infected patients[33-35]. Mortality is mostly attributed to the cytotoxic effects of lipopolysaccharide (LPS) (endotoxin), a component of the outer membrane of gram-negative bacteria[36-39]. Various and large numbers of inflammatory factors, such as tumour necrosis factor-α (TNF-α), interleukin-1β (IL-1β), and interleukin-6 (IL-6), and high levels of intracellular reactive oxygen species (ROS), are substantially induced in LPS-stimulated macrophages[40-44].

Regulation of macrophage activity is essential for maintaining immune homeostasis. Receptor tyrosine kinases (RTKs) including colony-stimulating factor 1 receptor (CSF-1R), recepteur d'origine nantais (RON) receptor, myeloid-epithelial-reproductive tyrosine kinase (MERTK), Tyro3, Axl, and Mer (TAM) receptors, epidermal growth factors receptors (EGFRs) and fibroblast growth factor receptors (FGFRs) are found to be expressed in macrophages and involved in macrophage function and differentiation[45]. CSF-1R positively

regulates the functions and differentiation of tumor-associated macrophages[46]. Activation of RON receptor tyrosine kinase exerts dual stimulatory and suppressive effects on LPS-induced production of inflammatory cytokines in macrophages[47]. MERTK is strongly upregulated during migration and differentiation of macrophages and its signaling facilitates polarizing macrophages toward anti-inflammatory M2 phenotypes[48]. TAM receptors play important roles in the anti-inflammatory responses through modulating the function of macrophages[49-51]. EGFR is expressed and activated in colonic macrophages in mice and patients with colitis, and promotes DSS-induced acute colitis[52]. EGFR is also expressed in liver macrophages and is required for induction of cytokines such as IL-6[53]. During M2 polarization elicited by IL-4, the expression of EGFR is down-regulated. IL-4 or chitin can induce a higher level of M2 polarization in peritoneal macrophages when EGFR is deleted in myeloid cells[54]. EGFR in gastric tissue macrophages is phosphorylated upon infection with Helicobacter pylori and activates NF-κB and MAPK1/3 pathways to induce cytokine production[55]. Stimulating of human macrophages results in phosphorylation of EGFR, which promotes polarization of M2 macrophages[56]. Both M2 macrophage activation and M1 macrophage activation are promoted by EGFR in myeloid cells during colitis-associated carcinogenesis[57]. LPS promotes high levels of cell surface expression of EGFR in macrophages and its signaling is required for macrophage activation during sepsis[58,59]. High fat diet increases the expression of EGFR in adipose tissue macrophages (ATMs) and the induced EGFR mediates activation of ATMs, which promotes obesity and insulin resistance[60]. Compared with EGFRs, there are less studies on FGFRs in macrophages. Expression of FGFR-1 in infiltrating macrophages is detected during renal inflammation[61]. Activation of astrocytes in the normal and injured neocortex is delayed by astrocytic FGFRs, and disrupting astrocytic FGFRs results in reduced scar size without affecting neuronal survival[62]. FGFR inhibitor AZD4547 suppresses the proliferative activity of macrophages in lung tissue and further exerts a protective effect against excessive inflammatory damage in septic mice[63]. FGFR3 deficiency in myeloid cells promoted macrophage chemotaxis partly through activation of NF-κB/CXCR7 pathway, suggesting the role of FGFR3 in synovial macrophage recruitment and synovitis, an inflammation-related arthritis[64].

The immunoproteasome is an important mediator of inflammation in immune cells such as macrophages and non-immune cells, and its expression is triggered by inflammatory signals[65-77]. The immunoproteasome is a specific isoform of the 20s constitutive proteasome that is quickly formed (in about 20 minutes) by replacing the β1, β2, and β5 subunits of the 20s proteasome with different counterparts known as β1i (low molecular mass polypeptide 2, LMP2), β2i (multi-catalytic endopeptidase complexlike-1, MECL-1, also known as LMP10) and β5i (low molecular mass polypeptide 2, LMP7)[65,66,70,78]. Originally, the immunoproteasome was found to hydrolyse proteins into peptides loaded into the MHC-I complex as antigens[67,79,80]. Apart from antigen processing, the immunoproteasome has been associated with macrophage polarization[81], brain inflammation[82-84], diabetic nephropathy[85], the production of inflammatory cytokines[86-90], myometrium inflammation associated with preterm labor[91], diet-induced atherosclerosis[92], various inflammatory and autoimmune diseases[86,88,93-98], colitis and colitis-associated cancers[99-102], viral infection[103-110], the activation of lymphocytes[111,112], and cardiac hypertrophy[113,114]. The necessity of lowering excessive immunoproteasomes has been highlighted, as immunoproteasome inhibitors have become promising drug candidates for various diseases, such as hematologic malignancies, autoimmune, and inflammatory diseases[115-122]. However, it is unknown whether and how degradation of the immunoproteasome is chieved.

Macroautophagy/autophagy is a catabolic pathway for bulk transport through double-layered membrane vesicles (autophagosomes) and the eventual degradation of intracellular components in lysosomes[123-126]. The autophagy pathway is highly conserved among eukaryotic organisms and plays fundamental roles in various physiological and pathological conditions [127-129]. The autophagy pathway is a dynamic process that functions to maintain cellular homeostasis through the selective degradation of cytoplastic cargoes such as toxic protein aggregates, unnecessary or damaged organelles and intracellular pathogens[130-133]. Autophagy dysfunction has been linked to several proinflammatory diseases, such as rheumatoid arthritis, lupus, and inflammatory bowel disease (IBD)[134-137]. Germline variations in the autophagy genes ATG16L1, NOD2, IRGM, ATG5 and ATG7 is associated with chronic

inflammatory diseases including Crohn's disease[138-142]. However, the mechanisms through which autophagy limits inflammation remain unclear.

Thus, it is important to clarify whether and how autophagy targets the immunoproteasome for degradation and the involvement of this degradation in limiting inflammation. In the present study, we identified the pan-FGFR inhibitor LY2874455 as an effective anti-inflammatory treatment through the activation of autophagy. We utilized LPS-induced inflammatory responses in macrophages as a functional screen and identified the pan-FGFR inhibitor LY2874455 as an effective inhibitor of inflammation. We found that the stimulation of autophagy by LY2874455 promoted the autophagic degradation of the immunoproteasomes in LPS-induced macrophages. LY2874455 inhibited the AKT-mTOR axis to activate autophagy, and autophagic degradation of the immunoproteasome was mediated by the receptor SQSTM1/p62. LY2874455-activated autophagy dramatically inhibited the production of inflammatory cytokines, and blocking autophagy with inhibitors, ATG7 knockout, or knockdown of the receptor p62 reversed this inhibition. The protective role of LY2874455-induced autophagic degradation of the immunoproteasome was verified by in vivo mouse models with acute lung injury induced by LPS or with inflammatory bowel disease induced by dextran sulfate sodium (DSS). These findings suggested that selective autophagic degradation of the immunoproteasome was a pivotal regulator of innate inflammatory signaling.

Results

The pan-FGFR inhibitor LY2874455 effectively suppressed inflammation in LPS-stimulated macrophages.

Because numerous diseases are caused by rampant inflammation, we screened for chemicals with potential suppressive effects on inflammation in the LPS-treated macrophage cell line RAW264.7. Nitrous oxide (NO) levels were measured because NO is generated at increased levels during inflammation[143]. RAW264.7 cells were cultured in 96-well plates and treated with LPS and in total 5618 compounds including FDA-approved drug library (MedChemExpress, Cat. HY-L022, 2669 compounds), PI3K/Akt/mTOR compound library (MedChemExpress, Cat. HY-L015, 456 compounds) and

kinase inhibitor library (MedChemExpress, Cat. HY-L009, 2493 compounds). NO concentration in culture supernatant was measured in a high-throughput manner with three replicated wells for each compound. We found that LY2874455 was the most effective anti-inflammatory chemical by measuring NO levels (only 27 chemicals, including LY2874455, are shown) (**Fig. S1A, B**). LY2874455 ((R)-(E)-2-(4-(2-(5-(1-(3,5-dichloropyridin-4-yl) ethoxy)-1H-indazol-3yl) vinyl)-1H-pyrazol-1-yl) ethanol) (**Fig. 1A**) is a novel pan-FGFR inhibitor that shows good tolerability and activity in solid organ cancer patients [144,145]. In addition to suppressing NO generation in LPS-stimulated RAW264.7 macrophages without affecting cell viabilities (**Fig. 1B** and **Fig. S1C, D**), LY2874455 also reduced the levels of reactive oxygen species (ROS, shown by DCFH-DA staining) in LPS-stimulated RAW264.7 cells (**Fig. 1C, D**). The levels of proinflammatory cytokines, such as tumor necrosis factor-α (TNF-α) and interleukin-6 (IL-6), and inducible nitric oxide synthase (iNOS) were dramatically induced by LPS in RAW264.7 macrophages, and LY2874455 completely reversed this effect (**Fig. 1E-H**). Inflammation suppression by LY2874455 was repeatedly observed in peritoneal macrophages (PMs) and bone marrow-derived macrophages (BMDMs) obtained from mice[146,147] (**Fig. S1E-L**) and in human THP-1 monocytes (**Fig. S1M, N**). NF-κB signaling is a classic proinflammatory regulator that mediates the induction of various inflammatory cytokines[148-151]. The IκB kinase (IKK) complex, which contains IKK-α, IKK-β, and IKK-γ, activates the inflammatory transcription factor NF-κB. IKK-β phosphorylates inhibitory IκBα to promote its polyubiquitination and proteasomal degradation, and then the classic p65/p50 heterodimer is freed to translocate to the nucleus and mediate the transcription of NF-κB target genes, including various cytokines. The LPS-induced protein levels of IKK-β in RAW264.7 cells were reduced by LY2874455 (**Fig. 1I**). The activated form of p65 (phosphorylated) and its translocation into the nucleus were also reversed by LY2874455 in LPS-stimulated RAW264.7 cells (**Fig. 1J, K**).

Taken together, these results demonstrated that the pan-FGFR inhibitor LY2874455 effectively suppressed the inflammatory responses in LPS-stimulated macrophages.

LY2874455 reduced the protein levels of immunoproteasomes in LPS-stimulated macrophages.

To determine the mechanism by which LY2874455 suppresses inflammatory responses, mass spectrometry-based proteomics analysis was performed on RAW264.7 macrophages treated with LPS and LY2874455 (**Fig. 2A**, upper panel). Clustering of the altered proteins suggested that the immunoproteasome-specific subunits were downregulated by LY2874455 treatment (arrow pointed out in **Fig. 2A**, lower panel). Under inflammatory conditions such as LPS stimulation, the immunoproteasome-specific subunits β1i/LMP2, β2i/MECL-1/LMP10 and β5i/LMP7 replaced the original β1, β2, and β5 subunits of the 20s proteasome, leading to the formation of immunoproteasomes (schemed in **Fig. 2B, C**). The function of immunoproteasome in NF-κB pathway was confirmed by the observation that immunoproteasome inhibitor ONX-0914 efficiently blocked the nucleus translocation of p65 (**Fig. S2A**). The function of immunoproteasome in NF-κB pathway was suggested to be not through degrading p-IκB-α (**Fig. S2B**). Instead, immunoproteasome may function in NF-κB pathway through regulating the upstream signaling steps that promotes the phosphorylation of IκB-α (**Fig. S2C**). The exact mechanism of immunoproteasome in regulating the NF-κB pathway has been elusive. We analyzed the NF-κB pathway at presence of LY2874455 or ONX-0914 by detecting the components function in NF-κB pathway. Results showed that LY2874455 and ONX-0914 similarly caused the reduction of protein levels of TRAF6 and TAK1, while showed no effect on other components (**Fig. S2D, E**). These results suggested that immunoproteasome may regulating the TRAF6-TAK1 phase in NF-κB pathway. Further, the same effects of LY2874455 and ONX-0914 suggested that LY2874455 suppressed the inflammation pathway through immunoproteasome.

We confirmed the proteomics results and found that LY2874455 indeed reduced the protein levels of the immunoproteasome subunits LMP2, LMP7, and LMP10 in LPS-stimulated RAW264.7 macrophages (**Fig. 2D**). In contrast, the protein levels of constitutive proteasome subunits (PSMB1, PSMB2, and PSMB5) in LPS-stimulated RAW264.7 macrophages were not affected by LY2874455 (**Fig. 2E**). Similar results were also obtained in peritoneal macrophages (PM) isolated from mice (**Fig. 2F, G**). Moreover, the catalytic activities of immunoproteasome subunits (LMP2, LMP7 and LMP10) in LPS-stimulated RAW264.7 macrophages were also reduced by LY2874455 (**Fig.

2H and **Fig. S2F**). The role of the immunoproteasome in mediating inflammation and the specific inhibitor ONX-0914 have been reported. We confirmed that ONX-0914 effectively suppressed the inflammatory responses in LPS-stimulated RAW264.7 macrophages by measuring the expression of the proinflammatory cytokines TNF-α and IL-6 (**Fig. 2I, J**). More importantly, additional treatment with LY2874455 did not further repress inflammation in ONX-0914-treated macrophages (**Fig. 2I, J**). This result indicated that LY2874455 repressed inflammation though immunoproteasome inhibition, which was probably mediated by lowering the protein levels of immunoproteasomes.

LY2874455 activated autophagy.

We then tried to determine the mechanism by which LY2874455 reduces the protein levels of immunoproteasome subunits (equivalent to immunoproteasomes) since their mRNA levels were not influenced by LY2874455 treatment (data not shown). The ubiquitin–proteasome and autophagy–lysosome systems have been reported to be the two mechanisms of cytoplastic degradation. In the two intracellular degradation pathways (the proteasome pathway and the autophagy pathway), large substrates are generally targeted for autophagic degradation. We blocked these two degradation pathways using proteasome inhibitor MG132 and autophagy inhibitors chloroquine (CQ) and wortmannin (targeting Class III PI3K, VPS34 that uses PtdIns as a substrate to produce PtdIns3P, a lipid material for autophagosome formation[152,153]), and then analyzed the protein levels of immunoproteasome subunits. Western blot analysis showed that MG132 cannot inhibit the LY2874455-induced reductions in LMP2 and LMP7 (**Fig. 3A** and **Fig. S3A, B**), whereas the decrease induced by LY2874455 was markedly reversed by CQ or wortmannin (**Fig. 3B, C** and **Fig. S3A, C**). This result thus suggested that immunoproteasome degradation induced by LY2874455 was more dependent on autophagy than the ubiquitin–proteasome system.

These findings also suggested that LY2874455 can induce autophagy. Autophagic flux assays were conducted by measuring the dot signal of the fusion protein mCherry-EGFP-LC3[154]. The high intensity of the mCherry signal and low GFP signal induced by LY2874455 indicated that LY2874455

activated autophagy (**Fig. 3D**). Transmission electron microscopy showed many more autophagosomes in RAW264.7 macrophages treated with LY2874455 than in control cells (**Fig. 3E** and **Fig. S3D**). p62/SQSTM1 is an autophagic receptor that recruits substrates (usually ubiquitinated substrates) into autophagosomes, and it is degraded together with the substrates after the autophagosome fuses with the lysosome. The protein levels of p62 were gradually reduced with the addition of increasing amounts of LY2874455 (**Fig. 3F**). LPS stimulation caused the accumulation of p62 (**Fig. 3G**), indicating that LPS blocked autophagy, which was consistent with the observation that LPS reduced autophagic flux (**Fig. 3D**, yellow signal in LPS cells). LY2874455 effectively reduced the protein levels of p62 in LPS-stimulated RAW264.7 macrophages (**Fig. 3G**). In peritoneal macrophages (PMs) and bone marrow-derived macrophages (BMDMs) isolated from mice, LY2874455 also reduced the protein levels of p62 (**Fig. 3H, I**). These results confirmed the role of LY2874455 in activating autophagy.

FGFRs are transmembrane RTKs and activated by FGF to form dimers, leading to the activation of downstream FRS2 complex and subsequent activation of PI3K/AKT/mTOR signaling pathway[155-158]. Activated mTOR then phosphorylates and inhibits autophagy initiating ULK1 kinase complex, leading to suppression of autophagy[153,159]. We found that LY2874455 reduced the levels of AKT-mTOR signaling, as shown by the low levels of mTOR and AKT phosphorylation (**Fig. 3J**). This LY2874455-mediated inhibition of the AKT-mTOR signaling pathway further indicated its ability to activate autophagy. LY2874455 suppresses the FGFR activities and the downstream PI3K/AKT/mTOR signaling pathway, leading to the release and activation of autophagy (schemed in **Fig. S3E**).

LY2874455 promoted the degradation of immunoproteasomes through autophagy and the selective receptor p62.

For further validation of the effect of LY2874455 on promoting the degradation of immunoproteasomes through autophagy, ATG7, an essential gene in autophagy, was knocked out in RAW264.7 cells, which resulted in blocked autophagy, an increase in p62 and deficiency in the activated form of LC3 (LC3-II) (**Fig. S4A**). Then, the protein levels of the immunoproteasome subunits LMP2 and LMP7 were analyzed. In ATG7-knockout (ATG7-KO)

RAW264.7 cells, the protein levels of LMP2 and LMP7 were increased compared to those in WT cells (**Fig. 4A**). While the protein levels of LMP2 and LMP7 were reduced by LY2874455 in WT cells, this effect was abolished in ATG7-KO cells (**Fig. 4B, C**). We examined the localization of autophagosomes and the immunoproteasome. The results showed that an autophagosome marker (LC3 dots) colocalized with LMP2 and LMP7(**Fig. 4 D**). Collectively, these results demonstrated that LY2874455 induced the selective capture and degradation of immunoproteasomes via autophagy.

Generally, selective targeting of substrates into autophagosomes is mediated by receptors that link cargos to autophagosomes and are degraded together with the substrates after autophagic transfer into lysosomes. We then analyzed three previously reported receptors and found that p62 behaved similarly to LMP2 and LMP7 in RAW264.7 cells, was increased by LPS treatment and decreased by further LY2874455 treatment (**Fig. 4E**). Knockdown of p62 in RAW264.7 cells led to increased protein levels of LMP2 and LMP7 (**Fig. 4F** and **Fig. S4B**). These results indicated p62 was the receptor protein for the autophagic degradation of immunoproteasomes. Substrate ubiquitination is necessary for selective targeting by the autophagy receptor p62. Indeed, LMP2 and LMP7 were ubiquitinated (**Fig. 4G**). Furthermore, LMP2 and LMP7 could interact with p62, and this interaction was shown to be dependent on the UBA domain (ubiquitin-associated domain for ubiquitin binding) of p62 (**Fig. 4H, I**). Thus, these results indicated p62 was the receptor that mediated the autophagic degradation of immunoproteasomes.

LY2874455 suppressed inflammation though the autophagy pathway.

Now that we have shown that LY2874455 induced the autophagic degradation of immunoproteasomes, we then examined whether the suppressive effect of LY2874455 on inflammation occurred through the autophagy pathway. The LPS-induced increase in ROS levels (shown by the DCFH-DA probe) in RAW264.7 cells was abolished by LY2874455; however, this abolishment was blocked by the autophagy inhibitor wortmannin (**Fig. 5A**). Moreover, increased mRNA levels of iNOS and IL-6 were observed in wortmannin-treated RAW264.7 cells (**Fig. 5B, C**), mouse-isolated primary macrophages (**Fig. 5D, E**) and bone marrow-derived macrophages (**Fig. S5A,**

B). These results suggested that LY2874455 suppressed inflammation through autophagy. This finding was further confirmed by the observation that ATG7 knockout or p62 knockdown led to increased levels of iNOS and IL-6 in the presence of LPS and LY2874455 (**Fig. 5F, G**). The effects of LY2874455 suppression on NF-κB pathway was also shown to be dependent on autophagy and receptor p62, as in autophagy- or p62-deficient macrophages, LY2874455 cannot suppress NF-κB pathway (**Fig. S5C-F**).

Together with the data showing that LY2874455 induced the autophagic degradation of immunoproteasomes, these results indicated the possibility that the increased inflammation in autophagy-deficient cells may be caused by the accumulation of autophagic substrate immunoproteasomes. The immunoproteasome-specific inhibitor ONX-0914 was then used to treat ATG7-KO RAW264.7 cells, and the expression levels of iNOS and the proinflammatory cytokines IL-6 and TNF-α were analyzed. The results showed that the immunoproteasome inhibitor ONX-0914 dramatically reduced inflammation in autophagy-deficient cells (**Fig. 5H-J** and **Fig. S5G**).

Hence, these results indicated that the suppressive effect of LY2874455 on inflammation was dependent on the induction of the autophagic degradation of immunoproteasomes.

LY2874455 suppressed lung and intestinal inflammation in mouse models.

After demonstrating the suppressive effect of LY2874455 on inflammation at the cellular level, we then analyzed whether LY2874455 could affect inflammatory mouse models with acute lung injury and septicopyemia induced by lipopolysaccharide (LPS)[160,161] or with inflammatory bowel disease induced by dextran sulfate sodium (DSS). Histological examination of the lungs of mice stimulated with LPS revealed considerable inflammatory cell infiltration, which is a typical symptom of lung inflammation (**Fig. 6A**). Notably, inflammatory cell infiltration was reduced by LY2874455 (**Fig. 6A**). The LPS-induced high levels of the proinflammatory cytokine IL-6 in the lung tissues of mice were also significantly reduced by LY2874455 (**Fig. 6B**). In addition, survival ~~analysis~~ of LPS-stimulated mice with or without LY2874455 treatment was measured. The survival rate was 100% in the control group but dropped notably in the LPS-treated group, while the survival rate was dramatically

increased in mice treated with LY2874455 (**Fig. 6C**). These results indicated that LY2874455 suppressed inflammation in mice with acute lung injury and septic pyemia induced by LPS and promoted survival in LPS-stimulated mice.

Furthermore, DSS-induced colitis model mice were used to analyze the potential gastrointestinal protective effects of LY2874455. The results showed that LY2874455 notably ameliorated the reduction in colon length in DSS-stimulated mice (**Fig. 6D**). To confirm the effect of LY2874455 on bowel inflammation and tissue injury, colonic sections of mice were subjected to histological examination. DSS caused severe damage to the mucosa of intestinal villi, and LY2874455 treatment efficiently reduced tissue damage (**Fig. 6E**). The expression levels of the proinflammatory cytokines IL-17 and TNF-α were reduced in colonic tissues from LY2874455-treated mice (**Fig. 6F, G**), which indicated the suppressive effect of LY2874455 on inflammation in colitis model mice. The inflammation-suppression effects of LY2874455 were in a wide-range manner, as it also showed suppressive effects on viral infection (**Fig. S6A-C**) and cell-aging induced inflammation (**Fig. S6D-H**).

Overall, these results confirmed the suppressive effect of LY2874455 on inflammation and LY2874455-mediated promotion of immunoproteasome turnover.

Discussion

Hypotheses on the precise regulation of inflammation by selective autophagy are fascinating; however, the underlying mechanism has been elusive. In the present study, we uncovered the role and mechanism of the pan-FGFR inhibitor LY2874455 in inflammation suppression. In macrophages stimulated with LPS, immunoproteasomes were induced and then triggered NF-κB-mediated inflammatory responses, leading to robust expression and generation of proinflammatory cytokines, ROS, and NO. In the presence of LY2874455, the autophagy pathway was activated, induced autophagosome targets and captured immunoproteasomes, and this effect was mediated by the selective receptor p62 recognizing ubiquitin signals on immunoproteasomes. This autophagic degradation reduced the abundance of immunoproteasomes and thus blocked the activity of NF-κB and eventually suppressed inflammation. Our work here reveals a novel mechanism by which

autophagy suppresses inflammation, which may substantially contribute to the development of new therapeutic approaches for inflammatory diseases.

Currently, it is unclear how the induction and autophagic degradation of immunoproteasomes are coordinated. We hypothesize that inflammatory stimuli such as LPS may induce the expression, assembly, and increased abundance of immunoproteasomes, while LPS inhibits the autophagy pathway, as shown by the increased protein levels of the receptor p62 (**Fig. 3F-I**). This result suggests that LPS not only induces the production of the immunoproteasome but also prohibits its autophagic degradation, which consequently causes the accumulation of the immunoproteasome and eventual rampant inflammation. The high level of immunoproteasome-induced inflammation provides efficacious signals to remind the host of toxic stimuli and thus activate host responses. However, the host benefits from intense inflammation-induced signals and simultaneously suffers from inflammation-induced damage, especially under severe/acute or chronic inflammatory conditions. Thus, the balance between moderate and controllable inflammation is essential in maintaining host health. Here, we propose a mechanism to explain how autophagy functions to break rampant inflammation. LY2874455, a novel pan-FGFR inhibitor, was shown to have good tolerability, absorbance, and activity in solid organ cancer patients in a phase I clinical study[145]. Thus, LY2874455 could be a good therapeutic candidate for inflammation-related diseases such as sepsis and Crohn's disease.

In addition to the regulation of acute inflammation, autophagy has been shown to positively alleviate chronic inflammation-related neurodegenerative disorders such as Alzheimer's disease, Parkinson's disease, and depression[162,163]. An overwhelming number of studies have connected autophagy in neurons to degenerative diseases; however, there are only sporadic hints of inflammatory connections via autophagy. Recently, it was shown that LPS could impair autophagy and relieve autophagic inhibition of the inflammatory machinery in microglia, a specific type of macrophage in the central nervous system[164-166]. Autophagy-deficient microglia show impaired phagocytic capacities and the overproduction of proinflammatory cytokines, which can lead to synaptic dysfunction, neuronal death, and the inhibition of neurogenesis[167]. It will be interesting to examine whether autophagic

degradation of the immunoproteasome functions as an inflammatory signal in microglia and whether and how much this dysregulation contributes to neurodegenerative diseases.

Cautiously, while in some cases defective autophagy causes excessive inflammation that leads to disease pathology, there are certain situations of viral infection in which elevated inflammation positively contributes to model animals. For example, myeloid-specific knockout of autophagy genes (RB1CC1, ATG5, ATG7, or ATG14) caused excessive inflammation in the lungs and facilitated resistance to influenza virus infection in animals[168]. Additionally, myeloid-specific knockout of several autophagy genes (RB1CC1, Belin1, ATG3, ATG5, ATG7, ATG14, or ATG16L) in animals subjected to herpesvirus infection results in elevated inflammation and prevented viral reactivation from latency[169]. We hypothesize that for pathogens that induce normal (slight increase above baseline) inflammation, autophagy deficiency results in elevated inflammation that is beneficial for host recognition and reaction to invaded pathogens, while for pathogens that induce high levels of inflammation, autophagy deficiency ~~causes~~ further increases inflammation, and this inflammation leads to more damage than is beneficial to the host. That is, although we showed that the autophagy-mediated reduction in the immunoproteasome is beneficial for animals with LPS-induced lung damage and DSS-induced inflammatory bowel damage, it is possible that in certain situations, autophagic degradation of the immunoproteasome may weaken proper host responses to pathogens. Therefore, whether and how autophagic degradation of the immunoproteasome functions in inflammation-related diseases such as aging, cancer, cardiovascular disease, neurodegeneration, and infections needs to be specifically examined.

The functions of selective autophagy in various conditions occur through receptors that specifically recognize certain types of substrates and concomitantly interact with lipidated LC3/Atg8 residing in the autophagosome membrane[170,171]. The degradation of substrates mostly indicates specific roles of autophagy[172]. The nature of autophagic substrates varies according to cell type and stimulating signals[130,173]. In our case, selective targeting of the immunoproteasome by autophagy explains the suppressive effect of autophagy on inflammation in macrophages. This conclusion was strengthened by the observation that autophagy blockade induced the

accumulation of immunoproteasomes accompanied by intense inflammatory reactions that could be completely reversed by the immunoproteasome inhibitor (**Fig. 5H-J**). This finding suggests that, at least in the contexts examined in this work, autophagic degradation of immunoproteasomes is mainly responsible for the role of autophagy in inflammation repression.

The receptor mediating autophagic degradation of the immunoproteasome is p62 but not NBR1 or Tollip, as shown in this study. One interesting question regarding receptor function in autophagy is why so many receptors exist. For example, p62, NBR1, Tollip, and OPTN function in aggrephagy (autophagy of protein aggregates); BNIP3L, FUNDC1, BNIP3, AMBRA1, BCL2LI3, FKBP8, CHDH, DISC1, PHB2, and cardiolipin function in mitophagy (autophagy of mitochondria); FAM134B, Sec62, RTN3, CCPG1 ATL3, and TEX264 function in ERphagy (autophagy of ER); and p62, NBR1, and PEX3 function in pexophagy (autophagy of peroxisomes)[130,173-175]. The redundancy of receptors may reflect the specific and meticulous regulation and function of autophagy in multicellular organisms. In other words, certain receptors functions in specifically defined types of cells. The clarification of where (cell types), when (activation or inhibition), and how (substrate targeting) autophagy functions may improve our knowledge of the intrinsic nature of autophagy as a dynamic and multifunctional process. The observation that immunoproteasome subunits are polyubiquitinated and their binding with p62 relies on the ubiquitin-binding domain of 62 indicates ubiquitin chains as the signal for immunoproteasome targeting by autophagosomes. Identifying the ubiquitin ligase(s) responsible for ubiquitination of the immunoproteasome may help to determine how ubiquitin ligase(s) directly or indirectly facilitate the specific recognition of the immunoproteasome by p62.

In conclusion, our findings described the selective autophagic degradation of the immunoproteasome in the context of aberrant inflammation in macrophages and supported a novel mechanism of the regulation of inflammation by selective autophagy. These data expanded the understand of autophagic cargo and, more importantly, explored the crosstalk between selective autophagy and inflammation, which should be meaningful in establishing effective therapies for inflammatory diseases.

Materials and Methods

Antibodies and Reagents

The following antibodies were purchased from Abcam: anti-SQSTM-1/p62 (ab109012), anti-LMP2 (ab3328), anti-LMP7 (ab3329), anti-Ubiquitin (ab7780), anti-NBR1 (ab55474), anti-ATG7 (ab133528), anti-Mtor (ab87540), anti-LMP10 (ab183506), anti-PSMB5 (ab90867), anti-GAPDH (60004-1-Ig), anti-p65 (10745-1-AP). Anti-PSMB1 (11749-1-AP) and anti-PSMB2 (15154-1-AP) were purchased from Proteintech. Those antibodies anti-LC3 (2775S), anti-phospho-AktSer473 (4060S), anti-phospho-mTORSer2448 (2971S), anti-p70S6K (9202S), anti-phospho-p70S6KThr421/Ser424 (9204S), anti-Akt (4685S), and anti-TNF-α (11948S) were from Cell Signaling Technology. The phospho-NF-kB p65 (Ser536) (bs-0982R) antibody and anti-IL6 (bs-0782R) was purchased from Bioss. Anti-phospho- -IκB-α (Ser32) (AP0707), anti-TRIF (A1155), anti-MYD88 (A0980), anti-TRAF6 (A16991), anti-TAK1 (A19077), anti-TAB2 (A9867) antibodies were from ABclonal Technology. The secondary antibodies HRP-conjugated anti-rabbit (D-110058) and HRP-conjugated anti-mouse(D-110087) were from BBI Life Sciences. Fluor-conjugated (488 and 594) secondary antibodies were purchased from Invitrogen. LY2874455 was from MCE and dissolved in phosphate-buffered saline (PBS). LPS (E. coli, serotype 055:B5) was purchased from Sigma. The H_2-2',7'-dichlorodihydrofluorescein diacetate (DCFH-DA) (S0033S) and NO detection Kit(S0021S) were purchased from Beyotime. Protease inhibitor cocktail was purchased from Sigma (Roche 11697498001). Polyinosinic-polycytidylic acid (Poly(I:C)) (HY-107202) was purchased from MCE

Macrophage culture and isolation

RAW264.7 macrophages were cultured in RPMI-1640(Sigma) with 10% fetal bovine serum (Gibico) and maintained in humidified 5% CO_2 atmosphere at 37°C. Peritoneal macrophage (PM) and bone marrow derived macrophages (BMDM) were isolated from C57BL/6J mice. 6-8 weeks aged mice were injected with 3% thioglycolate for 3 days in advance, and ascites and bone marrow were taken 3 days later. Cells separated from ascites were seeded in cell plates with RPMI1640 complete medium and were allowed to adhere for 4-6h. Adherent cells were considered as PM. Cells form bone marrow were

seeded in cell plates incubated with RPMI1640 complete medium with M-CSF(10ng/ml, Sigma) and were allowed to adhere for 7 days. Adherent cells were considered as BMDM.

Plasmid construction and DNA transfection

The target gene p62, LMP2, LMP7 were cloned into the pEGFP-C1 vector respectively. The target gene p62 and p62△UBA were cloned into the pFlag-CMV2 vector respectively. The target gene LC3 was cloned into the pLVX-mCherry-C1. The sh-RNA targeting p62 sequence (CCGGGTCTCTACAGATGCCAGAATCCTCGAGGATTCTGGCATCTGTAGAGACTTTTTG) was cloned into PLKO.1 vector. The guide RNA (gRNA) sequence (TCTGCCCACCCGCTTGACGT) of target gene ATG7 was cloned into the PX330 vector. 800bp sequences of upstream and downstream from the knockout site respectively and screen gene (puro) were cloned into the pUC19 vector. Endoxin-free plasmids were transfected into cells using jetprime reagent according to the protocol recommended by the manufacturer.

ROS Detection

For intracellular ROS measurement, the cells treated with LY2874455 and/or LPS for 24h were washed with PBS three times and incubated with 10 μM H2-DCFH-DA for 30 min at 37℃ in the dark. Then cells were washed three times and collected by scraping. Collected cells were resuspended in PBS and their fluorescence intensity corresponding to intracellular DCFH-DA levels was immediately read on a Becton Dickinson FACS. DCFH-DA levels were also visualized using fluorescence microscope.

Nitric Oxide (NO) concentration measurements

The level of NO derivative nitrite in culture medium was determined by Griess reaction as per the kit's manual (Beyotime). After reaction, the mixture is measured at 540nm with a multifunctional microplate reader (Biotek).

Quantitative real-time polymerase chain reaction analysis (qRT-PCR)

Total RNA was extracted from cells lines, primary cells, or tissues by using Fast Pure cell/Tissue Total RNA Isolation Kit (Nanjing Vazyme Biotek).

cDNAs were reverse transcribed from 1 μg of RNA by Superscript II Reverse Transcriptase kit (Nanjing Vazyme). The production was used as templates in quantitative real-time polymerase chain reactions (PCRs) by using SYBR Green reagent (Nanjing Vazyme) according to the protocol recommended by the manufacturer. The sequences of primers used for PCR amplification are shown in Supplementary Table S1. GAPDH served as internal normalization control.

Proteasome activity profiling

Subunit-selective fluorogenic peptide substrates were used to measure the catalytic activities of individual catalytic subunits by monitoring the rate of substrate hydrolysis over time. Briefly, protein lysates were prepared using passive lysis buffer (25mM Tris PH 7.5,100mM Nacl,5mM ATP,0.2% NP40,20%Glycerol) and diluted in 20S proteasome assay buffer (20 mM Tris-HCl, 0.5 mM EDTA, 0.035% SDS, pH 8.0). Enzyme reactions were initiated by the addition of proteasome substrates. Substrates and concentrations were used as following: Ac-ANW-AMC (β5i, 100μM), Ac-PAL-AMC (β1i activity, 100 μM) and Ac-Arg-Leu-Arg-AMC (β2i activity, 100 μM). The mixture was incubated at 37 °C for 2h. Fluorescence signals were measured using a ~~with a~~ multifunctional microplate reader (Biotek) at the excitation and emission wavelengths of 345 and 445 nm, respectively.

Immunoblotting

Cells or tissues were lysed with RIPA buffer with protease inhibitors (Roche 11697498001). The protein was quantified by BCA Protein Assay Kit (Sigma) according to the protocol recommended by the manufacturer. After quantified, protein precipitates were subjected to SDS-PAGE. Protein was transferred to PVDF membranes and blocked in 5% non-fat milk in Tris-HCl-buffered saline (TBS) with 0.1% Tween-20 (TBST) for 1 h at room temperature, after which primary antibody (Supplementary Table S2) was incubated overnight at 4 °C. Then membranes were incubated with the secondary antibody (Supplementary Table S2) at room temperature for 1h. Signals were acquired with a Chemidoc MP Imaging System (Bio-Rad).

Immunoprecipitation

Cells were lysed in a buffer containing 0.25% TX-100, 1% NP-40, 50 mM Tris-HCl, pH 8, 150 mM NaCl, and 1mMEDTA. Lysates were adjusted for concentration and volume and incubated with GFP-Beads (Shenzhen Sports Life Technology Company) at 4°C overnight. Then, the samples were centrifuged at 2000 rpm at 4°C for 3 min. The pellet was washed five times with wash buffer A [50 mM tris-HCl (pH 7.5), 100 mM sodium chloride, and 2mM EDTA]. Bound proteins were collected in RIPA buffer (50 mM Tris, pH 7.4, 150 mM NaCl, 0.1% SDS, 1% TritonX-100, and 0.5% sodium deoxycholate), and the precipitated proteins were subjected to SDS-PAGE.

Immunofluorescence

Cells grown on cover slips were fixed with 4% paraformaldehyde in PBS for 10 min followed by permeabilization with 0.1% TritonX100 for 10min and incubation with 3% BSA for 1 hour at room temperature. Cells were then incubated with primary antibody followed by a PBS wash and incubated with secondary antibody (donkey anti-mouse and anti-rabbit Alexa Fluor 488 or 594). DAPI was used to stain the DNA. Images were captured using the Zeiss LSM 880.

Mass spectrometry-based proteomics analysis

The original mass spectrometry proteomics data have been deposited to the ProteomeXchange Consortium via the PRIDE partner repository with the dataset identifier PXD038747 and are publicly available as of the date of publication.

Protein extraction. Sample was sonicated three times on ice using a high intensity ultrasonic processor (Scientz) in lysis buffer (8 M urea, 1% Protease Inhibitor Cocktail). The remaining debris was removed by centrifugation at 12,000 g at 4 °C for 10 min. Finally, the supernatant was collected and the protein concentration was determined with BCA kit according to the manufacturer’s instructions.

Trypsin digestion. For digestion, the protein solution was reduced with 5 mM dithiothreitol for 30 min at 56 °C and alkylated with 11 mM iodoacetamide for 15 min at room temperature in darkness. The protein sample was then diluted by adding 100 mM TEAB to urea concentration less than 2M. Finally,

trypsin was added at 1:50 trypsin-to-protein mass ratio for the first digestion overnight and 1:100 trypsin-to-protein mass ratio for a second 4 h-digestion.

LC-MS/MS analysis. The tryptic peptides were dissolved in solvent A (0.1% formic acid, 0.1% acetonitrile/in water), directly loaded onto a home-made reversed-phase analytical column (25cm length, 75/100 μm i.d.). Peptides were separated with a gradient from 6% to 24% solvent B (0.1% formic acid in acetonitrile) over 70 min, 24% to 35% in 14 min and climbing to 80% in 3 min then holding at 80% for the last 3 min, all at a constant flow rate of 450 nL/min on a nanoElute UHPLC system (Bruker Daltonics). The peptides were subjected to capillary source followed by the timsTOF Pro (Bruker Daltonics) mass spectrometry. The electrospray voltage applied was 1.75kV. Precursors and fragments were analyzed at the TOF detector, with a MS/MS scan range from 100 to 1700 m/z. The timsTOF Pro was operated in parallel accumulation serial fragmentation (PASEF) mode. Precursors with charge states 0 to 5 were selected for fragmentation, and 10 PASEF-MS/MS scans were acquired per cycle. The dynamic exclusion was set to 30 s.

Data processing protocol. The resulting MS/MS data were processed using MaxQuant search engine (v1.6.6.0). Tandem mass spectra were searched against the Mus_musculus_10090_SP_20191115 (17032 entries) concatenated with reverse decoy database. Trypsin/P was specified as cleavage enzyme allowing up to 2 missing cleavages. The mass tolerance for precursor ions was set as 20 ppm in first search and 20 ppm in main search, and the mass tolerance for fragment ions was set as 0.02 Da. Carbamidomethyl on Cys was specified as fixed modification, and acetylation on protein N-terminal and oxidation on Met were specified as variable modifications. FDR was adjusted to < 1%.

Cell aging induction

NIH3T3 cells were cultured in DMEM supplemented with 10% FBS. NIH3T3 cells were trypsinized and suspended in phosphate buffer solution (PBS) with H_2O_2 (400 μlM) at 37 °C for 45 min. H_2O_2 treatment was terminated by three times of washing. Then, the NIH3T3 cells were cultured with complete medium for three days.

Animal experiments

All procedures involved in animals were performed in conformity with relevant guidelines and regulations, and approved by the Ethics Committee of Sichuan University.

Induction of ALI (Acute lung injury)

Male mice aged 8 weeks were randomly divided into 3 groups (control group, LPS (10 mg/kg) group, LPS (10 mg/kg) + LY2874455 (1 mg/kg) group, 6 mice in each group. Mice were intragastric administration with either saline or LY2874455 (1mg/kg) at the same time as LPS injection (10mg/kg). Six hours after LPS injection, mice were killed and the lungs were removed. Lung tissues were subsequently used for quantitative real-time PCR assay and hematoxylineosin (HE) staining.

Induction of Sepsis

Male mice aged 8 weeks were randomly divided into 3 groups, 8 mice in each group. To evaluate the effect of LY2874455 on the survival of mice, endotoxemia was induced by a single i.p. injection of bacterial endotoxin (LPS, 35mg/kg), with i.g. administration of LY2874455 (1 mg/kg), at 2 h before the injection of LPS. Survival rates of animals were monitored up to 96 h after LPS challenge.

Induction of colitis

Colitis was induced in 8-week-old male mice by adding 3% dextran sulfate sodium (DSS; m.w. 36,000–50,000; MP Biomedicals, Solon, OH) to the drinking water, beginning on day 0. On the third day of DSS treatment, mice were subject to intragastric administration with either saline or LY2874455 (1mg/kg) for 5 days. Thereafter, mice were given regular drinking water. The body weight was measured daily throughout the experiment. Then mice were killed and the intestines were removed. Intestine tissues were subsequently used for quantitative real-time PCR assay, western blot assay, and hematoxylineosin (HE) staining.

Transmission electron microscopy

Macrophages after indicated treatments were washed three times for 15 minutes with 0.1M phosphate buffer, and then fixed in 2% aqueous osmium tetraoxide for 1 h followed by washing three times each for 15 minutes with deionized water. Samples were then dyed with 2% uranyl acetate for 30 minutes, and dehydrated through graded alcohols (50%–100%) and 100% acetone each for 15 minutes. After that, Samples were embedded in EPON 812 resin and cured for 24 hours at 37°C, 45°C, 60°C, respectively. Ultrathin (70 nm) sections were obtained by ultra-thin slicer machine and stained with 2% uranyl acetate and 0.3% lead citrate. Electron microscopy images of the samples were taken using Tecnai G2 Spirit transmission electron microscope (FEI Company). Representative images of at least three independent replicated experiments are shown.

Statistical analysis

All data shown in this study are representative results from at least three independent replicated experiments. Quantification data are expressed as means ± SD from at least three biological replicates. Statistical analyses were conducted using Prism5. Ordinary one-way or two-way ANOVA with Turkey's multiple comparisons test, or a two-tailed paired or unpaired Student's t-test were used to determine statistical significance (*: $P< 0.05$, **: $P< 0.01$, ***: $P< 0.001$, NS: no statistical difference).

Acknowledgements

We thank the Imaging Center of Sichuan University for assistance with imaging taking by confocal microscopy. This study was supported by grants from the National Key R&D Program of China under grant 2017YFA0506300 (to K.L.) and the National Natural Science Foundation under grant 32022020 (to K.L.) and 81902997 (to H.L.).

Disclosure statement

The authors declare no conflict of interests.

References:

1 Luo, A. *et al.*, The Microbiota and epigenetic Regulation of T Helper 17/Regulatory T Cells: in Search of a Balanced immune System. *FRONT IMMUNOL* **8** (2017).

2 Hawiger, J., Innate immunity and inflammation: A transcriptional paradigm. *IMMUNOL RES* **23** 99 (2001).

3 Artis, D. & Spits, H., The biology of innate lymphoid cells. *NATURE* **517** 293 (2015).

4 Isailovic, N., Daigo, K., Mantovani, A. & Selmi, C., Interleukin-17 and innate immunity in infections and chronic inflammation. *J AUTOIMMUN* **60** 1 (2015).

5 Rock, K. L., Lai, J. & Kono, H., Innate and adaptive immune responses to cell death. *IMMUNOL REV* **243** 191 (2011).

6 Waisman, A., Liblau, R. S. & Becher, B., Innate and adaptive immune responses in the CNS. *LANCET NEUROL* **14** 945 (2015).

7 Bektas, A. *et al.*, Age-associated changes in human CD4(+) T cells point to mitochondrial dysfunction consequent to impaired autophagy. *AGING* **11** 9234 (2019).

8 Franceschi, C., Garagnani, P., Parini, P., Giuliani, C. & Santoro, A., Inflammaging: a new immune-metabolic viewpoint for age-related diseases. *NAT REV ENDOCRINOL* **14** 576 (2018).

9 Ferrucci, L. & Fabbri, E., Inflammageing: chronic inflammation in ageing, cardiovascular disease, and frailty. *NAT REV CARDIOL* **15** 505 (2018).

10 Xavier, R. J. & Podolsky, D. K., Unravelling the pathogenesis of inflammatory bowel disease. *NATURE* **448** 427 (2007).

11 Sands, B. E. *et al.*, Ustekinumab as Induction and Maintenance Therapy for Ulcerative Colitis. *NEW ENGL J MED* **381** 1201 (2019).

12 Olivera, P., Danese, S., Jay, N., Natoli, G. & Peyrin-Biroulet, L., Big data in IBD: a look into the future. *NAT REV GASTRO HEPAT* **16** 312 (2019).

13 Andersson, U., Hyperinflammation: On the pathogenesis and treatment of macrophage activation syndrome. *ACTA PAEDIATR* **110** 2717 (2021).

14 Brands, X. *et al.*, Concurrent Immune Suppression and Hyperinflammation in Patients With Community-Acquired Pneumonia. *FRONT IMMUNOL* **11** 796 (2020).

15 Dickson, K. & Lehmann, C., Inflammatory Response to Different Toxins in Experimental Sepsis Models. *INT J MOL SCI* **20** (2019).

16 Schuller, S. S. *et al.*, Pentoxifylline modulates LPS-induced hyperinflammation in monocytes of preterm infants in vitro. *PEDIATR RES* **82** 215 (2017).

17 England, J. T. *et al.*, Weathering the COVID-19 storm: Lessons from hematologic cytokine syndromes. *BLOOD REV* 100707 (2020).

18 Mehta, P. *et al.*, COVID-19: consider cytokine storm syndromes and immunosuppression. *LANCET* **395** 1033 (2020).

19 Sinha, P., Matthay, M. A. & Calfee, C. S., Is a "Cytokine Storm" Relevant to COVID-19? *JAMA INTERN MED* **180** 1152 (2020).

20 Moore, J. B. & June, C. H., Cytokine release syndrome in severe COVID-19. *SCIENCE* **368** 473 (2020).

21 Webb, B. J. *et al.*, Clinical criteria for COVID-19-associated hyperinflammatory syndrome: a cohort study. *LANCET RHEUMATOL* **2** e754 (2020).

22 Liuzzo, G. & Patrono, C., COVID 19: in the eye of the cytokine storm. *EUR HEART J* **42** 150 (2021).

23 Ritchie, A. I. & Singanayagam, A., Immunosuppression for hyperinflammation in COVID-19: a double-edged sword? *LANCET* **395** 1111 (2020).

24 Ho, J. *et al.*, TOP1 inhibition therapy protects against SARS-CoV-2-induced lethal inflammation. *CELL* **184** 2618 (2021).

25 Hasan, D. *et al.*, A novel definition and treatment of hyperinflammation in COVID-19 based on purinergic signalling. *PURINERGIC SIGNAL* **18** 13 (2022).

26 McGonagle, D., Sharif, K., O'Regan, A. & Bridgewood, C., The Role of Cytokines including Interleukin-6 in COVID-19 induced Pneumonia and Macrophage Activation Syndrome-Like Disease. *AUTOIMMUN REV* **19** (2020).

27 Merad, M. & Martin, J. C., Pathological inflammation in patients with COVID-19: a key role for monocytes and macrophages. *NAT REV IMMUNOL* **20** 355 (2020).

28 Feldmann, M. *et al.*, Trials of anti-tumour necrosis factor therapy for COVID-19 are urgently needed. *LANCET* **395** 1407 (2020).

29 Huang, Q. *et al.*, Targeting inflammation and cytokine storm in COVID-19. *PHARMACOL RES* **159** (2020).

30 Safavi, F. & Nath, A., Silencing of immune activation with methotrexate in patients with COVID-19. *J NEUROL SCI* **415** (2020).
31 Wright, D. J. M., Prevention of the cytokine storm in COVID-19. *The Lancet. INFECTIOUS DISEASES* **21** 25 (2021).
32 Schulert, G. S., Can tocilizumab calm the cytokine storm of COVID-19? *LANCET RHEUMATOLOGY* **2** E449 (2020).
33 Timsit, J. *et al.*, Rationalizing antimicrobial therapy in the ICU: a narrative review. *INTENS CARE MED* **45** 172 (2019).
34 Bergsbaken, T., Fink, S. L. & Cookson, B. T., Pyroptosis: host cell death and inflammation. *NAT REV MICROBIOL* **7** 99 (2009).
35 Ramachandran, G., Gram-positive and gram-negative bacterial toxins in sepsis A brief review. *VIRULENCE* **5** 213 (2014).
36 Munford, R. S., Weiss, J. P. & Lu, M., Biochemical transformation of bacterial lipopolysaccharides by acyloxyacyl hydrolase reduces host injury and promotes recovery. *J BIOL CHEM* **295** 17842 (2020).
37 Jing, W., Lo Pilato, J., Kay, C. & Man, S. M., Activation mechanisms of inflammasomes by bacterial toxins. *CELL MICROBIOL* e13309 (2021).
38 Rathinam, V. A. K., Zhao, Y. & Shao, F., Innate immunity to intracellular LPS. *NAT IMMUNOL* **20** 527 (2019).
39 Pfalzgraff, A. & Weindl, G., Intracellular Lipopolysaccharide Sensing as a Potential Therapeutic Target for Sepsis. *TRENDS PHARMACOL SCI* **40** 187 (2019).
40 Kim, J. *et al.*, Metformin Suppresses Lipopolysaccharide (LPS)-induced Inflammatory Response in Murine Macrophages via Activating Transcription Factor-3 (ATF-3) Induction. *J BIOL CHEM* **289** 23246 (2014).
41 Arikawa, M., Kakinuma, Y., Noguchi, T., Todaka, H. & Sato, T., Donepezil, an acetylcholinesterase inhibitor, attenuates LPS-induced inflammatory response in murine macrophage cell line RAW 264.7 through inhibition of nuclear factor kappa B translocation. *EUR J PHARMACOL* **789** 17 (2016).
42 Naruishi, K. & Nagata, T., Biological effects of interleukin-6 on Gingival Fibroblasts: Cytokine regulation in periodontitis. *J CELL PHYSIOL* **233** 6393 (2018).
43 Zhang, G., Meredith, T. C. & Kahne, D., On the essentiality of lipopolysaccharide to Gram-negative bacteria. *CURR OPIN MICROBIOL* **16** 779 (2013).
44 Zamyatina, A. & Heine, H., Lipopolysaccharide Recognition in the Crossroads of TLR4 and Caspase-4/11 Mediated Inflammatory Pathways. *FRONT IMMUNOL* **11** (2020).
45 Huang, X. L. *et al.*, Role of receptor tyrosine kinases mediated signal transduction pathways in tumor growth and angiogenesis-new insight and futuristic vision. *INT J BIOL MACROMOL* **180** 739 (2021).
46 Ao, J. Y. *et al.*, Colony-Stimulating Factor 1 Receptor Blockade Inhibits Tumor Growth by Altering the Polarization of Tumor-Associated Macrophages in Hepatocellular Carcinoma. *MOL CANCER THER* **16** 1544 (2017).
47 Wang, M. H., Zhou, Y. Q. & Chen, Y. Q., Macrophage-stimulating protein and RON receptor tyrosine kinase: potential regulators of macrophage inflammatory activities. *SCAND J IMMUNOL* **56** 545 (2002).
48 Huelse, J. M., Fridlyand, D. M., Earp, S., DeRyckere, D. & Graham, D. K., MERTK in cancer therapy: Targeting the receptor tyrosine kinase in tumor cells and the immune system. *PHARMACOL THER* **213** 107577 (2020).
49 Lee, C. H. & Chun, T., Anti-Inflammatory Role of TAM Family of Receptor Tyrosine Kinases Via Modulating Macrophage Function. *MOL CELLS* **42** 1 (2019).
50 Myers, K. V., Amend, S. R. & Pienta, K. J., Targeting Tyro3, Axl and MerTK (TAM receptors): implications for macrophages in the tumor microenvironment. *MOL CANCER* **18** 94 (2019).
51 Dransfield, I. & Farnworth, S., Axl and Mer Receptor Tyrosine Kinases: Distinct and Nonoverlapping Roles in Inflammation and Cancer? *ADV EXP MED BIOL* **930** 113 (2016).
52 Lu, N. *et al.*, Activation of the epidermal growth factor receptor in macrophages regulates cytokine production and experimental colitis. *J IMMUNOL* **192** 1013 (2014).
53 Lanaya, H. *et al.*, EGFR has a tumour-promoting role in liver macrophages during hepatocellular carcinoma formation. *NAT CELL BIOL* **16** 972 (2014).
54 Zhao, G. *et al.*, Activation of Epidermal Growth Factor Receptor in Macrophages Mediates Feedback Inhibition of M2 Polarization and Gastrointestinal Tumor Cell Growth. *J BIOL CHEM* **291** 20462 (2016).
55 Hardbower, D. M. *et al.*, EGFR regulates macrophage activation and function in bacterial infection. *J CLIN INVEST* **126** 3296 (2016).
56 Ma, X. *et al.*, The pancreatic cancer secreted REG4 promotes macrophage polarization to M2 through EGFR/AKT/CREB pathway. *ONCOL REP* **35** 189 (2016).

57 Hardbower, D. M. *et al.*, EGFR-mediated macrophage activation promotes colitis-associated tumorigenesis. *ONCOGENE* **36** 3807 (2017).

58 Tang, J. *et al.*, EGFR signaling augments TLR4 cell surface expression and function in macrophages via regulation of Rab5a activation. *PROTEIN CELL* **11** 144 (2020).

59 Zhang, X. *et al.*, EGFR tyrosine kinase activity and Rab GTPases coordinate EGFR trafficking to regulate macrophage activation in sepsis. *CELL DEATH DIS* **13** 934 (2022).

60 Cao, S. *et al.*, EGFR-mediated activation of adipose tissue macrophages promotes obesity and insulin resistance. *NAT COMMUN* **13** 4684 (2022).

61 Rossini, M. *et al.*, Immunolocalization of fibroblast growth factor-1 (FGF-1), its receptor (FGFR-1), and fibroblast-specific protein-1 (FSP-1) in inflammatory renal disease. *KIDNEY INT* **68** 2621 (2005).

62 Kang, W. *et al.*, Astrocyte activation is suppressed in both normal and injured brain by FGF signaling. *PROC NATL ACAD SCI U S A* **111** E2987 (2014).

63 Huang, Y. *et al.*, Inhibition of Fibroblast Growth Factor Receptor by AZD4547 Protects Against Inflammation in Septic Mice. *INFLAMMATION* **42** 1957 (2019).

64 Kuang, L. *et al.*, FGFR3 deficiency enhances CXCL12-dependent chemotaxis of macrophages via upregulating CXCR7 and aggravates joint destruction in mice. *ANN RHEUM DIS* **79** 112 (2020).

65 Murata, S., Takahama, Y., Kasahara, M. & Tanaka, K., The immunoproteasome and thymoproteasome: functions, evolution and human disease. *NAT IMMUNOL* **19** 923 (2018).

66 McCarthy, M. K. & Weinberg, J. B., The immunoproteasome and viral infection: a complex regulator of inflammation. *FRONT MICROBIOL* **6** (2015).

67 Kimura, H., Caturegli, P., Takahashi, M. & Suzuki, K., New Insights into the Function of the Immunoproteasome in Immune and Nonimmune Cells. *J IMMUNOL RES* **2015** (2015).

68 Ferrington, D. A. & Gregerson, D. S., Immunoproteasomes: Structure, Function, and Antigen Presentation. *PROG MOL BIOL TRANSL SCI*, **109** 75 (2012).

69 Groettrup, M., Kirk, C. J. & Basler, M., Proteasomes in immune cells: more than peptide producers? *NAT REV IMMUNOL* **10** 72 (2010).

70 Griffin, T. A. *et al.*, Immunoproteasome assembly: Cooperative incorporation of interferon gamma (IFN-gamma)-inducible subunits. *J EXP MED* **187** 97 (1998).

71 Arellano-Garcia, M. E., Misuno, K., Tran, S. D. & Hu, S., Interferon-gamma induces immunoproteasomes and the presentation of MHC I-associated peptides on human salivary gland cells. *PLOS ONE* **9** e102878 (2014).

72 Macagno, A. *et al.*, Dendritic cells up-regulate immunoproteasomes and the proteasome regulator PA28 during maturation. *EUR J IMMUNOL* **29** 4037 (1999).

73 Cui, Z., Hwang, S. M. & Gomes, A. V., Identification of the immunoproteasome as a novel regulator of skeletal muscle differentiation. *MOL CELL BIOL* **34** 96 (2014).

74 Xie, X. *et al.*, The immunoproteasome catalytic beta5i subunit regulates cardiac hypertrophy by targeting the autophagy protein ATG5 for degradation. *SCI ADV* **5** u495 (2019).

75 Keller, I. E. *et al.*, Regulation of immunoproteasome function in the lung. *SCI REP* **5** 10230 (2015).

76 Chen, S. *et al.*, Immunoproteasome dysfunction augments alternative polarization of alveolar macrophages. *CELL DEATH DIFFER* **23** 1026 (2016).

77 Eskandari, S. K., Seelen, M., Lin, G. & Azzi, J. R., The immunoproteasome: An old player with a novel and emerging role in alloimmunity. *AM J TRANSPLANT* **17** 3033 (2017).

78 Herrmann, J. *et al.*, The immunoproteasome - a new characteristic of symptomatic carotid artery plaques. *J AM COLL CARDIOL* **59S** E2054 (2012).

79 Seliger, B., Maeurer, M. J. & Ferrone, S., Antigen-processing machinery breakdown and tumor growth. *IMMUNOLOGY TODAY* **21** 455 (2000).

80 Ebstein, F., Kloetzel, P., Krueger, E. & Seifert, U., Emerging roles of immunoproteasomes beyond MHC class I antigen processing. *CELL MOL LIFE SCI* **69** 2543 (2012).

81 Chen, S. *et al.*, Immunoproteasome dysfunction augments alternative polarization of alveolar macrophages. *CELL DEATH DIFFER* **23** 1026 (2016).

82 Kremer, M. *et al.*, Reduced Immunoproteasome Formation and Accumulation of Immunoproteasomal Precursors in the Brains of Lymphocytic Choriomeningitis Virus-Infected Mice. *J IMMUNOL* **185** 5549 (2010).

83 Mundt, S., Basler, M., Buerger, S., Engler, H. & Groettrup, M., Inhibiting the immunoproteasome exacerbates the pathogenesis of systemic Candida albicans infection in mice. *SCI REP-UK* **6** (2016).

84 Chen, X. *et al.*, Inhibition of immunoproteasome reduces infarction volume and attenuates inflammatory reaction in a rat model of ischemic stroke. *CELL DEATH DIS* **6** (2015).

85 Sun, Y. *et al.*, miR-451 suppresses the NF-kappaB-mediated proinflammatory molecules expression through inhibiting LMP7 in diabetic nephropathy. *MOL CELL ENDOCRINOL* **433** 75 (2016).

86 Basler, M., Dajee, M., Moll, C., Groettrup, M. & Kirk, C. J., Prevention of Experimental Colitis by a Selective Inhibitor of the Immunoproteasome. *J IMMUNOL* **185** 634 (2010).

87 Basler, M., Kirk, C. J. & Groettrup, M., The immunoproteasome in antigen processing and other immunological functions. *CURR OPIN IMMUNOL* **25** 74 (2013).

88 Basler, M. *et al.*, Inhibition of the immunoproteasome ameliorates experimental autoimmune encephalomyelitis. *EMBO MOL MED* **6** 226 (2014).

89 Farini, A. *et al.*, Therapeutic Potential of Immunoproteasome Inhibition in Duchenne Muscular Dystrophy. *MOL THER* **24** 1898 (2016).

90 Yan, W. *et al.*, Knockout of immunoproteasome subunit beta2i ameliorates cardiac fibrosis and inflammation in DOCA/Salt hypertensive mice. *BIOCHEM BIOPHYS RES COMMUN* **490** 84 (2017).

91 Liong, S. *et al.*, The immunoproteasome inhibitor ONX-0914 regulates inflammation and expression of contraction associated proteins in myometrium. *EUR J IMMUNOL* **48** 1350 (2018).

92 Liao, J. *et al.*, Deficiency of LMP10 Attenuates Diet-Induced Atherosclerosis by Inhibiting Macrophage Polarization and Inflammation in Apolipoprotein E Deficient Mice. *Front Cell Dev Biol* **8** 592048 (2020).

93 Basler, M., Mundt, S. & Groettrup, M., The immunoproteasome subunit LMP7 is required in the murine thymus for filling up a hole in the Tcell repertoire. *EUR J IMMUNOL* **48** 419 (2018).

94 Basler, M. *et al.*, Co-inhibition of immunoproteasome subunits LMP2 and LMP7 is required to block autoimmunity. *EMBO REP* **19** (2018).

95 Mundt, S., Engelhardt, B., Kirk, C. J., Groettrup, M. & Basler, M., Inhibition and deficiency of the immunoproteasome subunit LMP7 attenuates LCMV-induced meningitis. *EUR J IMMUNOL* **46** 104 (2016).

96 Zaiss, D. M., Bekker, C. P., Grone, A., Lie, B. A. & Sijts, A. J., Proteasome immunosubunits protect against the development of CD8 T cell-mediated autoimmune diseases. *J IMMUNOL* **187** 2302 (2011).

97 Ichikawa, H. T. *et al.*, Beneficial effect of novel proteasome inhibitors in murine lupus via dual inhibition of type I interferon and autoantibody-secreting cells. *Arthritis Rheum* **64** 493 (2012).

98 Basler, M., Mundt, S., Bitzer, A., Schmidt, C. & Groettrup, M., The immunoproteasome: a novel drug target for autoimmune diseases. *CLIN EXP RHEUMATOL* **33** S74 (2015).

99 Koerner, J., Brunner, T. & Groettrup, M., Inhibition and deficiency of the immunoproteasome subunit LMP7 suppress the development and progression of colorectal carcinoma in mice. *ONCOTARGET* **8** 50873 (2017).

100 Vachharajani, N. *et al.*, Prevention of colitis-associated cancer by selective targeting of immunoproteasome subunit LMP7. *ONCOTARGET* **8** 50447 (2017).

101 Moallemian, R. *et al.*, Immunoproteasome inhibitor DPLG3 attenuates experimental colitis by restraining NF-kappaB activation. *BIOCHEM PHARMACOL* **177** 113964 (2020).

102 Schmidt, N. *et al.*, Targeting the proteasome: partial inhibition of the proteasome by bortezomib or deletion of the immunosubunit LMP7 attenuates experimental colitis. *GUT* **59** 896 (2010).

103 Althof, N. *et al.*, The immunoproteasome-specific inhibitor ONX 0914 reverses susceptibility to acute viral myocarditis. *EMBO MOL MED* **10** 200 (2018).

104 Liu, Q. *et al.*, Anti-viral immune response in the lung and thymus: Molecular characterization and expression analysis of immunoproteasome subunits LMP2, LMP7 and MECL-1 in pigs. *BIOCHEM BIOPHYS RES COMMUN* **502** 472 (2018).

105 Oh, I. S. *et al.*, Immunoproteasome induction is suppressed in hepatitis C virus-infected cells in a protein kinase R-dependent manner. *EXP MOL MED* **48** e270 (2016).

106 Moebius, J., van den Broek, M., Groettrup, M. & Basler, M., Immunoproteasomes are essential for survival and expansion of T cells in virus-infected mice. *EUR J IMMUNOL* **40** 3439 (2010).

107 Robek, M. D., Garcia, M. L., Boyd, B. S. & Chisari, F. V., Role of immunoproteasome catalytic subunits in the immune response to hepatitis B virus. *J VIROL* **81** 483 (2007).

108 Desterke, C., Turhan, A. G., Bennaceur-Griscelli, A. & Griscelli, F., HLA-dependent heterogeneity and macrophage immunoproteasome activation during lung COVID-19 disease. *J TRANSL MED* **19** 290 (2021).

109 Pang, K. C. *et al.*, Immunoproteasome subunit deficiencies impact differentially on two immunodominant influenza virus-specific CD8+ T cell responses. *J IMMUNOL* **177** 7680 (2006).

110 Gan, C. S., Lim, P. J., Razif, M. F., Yusof, R. & Othman, S., Subversion of immunoproteasome subunit expression in dengue virus serotype 2-infected HepG2 cells. *REV SOC BRAS MED TROP* **50** 99 (2017).

111 Santos, R. D. L. A. *et al.*, Structure of human immunoproteasome with a reversible and noncompetitive inhibitor that selectively inhibits activated lymphocytes. *NAT COMMUN* **8** (2017).

112 Karreci, E. S. *et al.*, Brief treatment with a highly selective immunoproteasome inhibitor

promotes long-term cardiac allograft acceptance in mice. *PROC NATL ACAD SCI USA* **113** E8425 (2016).
113 Xie, X. *et al.*, The immunoproteasome catalytic beta 5i subunit regulates cardiac hypertrophy by targeting the autophagy protein ATG5 for degradation. *SCI ADV* **5** (2019).
114 Wang, S. *et al.*, Ablation of Immunoproteasome beta 5i Subunit Suppresses Hypertensive Retinopathy by Blocking ATRAP Degradation in Mice. *MOL THER* **28** 279 (2020).
115 Liu, H. *et al.*, PR-957, a selective inhibitor of immunoproteasome subunit low-MW polypeptide 7, attenuates experimental autoimmune neuritis by suppressing T(h)17-cell differentiation and regulating cytokine production. *FASEB J* **31** 1756 (2017).
116 Liu, R. *et al.*, ONX-0914, a selective inhibitor of immunoproteasome, ameliorates experimental autoimmune myasthenia gravis by modulating humoral response. *J NEUROIMMUNOL* **311** 71 (2017).
117 Guo, Y. *et al.*, PR-957 mediates neuroprotection by inhibiting Th17 differentiation and modulating cytokine production in a mouse model of ischaemic stroke. *CLIN EXP IMMUNOL* **193** 194 (2018).
118 Muchamuel, T. *et al.*, A selective inhibitor of the immunoproteasome subunit LMP7 blocks cytokine production and attenuates progression of experimental arthritis. *NAT MED* **15** 712 (2009).
119 Nagayama, Y. *et al.*, Prophylactic and therapeutic efficacies of a selective inhibitor of the immunoproteasome for Hashimoto's thyroiditis, but not for Graves' hyperthyroidism, in mice. *CLIN EXP IMMUNOL* **168** 268 (2012).
120 Xiao, F. *et al.*, Proteasome inhibition suppresses Th17 cell generation and ameliorates autoimmune development in experimental Sjogren's syndrome. *CELL MOL IMMUNOL* **14** 924 (2017).
121 Huber, E. M. *et al.*, Immuno- and Constitutive Proteasome Crystal Structures Reveal Differences in Substrate and Inhibitor Specificity. *CELL* **148** 727 (2012).
122 Ettari, R. *et al.*, Immunoproteasome-selective and non-selective inhibitors: A promising approach for the treatment of multiple myeloma. *PHARMACOL THERAPEUT* **182** 176 (2018).
123 Ohsumi, Y., Molecular dissection of autophagy: Two ubiquitin-like systems. *NAT REV MOL CELL BIO* **2** 211 (2001).
124 Kraft, C. & Martens, S., Mechanisms and regulation of autophagosome formation. *CURR OPIN CELL BIOL* **24** 496 (2012).
125 Hollenstein, D. M. & Kraft, C., Autophagosomes are formed at a distinct cellular structure. *CURR OPIN CELL BIOL* **65** 50 (2020).
126 Xie, Z. & Klionsky, D. J., Autophagosome formation: core machinery and adaptations. *NAT CELL BIOL* **9** 1102 (2007).
127 Levine, B. & Kroemer, G., Biological Functions of Autophagy Genes: A Disease Perspective. *CELL* **176** 11 (2019).
128 Ma, X., McKeen, T., Zhang, J. & Ding, W. X., Role and Mechanisms of Mitophagy in Liver Diseases. *CELLS-BASEL* **9** (2020).
129 Dikic, I. & Elazar, Z., Mechanism and medical implications of mammalian autophagy. *NAT RRV MOL CELL BIOL* **19** 349 (2018).
130 Gatica, D., Lahiri, V. & Klionsky, D. J., Cargo recognition and degradation by selective autophagy. *NAT CELL BIOL* **20** 233 (2018).
131 Wen, X. & Klionsky, D. J., An overview of macroautophagy in yeast. *J MOL BIOL* **428** 1681 (2016).
132 Liu, X. M. *et al.*, ESCRTs Cooperate with a Selective Autophagy Receptor to Mediate Vacuolar Targeting of Soluble Cargos. *MOL CELL* **59** 1035 (2015).
133 Xu, Y. *et al.*, A Bacterial Effector Reveals the V-ATPase-ATG16L1 Axis that Initiates Xenophagy. *CELL* **178** 552 (2019).
134 Cuda, C. M., Pope, R. M. & Perlman, H., The inflammatory role of phagocyte apoptotic pathways in rheumatic diseases. *NAT REV RHEUMATOL* **12** 543 (2016).
135 Vomero, M. *et al.*, Autophagy and Rheumatoid Arthritis: Current Knowledges and Future Perspectives. *FRONT IMMUNOL* **9** (2018).
136 Karami, J. *et al.*, Role of autophagy in the pathogenesis of rheumatoid arthritis: Latest evidence and therapeutic approaches. *LIFE SCI* **254** (2020).
137 Retnakumar, S. V. & Muller, S., Pharmacological Autophagy Regulators as Therapeutic Agents for Inflammatory Bowel Diseases. *TRENDS MOL MED* **25** 516 (2019).
138 Jostins, L. *et al.*, Host-microbe interactions have shaped the genetic architecture of inflammatory bowel disease. *NATURE* **491** 119 (2012).
139 Harley, J. B. *et al.*, Genome-wide association scan in women with systemic lupus erythematosus identifies susceptibility variants in ITGAM, PXK, KIAA1542 and other loci. *NAT GENET* **40** 204 (2008).
140 Salem, M., Ammitzboell, M., Nys, K., Seidelin, J. B. & Nielsen, O. H., ATG16L1: A multifunctional susceptibility factor in Crohn disease. *AUTOPHAGY* **11** 585 (2015).

141 Cadwell, K., Patel, K. K., Komatsu, M., Virgin, H. W. & Stappenbeck, T. S., A common role for Atg16L1, Atg5 and Atg7 in small intestinal Paneth cells and Crohn disease. *AUTOPHAGY* **5** 250 (2009).

142 Ardali, R. *et al.*, Pathophysiological role of Atg5 in human ulcerative colitis. *INTESTINAL RESEARCH* **18** 421 (2020).

143 Lo Faro, M. L., Fox, B., Whatmore, J. L., Winyard, P. G. & Whiteman, M., Hydrogen sulfide and nitric oxide interactions in inflammation. *NITRIC OXIDE-BIOL CH* **41** 38 (2014).

144 Zhao, G. *et al.*, A Novel, Selective Inhibitor of Fibroblast Growth Factor Receptors That Shows a Potent Broad Spectrum of Antitumor Activity in Several Tumor Xenograft Models. *MOL CANCER THER* **10** 2200 (2011).

145 Michael, M. *et al.*, A Phase 1 Study of LY2874455, an Oral Selective pan-FGFR Inhibitor, in Patients with Advanced Cancer. *TARGET ONCOL* **12** 463 (2017).

146 He, C. *et al.*, Macrophages release plasma membrane-derived particles rich in accessible cholesterol. *PROC NATL ACAD SCI U S A* **115** E8499 (2018).

147 He, C. *et al.*, Cultured macrophages transfer surplus cholesterol into adjacent cells in the absence of serum or high-density lipoproteins. *PROC NATL ACAD SCI U S A* **117** 10476 (2020).

148 Zhang, Q., Lenardo, M. J. & Baltimore, D., 30 Years of NF-kappa B: A Blossoming of Relevance to Human Pathobiology. *CELL* **168** 37 (2017).

149 Catrysse, L. & van Loo, G., Inflammation and the Metabolic Syndrome: The Tissue-Specific Functions of NF-kappa B. *TRENDS CELL BIOL* **27** 417 (2017).

150 He, G. & Karin, M., NF-kappa B and STAT3 - key players in liver inflammation and cancer. *CELL RES* **21** 159 (2011).

151 Taniguchi, K. & Karin, M., NF-kappa B, inflammation, immunity and cancer: coming of age. *NAT REV IMMUNOL* **18** 309 (2018).

152 Blommaart, E. F., Krause, U., Schellens, J. P., Vreeling-Sindelarova, H. & Meijer, A. J., The phosphatidylinositol 3-kinase inhibitors wortmannin and LY294002 inhibit autophagy in isolated rat hepatocytes. *EUR J BIOCHEM* **243** 240 (1997).

153 Klionsky, D. J. *et al.*, Guidelines for the use and interpretation of assays for monitoring autophagy (4th edition). *AUTOPHAGY* **17** 1 (2021).

154 Klionsky DJ, A. K. A. A., Guidelines for the use and interpretation of assays for monitoring autophagy (3rd edition). *AUTOPHAGY* **12** 1 (2016).

155 Zhang, J. *et al.*, Targeting the Oncogenic FGF-FGFR Axis in Gastric Carcinogenesis. *CELLS-BASEL* **8** (2019).

156 Wheler, J. J. *et al.*, Presence of both alterations in FGFR/FGF and PI3K/AKT/mTOR confer improved outcomes for patients with metastatic breast cancer treated with PI3K/AKT/mTOR inhibitors. *ONCOSCIENCE* **3** 164 (2016).

157 Lin, X. *et al.*, FRS2 alpha is Essential for the Fibroblast Growth Factor to Regulate the mTOR Pathway and Autophagy in Mouse Embryonic Fibroblasts. *INT J BIOL SCI* **7** 1114 (2011).

158 Wang, Y. *et al.*, FGF-2 suppresses neuronal autophagy by regulating the PI3K/Akt pathway in subarachnoid hemorrhage. *BRAIN RES BULL* **173** 132 (2021).

159 Heras-Sandoval, D., Perez-Rojas, J. M., Hernandez-Damian, J. & Pedraza-Chaverri, J., The role of PI3K/AKT/mTOR pathway in the modulation of autophagy and the clearance of protein aggregates in neurodegeneration. *CELL SIGNAL* **26** 2694 (2014).

160 Li, N. *et al.*, Sodium butyrate alleviates LPS-induced acute lung injury in mice via inhibiting HMGB1 release. *INT IMMUNOPHARMACOL* **56** 242 (2018).

161 He, R. *et al.*, Itaconate inhibits ferroptosis of macrophage via Nrf2 pathways against sepsis-induced acute lung injury. *CELL DRATH DISCOV* **8** 43 (2022).

162 Bar-Yosef, T., Damri, O. & Agam, G., Dual Role of Autophagy in Diseases of the Central Nervous System. *FRONT CELL NEUROSCI* **13** (2019).

163 Rahman, M. A. & Rhim, H., Therapeutic implication of autophagy in neurodegenerative diseases. *BMB REP* **50** 345 (2017).

164 Lee, J. *et al.*, TLR4 (toll-like receptor 4) activation suppresses autophagy through inhibition of FOXO3 and impairs phagocytic capacity of microglia. *AUTOPHAGY* **15** 753 (2019).

165 He, Y. *et al.*, p38 MAPK inhibits autophagy and promotes microglial inflammatory responses by phosphorylating ULK1. *J CELL BIOL* **217** 315 (2018).

166 Wang, H., Guo, Y., Qiao, Y., Zhang, J. & Jiang, P., Nobiletin Ameliorates NLRP3 Inflammasome-Mediated Inflammation Through Promoting Autophagy via the AMPK Pathway. *MOL NEUROBIOL* **57** 5056 (2020).

167 Lyman, M., Lloyd, D. G., Ji, X., Vizcaychipi, M. P. & Ma, D., Neuroinflammation: The role and consequences. *NEUROSCI RES* **79** 1 (2014).

168 Lu, Q. *et al.*, Homeostatic Control of Innate Lung Inflammation by Vici Syndrome Gene Epg5 and Additional Autophagy Genes Promotes Influenza Pathogenesis. *CELL HOST MICROBE* **19** 102 (2016).

169 Park, S. *et al.*, Autophagy Genes Enhance Murine Gammaherpes virus 68 Reactivation from Latency by Preventing Virus-Induced Systemic Inflammation. *CELL HOST MICROBE* **19** 91 (2016).

170 Yu, L., Chen, Y. & Tooze, S. A., Autophagy pathway: Cellular and molecular mechanisms. *AUTOPHAGY* **14** 207 (2018).

171 Nishimura, T. & Tooze, S. A., Emerging roles of ATG proteins and membrane lipids in autophagosome formation. *CELL DISCOV* **6** (2020).

172 Yim, W. W. & Mizushima, N., Lysosome biology in autophagy. *CELL DISCOV* **6** (2020).

173 Johansen, T. & Lamark, T., Selective Autophagy: ATG8 Family Proteins, LIR Motifs and Cargo Receptors. *J MOL BIOL* **432** 80 (2020).

174 Yao, R., Ren, C., Xia, Z. & Yao, Y., Organelle-specific autophagy in inflammatory diseases: a potential therapeutic target underlying the quality control of multiple organelles. *AUTOPHAGY.* **17** 2 (2021.)

175 Kirkin, V. & Rogov, V. V., A Diversity of Selective Autophagy Receptors Determines the Specificity of the Autophagy Pathway. *MOL CELL* **76** 268 (2019).

Fig. 1

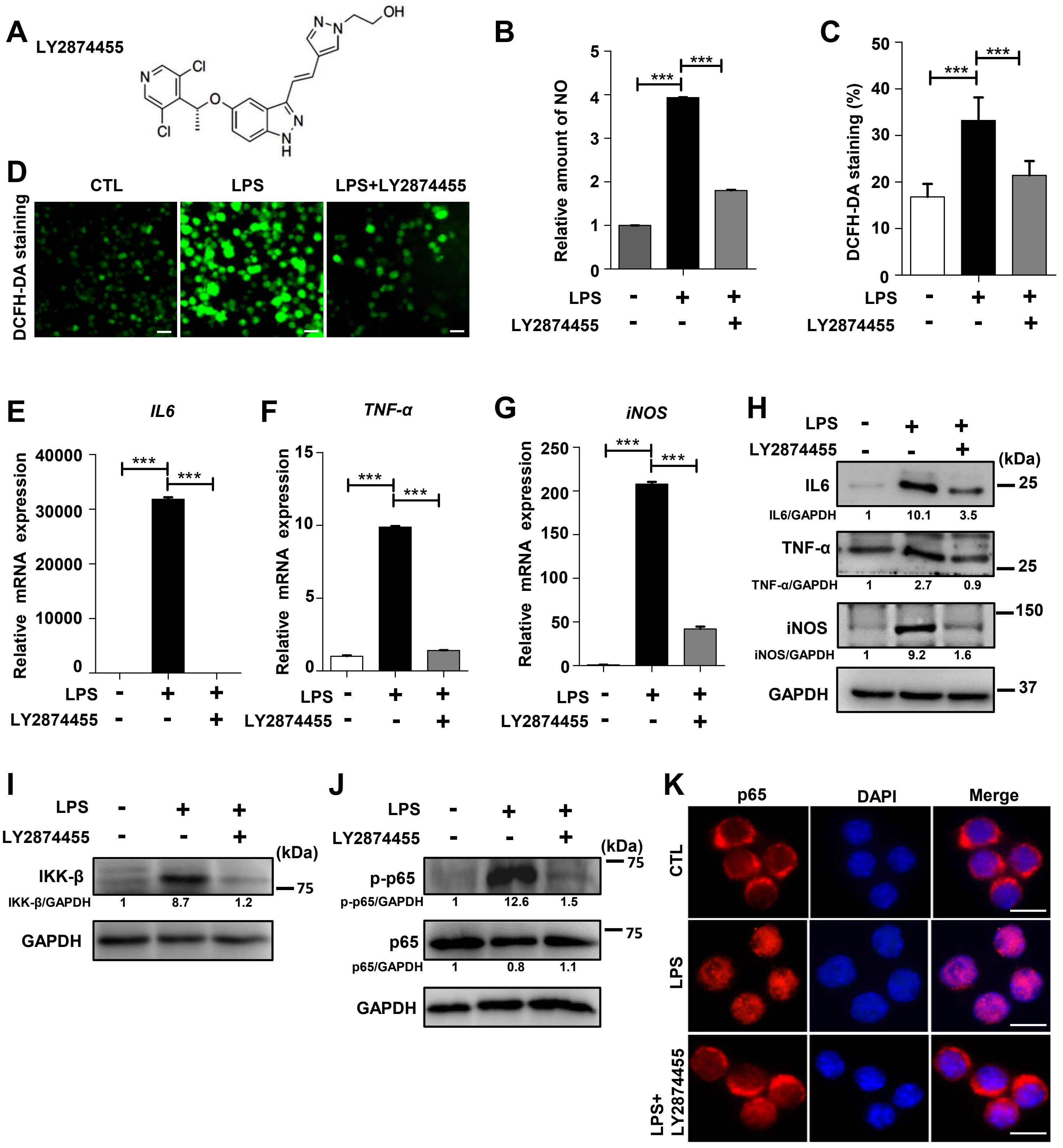
A
LY2874455
B
C
D
CTL
LPS
LPS+LY2874455
DCFH-DA staining
Relative amount of NO
DCFH-DA staining (%)
LPS
LY2874455
E
IL6
F
TNF-α
G
iNOS
Relative mRNA expression
H
IL6
IL6/GAPDH 1 10.1 3.5
TNF-α
TNF-α/GAPDH 1 2.7 0.9
iNOS
iNOS/GAPDH 1 9.2 1.6
GAPDH
(kDa)
I
IKK-β
IKK-β/GAPDH 1 8.7 1.2
J
p-p65
p-p65/GAPDH 1 12.6 1.5
p65
p65/GAPDH 1 0.8 1.1
K
p65
DAPI
Merge
CTL
LPS
LPS+ LY2874455

Fig. 1 LY2874455 attenuated the inflammatory reactions in LPS-stimulated macrophages. (A) The chemical structure of LY2874455. **(B-K)** Macrophage RAW264.7 cells were stimulated with LPS (20ng/ml) for 24h together with or without LY2874455 (2μM). **(B)** LY2874455 abolished the release of nitric oxide (NO) from LPS-stimulated RAW264.7 cells. NO concentration in culture supernatant was measured and shown as fold change compared to the control group. Similar results were obtained from three independent replicated experiments. $^{**}P<0.01$, $^{***}P<0.001$. **(C)** LY2874455 abolished the generation of reactive oxygen species (ROS) in LPS-stimulated RAW264.7 cells. After treatment with LPS or/and LY2874455, RAW264.7 cells were washed and labeled with DCFH-DA, a ROS probe. Quantitative measurement of cellular DCFH-DA staining was performed by flow cytometry. Similar results were obtained from three independent experiments. **(D)** A set of representative images showing fluorescent staining of ROS by DCFH-DA probe were shown. Scale bars: 10μm. **(E-G)** LY2874455 suppressed the expression of proinflammatory cytokines and NO synthetase in macrophages stimulated with LPS. The expression of proinflammatory cytokine genes (IL-6, TNF-α) and NO synthetase (iNOS) was checked by quantitative real-time PCR (qRT-PCR). **(H-J)** Total proteins were extracted from RAW264.7 cells and used for western blot analysis of protein levels of IL6, TNF-α, iNOS, IKK-β, p65 and phosphorylated p65 (p-p65). For the similar experiments shown by immunoblots, three independent experiments were conducted and representative images were shown. The blots were quantified with densitometric values and statistical significance was analyzed. **(K)** LY2874455 suppressed the nucleus translocation of phosphorylated p65 in macrophages stimulated with LPS. RAW264.7 cells were stimulated with LPS (20ng/ml) for 2 h with or without LY2874455 (2μM). Representative images of double immunostaining for p65 and nucleus (DAPI) were shown. Scale bars: 10μm.

Supplementary Fig. S1

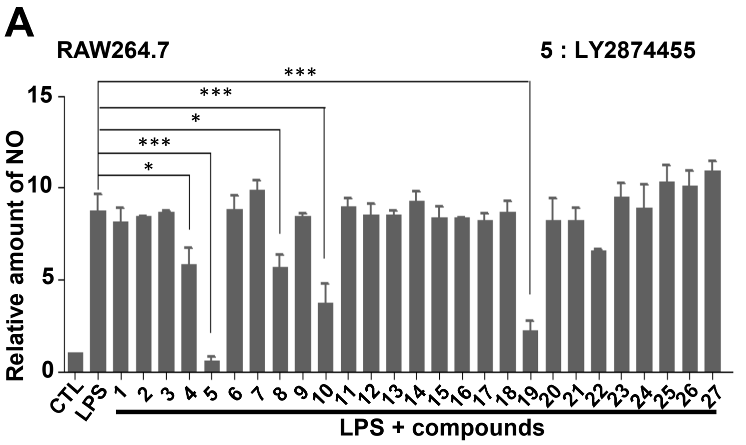

B

1: FRAX486	10: Cetylpyridinium (chloride monohydrate)	19: Fenticonazole (Nitrate)
2: Chlorindione	11: Voriconazole	20: Anisindione
3: Luliconazole	12: Hexylresorcinol	21: OSI-930
4: SKLB1002	13: Nitroxoline	22: Parthenolide
5: LY2874455	14: TCS-PIM-1-4a	23: Colistin (sulfate)
6: Salmeterol (xinafoate)	15: Chlorhexidine	24: Bifonazole
7: Sulconazole (nitrate)	16: Carprofen	25: A 419259 (trihydrochloride)
8: Domiphen (bromide)	17: Caspofungin (Acetate)	26: Chlorquinaldol
9: IPA-3	18: Posaconazole	27: 1-Naphthyl PP1

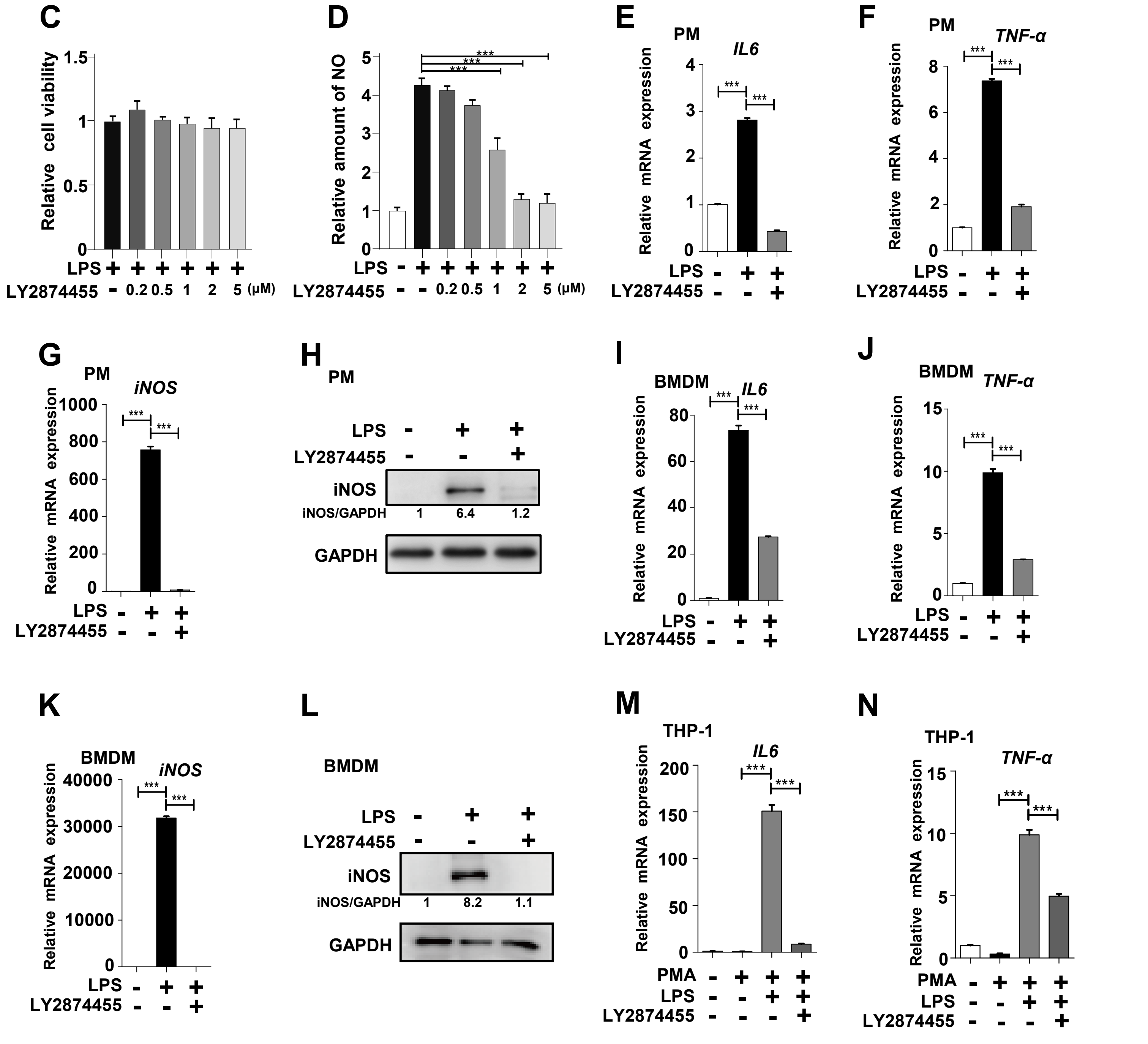

Supplementary Fig. S1 LY2874455 suppressed inflammation in LPS-stimulated macrophages. (A) RAW264.7 cells were incubated with 27 chemicals and stimulated with LPS (20ng/ml) for 24h. NO concentration in culture supernatant was measured and shown as fold change. **(B)** The names of the 27 chemicals. **(C-D)** Different concentrations of LY2874455 were applied to LPS (20ng/ml)-treated RAW264.7 cells and cell viabilities and levels of NO were measured. **(E-G)** The expression of proinflammatory genes (IL-6, TNF-α) and iNOS was checked by qRT-PCR in peritoneal macrophages (PM) treated with LY2874455 (2μM) and LPS (20ng/ml) for 24h. **(H)** Protein levels of iNOS and GAPDH were detected by western blot in PM. The blot was quantified with densitometric values and statistical significance was analyzed. **(I-K)** The expression of proinflammatory genes (IL-6, TNF-α) and iNOS was checked by qRT-PCR in bone marrow derived macrophages (BMDM) treated with LY2874455 (2μM) and LPS (20ng/ml) for 24h. **(L)** Protein levels of iNOS and GAPDH were detected by western blot in BMDM. The blot was quantified with densitometric values and statistical significance was analyzed. **(M-N)** The expression of proinflammatory genes (IL-6 and TNF-α) was checked by qRT-PCR in human THP-1 monocytes treated with LY2874455 (2μM) and LPS (20ng/ml) for 24h. Phorbol12-myristate13-acetate (PMA) was used to induce THP-1 monocyte to macrophages.

Fig. 2

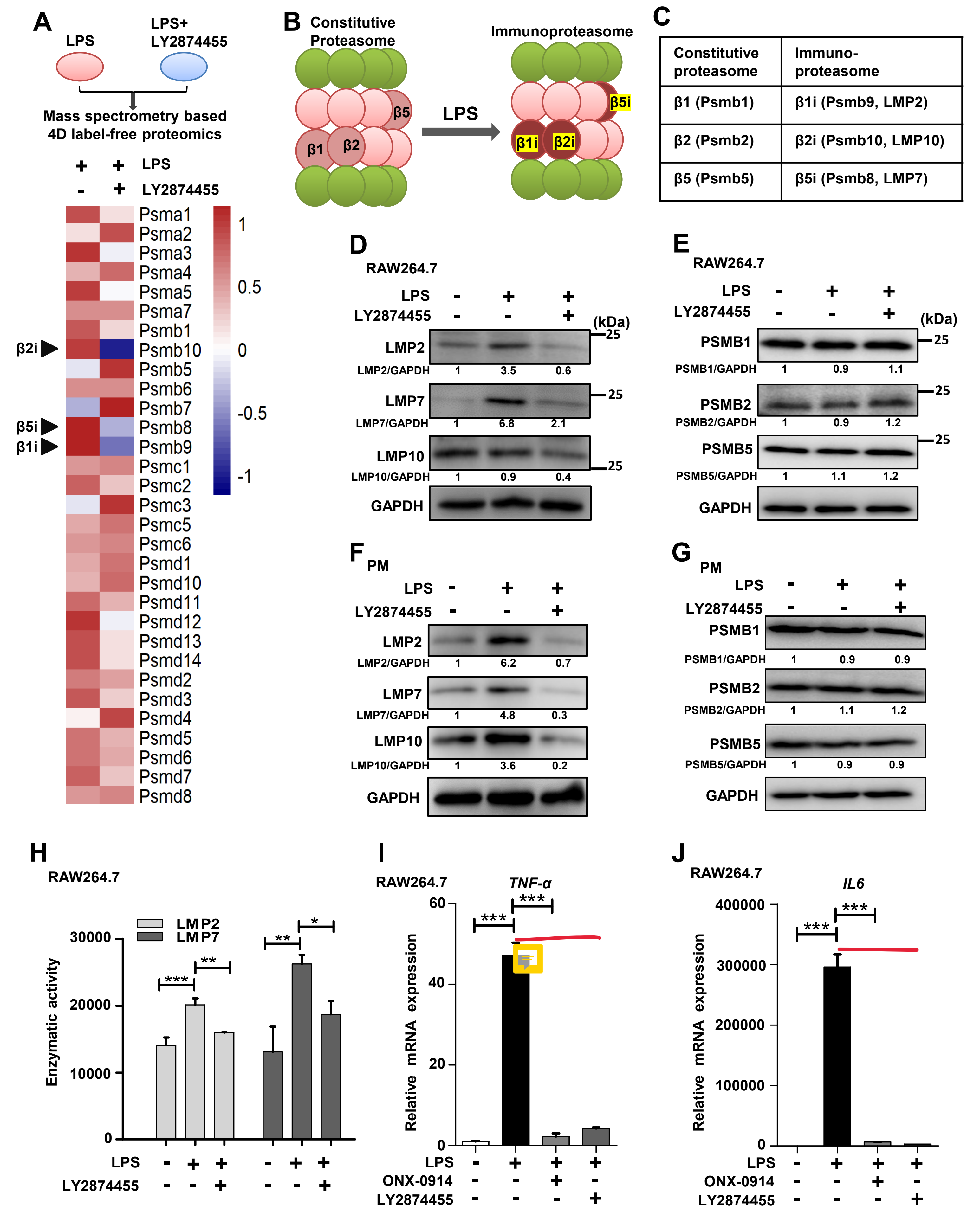
A
LPS
LPS+ LY2874455
Mass spectrometry based 4D label-free proteomics
LPS
LY2874455
Psma1
Psma2
Psma3
Psma4
Psma5
Psma7
Psmb1
Psmb10
Psmb5
Psmb6
Psmb7
Psmb8
Psmb9
Psmc1
Psmc2
Psmc3
Psmc5
Psmc6
Psmd1
Psmd10
Psmd11
Psmd12
Psmd13
Psmd14
Psmd2
Psmd3
Psmd4
Psmd5
Psmd6
Psmd7
Psmd8
β2i
β5i
β1i
1
0.5
0
-0.5
-1
B
Constitutive Proteasome
Immunoproteasome
LPS
β1
β2
β5
β1i
β2i
β5i
C
Constitutive proteasome | Immuno-proteasome
β1 (Psmb1) | β1i (Psmb9, LMP2)
β2 (Psmb2) | β2i (Psmb10, LMP10)
β5 (Psmb5) | β5i (Psmb8, LMP7)
D
RAW264.7
LPS - + +
LY2874455 - - +
(kDa)
LMP2
LMP2/GAPDH 1 3.5 0.6
LMP7
LMP7/GAPDH 1 6.8 2.1
LMP10
LMP10/GAPDH 1 0.9 0.4
GAPDH
25
E
RAW264.7
LPS - + +
LY2874455 - - +
(kDa)
PSMB1
PSMB1/GAPDH 1 0.9 1.1
PSMB2
PSMB2/GAPDH 1 0.9 1.2
PSMB5
PSMB5/GAPDH 1 1.1 1.2
GAPDH
25
F
PM
LPS - + +
LY2874455 - - +
LMP2
LMP2/GAPDH 1 6.2 0.7
LMP7
LMP7/GAPDH 1 4.8 0.3
LMP10
LMP10/GAPDH 1 3.6 0.2
GAPDH
G
PM
LPS - + +
LY2874455 - - +
PSMB1
PSMB1/GAPDH 1 0.9 0.9
PSMB2
PSMB2/GAPDH 1 1.1 1.2
PSMB5
PSMB5/GAPDH 1 0.9 0.9
GAPDH
H
RAW264.7
LMP2
LMP7
Enzymatic activity
30000
20000
10000
0
***
**
**
*
LPS - + + - + +
LY2874455 - - + - - +
I
RAW264.7
TNF-α
Relative mRNA expression
60
40
20
0
***
***
LPS - + + +
ONX-0914 - - + -
LY2874455 - - - +
J
RAW264.7
IL6
Relative mRNA expression
400000
300000
200000
100000
0
***
***
LPS - + + +
ONX-0914 - - + -
LY2874455 - - - +

Fig. 2 LY2874455 reduced the protein levels of immunoproteasome specific subunits in LPS-stimulated macrophages. **(A)** Above, method diagram of mass spectrometry-based proteomics analysis of RAW264.7 cells treated with LPS (20ng/ml) or/and LY2874455. The original mass spectrometry proteomics data have been deposited to the ProteomeXchange Consortium via the PRIDE partner repository with the dataset identifier PXD038747. Bottom, heat map of protein levels of proteasome subunits. The protein levels of immunoproteasome specific subunits (arrow pointed) were specifically reduced by LY2874455 treatment. **(B-C)** The diagram and names of the specific subunit of 20s constitutive proteasome and immunoproteasome. **(D)** LY2874455 reduced the protein levels of immunoproteasome specific subunits. Protein levels of immunoproteasome subunits LMP2, LMP7, LMP10 and control GAPDH were analyzed in RAW264.7 cells treated with LY2874455 (2μM) and LPS (20ng/ml) for 24 h. The blots were quantified with densitometric values and statistical significance was analyzed. **(E)** LY2874455 had no effect on the protein levels of constitutive proteasome specific subunits. Protein levels of constitutive proteasome subunits PSMB1, PSMB2, PSMB5 and control GAPDH were analyzed as in (D). The blots were quantified with densitometric values and statistical significance was analyzed. **(F)** Peritoneal macrophages (PM) from C57BL/6J mice were obtained and treated with LPS (20ng/ml) and/or LY2874455 (2μM) for 24h. The total proteins were extracted to detect the protein levels of immunoproteasome subunits LMP2, LMP7, LMP10 and GAPDH. The blots were quantified with densitometric values and statistical significance was analyzed. **(G)** Protein levels of constitutive proteasome subunits PSMB1, PSMB2, PSMB5 and GAPDH were detected. The blots were quantified with densitometric values and statistical significance was analyzed. **(H)** The enzymatic activities of immunoproteasome subunits LMP2 and LMP7 were measured in RAW264.7 cells. *: $P<0.05$, **: $P<0.01$, ***: $P<0.001$. **(I-J)** LY2874455 could not cause further repression on inflammation in macrophages with inhibited immunoproteasome. The specific inhibitor of immunoproteasome, ONX-0914, suppressed expression of inflammatory cytokines in macrophages stimulated with LPS. Additional treatment with LY2874455 could not cause further inflammation-repression. The expression of inflammatory cytokine genes (TNF-α and IL-6) was checked by qRT-PCR in RAW264.7 cells treated with LPS, the immunoproteasome inhibitor ONX-0914(1μM) and LY2874455 (2μM) for 24h. ***: $P<0.001$. NS: no statistical difference.

Supplementary Fig. S2

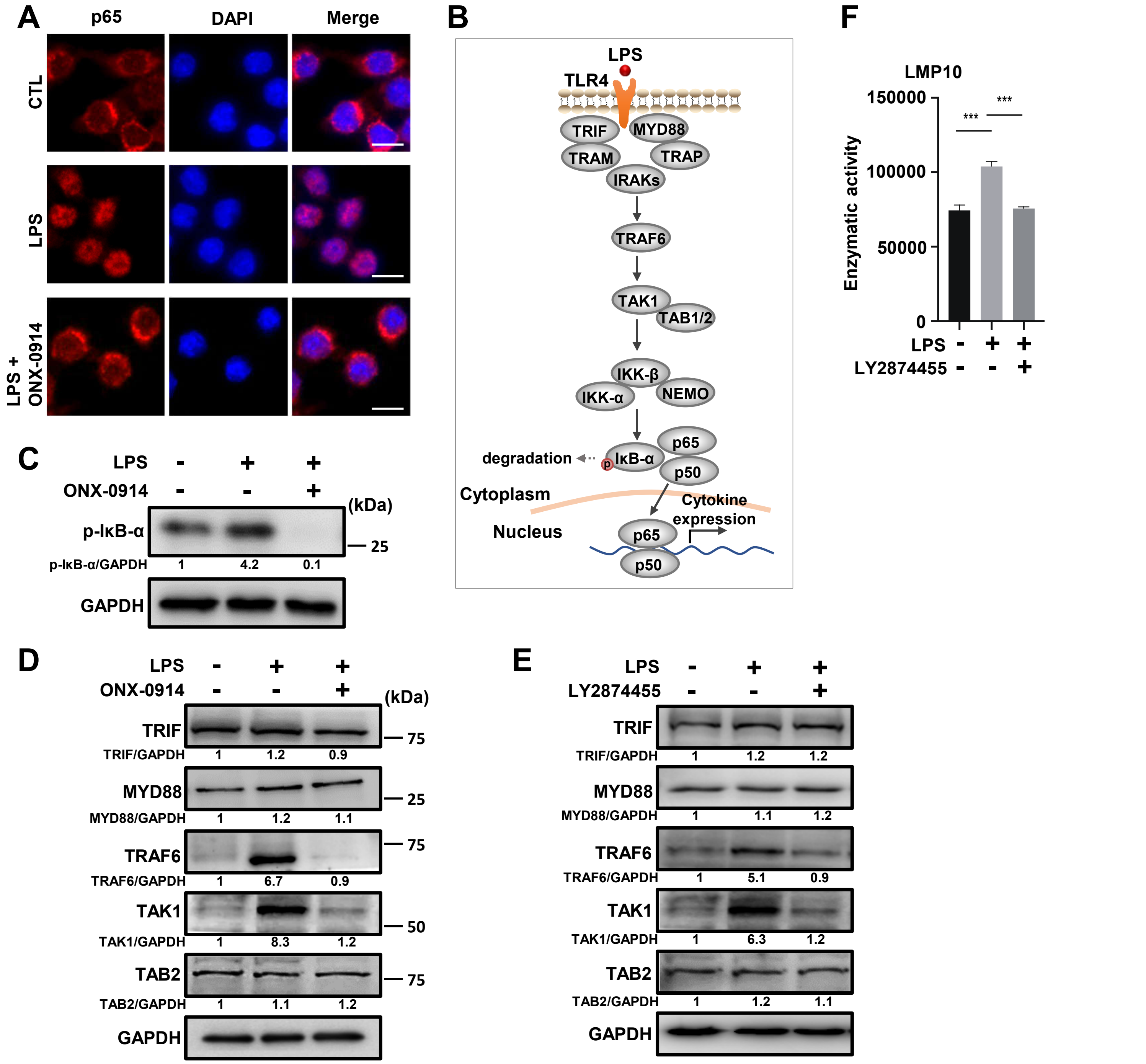

Supplementary Fig. S2 Immunoproteasome functions in LPS-induced inflammation ~~at~~ upstream of NF-κB factors. (A) Immunoproteasome inhibitor ONX-0914 suppressed the nucleus translocation of NF-κB factor p65 in macrophages stimulated with LPS. RAW264.7 cells were stimulated with LPS (20ng/ml) for 2 h with or without ONX-0914 (1μM). Representative images of were shown. Scale bars: 10μm. **(B)** Schematic diagram of NF-κB inflammation signaling pathway. **(C)** Total proteins were extracted from RAW264.7 macrophage cells treated with LPS (20ng/ml) together with ONX-0914 (1μM) and used for western blot analysis of protein levels of phosphorylated IκB-α (p-IκB-α). The blot was quantified with densitometric values and statistical significance was analyzed. **(D-E)** Total proteins were extracted from RAW264.7 macrophage cells treated with LPS (20ng/ml) together with ONX-0914 (1μM) or LY2874455 (2μM) and used for western blot analysis of protein levels of indicated factors function at upstream of NF-κB signaling pathway. The blots were quantified with densitometric values and statistical significance was analyzed. **(F)** The enzymatic activities of immunoproteasome subunits LMP10 were measured in RAW264.7 cells. ***: P<0.001.

Fig. 3

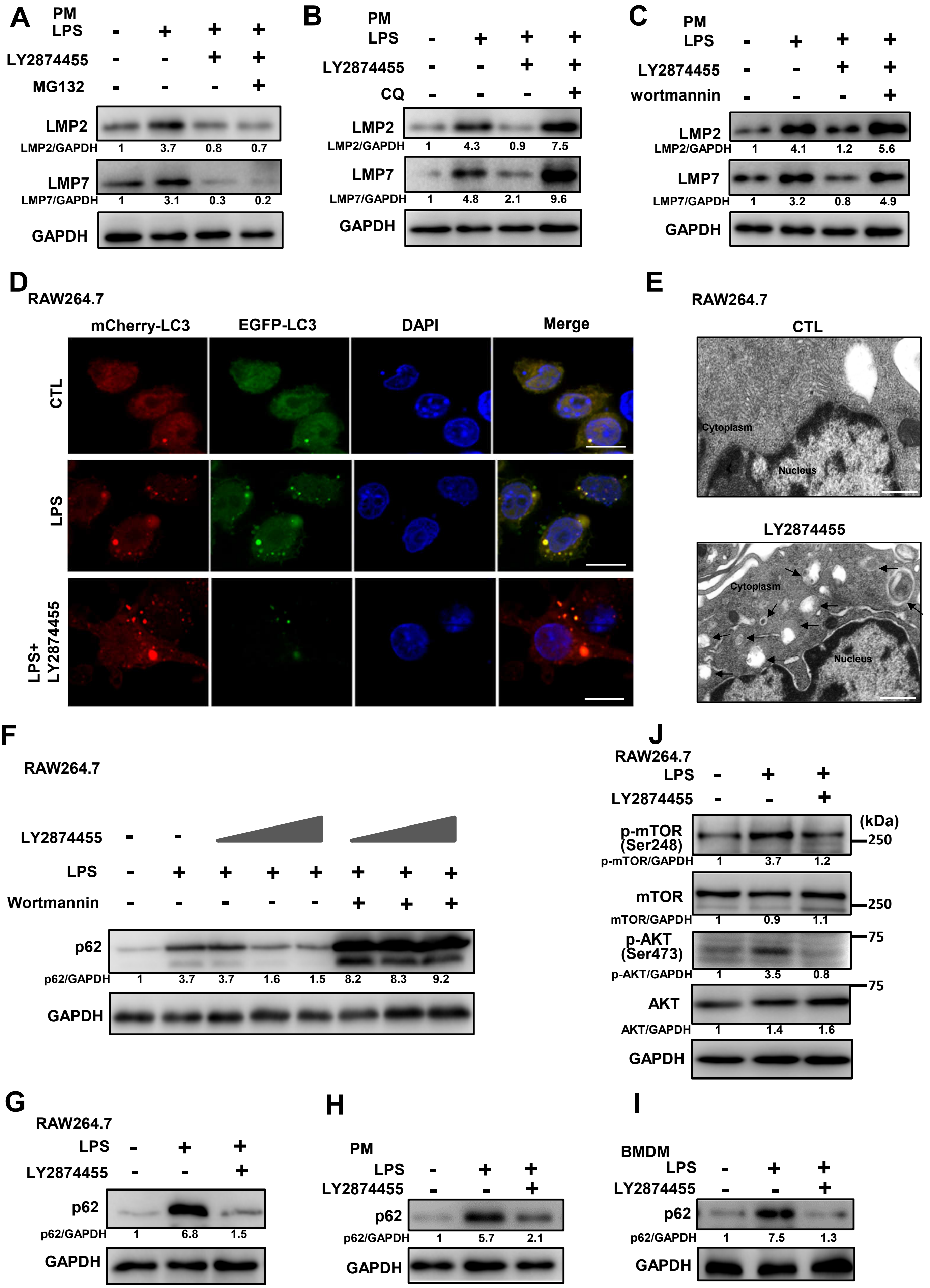

A
PM
LPS - + + +
LY2874455 - - + +
MG132 - - - +
LMP2
LMP2/GAPDH 1 3.7 0.8 0.7
LMP7
LMP7/GAPDH 1 3.1 0.3 0.2
GAPDH
B
PM
LPS - + + +
LY2874455 - - + +
CQ - - - +
LMP2
LMP2/GAPDH 1 4.3 0.9 7.5
LMP7
LMP7/GAPDH 1 4.8 2.1 9.6
GAPDH
C
PM
LPS - + + +
LY2874455 - - + +
wortmannin - - - +
LMP2
LMP2/GAPDH 1 4.1 1.2 5.6
LMP7
LMP7/GAPDH 1 3.2 0.8 4.9
GAPDH
D
RAW264.7
mCherry-LC3
EGFP-LC3
DAPI
Merge
CTL
LPS
LPS+ LY2874455
E
RAW264.7
CTL
Cytoplasm
Nucleus
LY2874455
Cytoplasm
Nucleus
F
RAW264.7
LY2874455 - -
LPS - + + + + + + +
Wortmannin - - - - - + + +
p62
p62/GAPDH 1 3.7 3.7 1.6 1.5 8.2 8.3 9.2
GAPDH
J
RAW264.7
LPS - + +
LY2874455 - - +
(kDa)
p-mTOR (Ser248)
250
p-mTOR/GAPDH 1 3.7 1.2
mTOR
250
mTOR/GAPDH 1 0.9 1.1
p-AKT (Ser473)
75
p-AKT/GAPDH 1 3.5 0.8
75
AKT
AKT/GAPDH 1 1.4 1.6
GAPDH
G
RAW264.7
LPS - + +
LY2874455 - - +
p62
p62/GAPDH 1 6.8 1.5
GAPDH
H
PM
LPS - + +
LY2874455 - - +
p62
p62/GAPDH 1 5.7 2.1
GAPDH
I
BMDM
LPS - + +
LY2874455 - - +
p62
p62/GAPDH 1 7.5 1.3
GAPDH

Fig. 3 LY2874455 activated the autophagy process. (A) Proteasome inhibitor MG132 cannot block the reduction of immunoproteasome subunits induced by LY2874455. Protein levels of LMP2, LMP7 and GAPDH were analyzed in RAW264.7 cells treated with LPS (20ng/ml), LY2874455 (2μM) and MG132 (5μM). The blots were quantified with densitometric values and statistical significance was analyzed. **(B-C)** Autophagy inhibitors blocked the reduction of immunoproteasome subunits induced by LY2874455. Protein levels of LMP2, LMP7 and GAPDH were analyzed in RAW264.7 cells treated with LPS (20ng/ml), LY2874455 (2μM) and autophagy inhibitors chloroquine (CQ, 20μM) or wortmannin (100nM). The blots were quantified with densitometric values and statistical significance was analyzed. **(D)** LY2874455 enhanced autophagy flux. In RAW264.7 cells transfect with mCherry-EGFP-LC3 for 24h and then stimulated with LPS (20ng/ml) or/and LY2874455 for another 24h, and the puncta of LC3 in cells was monitored after fixation under a confocal fluorescent microscope. Higher level of mCherry-LC3 signal and lower level of EGFP-LC3 signal (showing red in merge) indicated the stimulated autophagy flux, while the similar signal levels of mCherry-LC3 and GFP-L3 (showing yellow in merge) indicated the blocked autophagy flux. Representative images from three replicated experiments were shown. Scale bars: 10μm. **(E)** LY2874455 promoted the formation of autophagosomes. RAW264.7 cells treated with control or LY2874455 (2μM) for 24h were analyzed by transmission electron microscopy. Autophagosomes were pointed by arrows and representative images from three replicated experiments were shown. Scale bars: 1 μm. **(F)** LY2874455 promoted the autophagic degradation of substrate receptor p62. RAW264.7 cells were treated with LPS (20ng/ml) and LY2874455 (0.5, 2, 4μM) with or without Wortmannin (100nM) for 24h and protein levels of p62 and GAPDH were analyzed by western blot. The blots were quantified with densitometric values and statistical significance was analyzed. **(G)** Protein levels of p62 and GAPDH were detected by western blot in RAW264.7 cells treated with LY2874455 (2μM) and LPS (20ng/ml) and for 24h. The blot was quantified with densitometric values and statistical significance was analyzed. **(H)** Protein levels of p62 and GAPDH were detected by western blot in peritoneal macrophages (PM) from C57BL/6J mice treated as in (G). The blot was quantified with densitometric values and statistical significance was analyzed. **(I)** Protein levels of p62 and GAPDH were detected by western blot in bone marrow derived macrophages (BMDM) from C57BL/6J mice treated as in (G). The blot was quantified with densitometric values and statistical significance was analyzed. **(J)** LY2874455 inhibited the AKT-mTOR signaling. Protein levels of p-mTOR (Ser248), mTOR, p-AKT(Ser473), AKT and GAPDH were detected by western blot in RAW264.7 cells treated as in (G). The blots were quantified with densitometric values and statistical significance was analyzed.

Supplementary Fig. S3

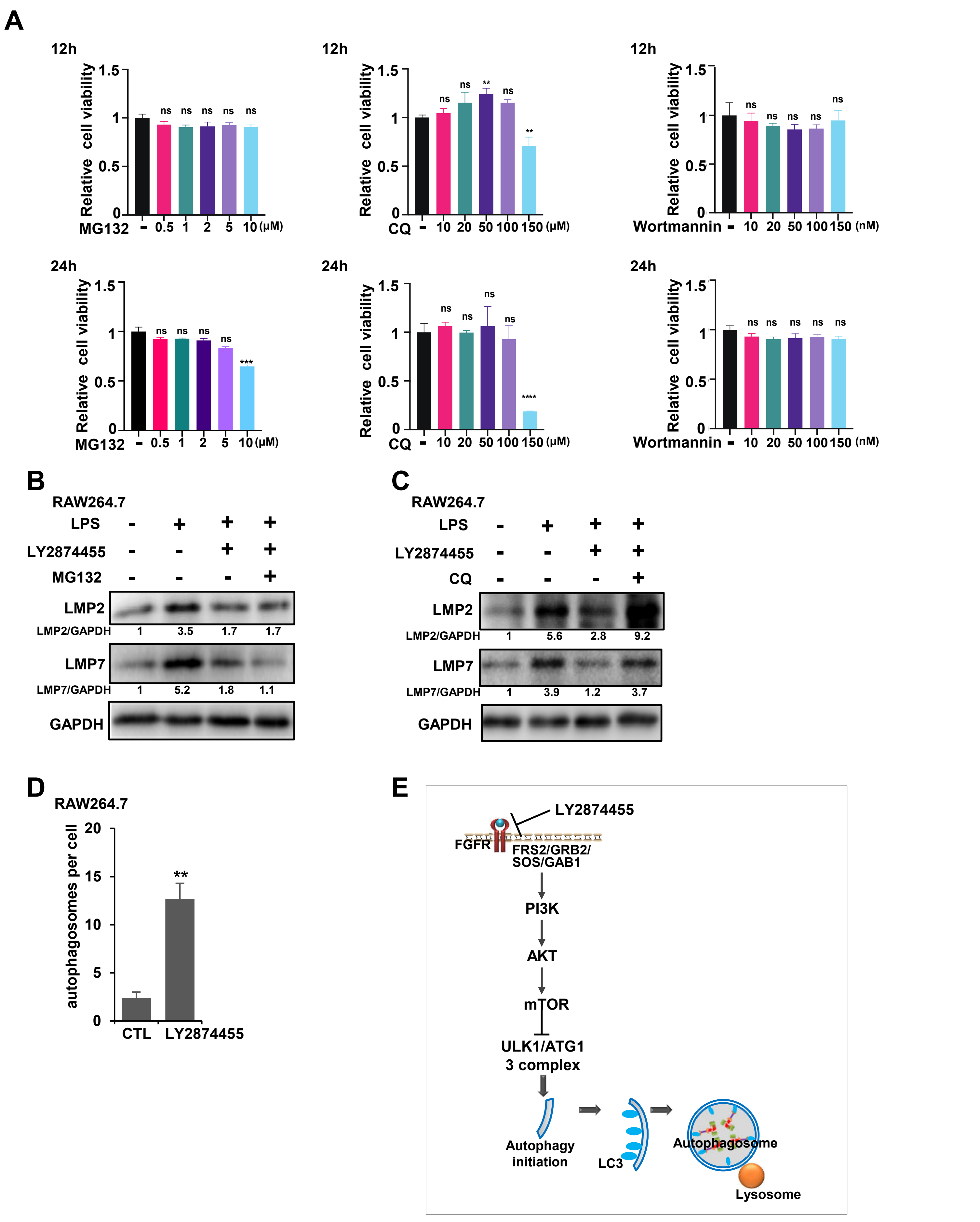

Supplementary Fig. S3 LY2874455 induced autophagy in macrophages. (A) Inhibitors MG132, chloroquine or wortmannin were used at different concentrations to analyze their effects on macrophage viabilities in the dose- and time-dependent cell death assays. **(B)** Inhibitor of constitutive proteasome, MG132, could not block the reduction of immunoproteasome subunits induced by LY2874455. RAW264.7 cells were treated with LPS (20ng/ml), LY2874455 (2μM) and proteasome inhibitor MG132 (5μM). Protein levels of LMP2, LMP7 and GAPDH were detected by western blot. The blots were quantified with densitometric values and statistical significance was analyzed. **(C)** Autophagy inhibitor wortmannin blocked the reduction of immunoproteasome subunits induced by LY2874455. Protein levels of LMP2, LMP7 and GAPDH were analyzed in RAW264.7 cells treated with LPS (20ng/ml), LY2874455 (2μM) and autophagy inhibitor Wortmannin (100nM). The blots were quantified with densitometric values and statistical significance was analyzed. **(D)** Quantification of autophagosomes in macrophages treated with LY2874455 for results in Fig.3E. **(E)** Model scheme of LY2874455 effect on activation of autophagy. FGFRs are transmembrane RTKs and activated by FGF to form dimers, leading to the activation of downstream FRS2 complex and subsequent activation of PI3K/AKT/mTOR signaling pathway. Activated mTOR then phosphorylates and inhibits autophagy initiating ULK1 kinase complex, leading to suppression of autophagy. LY2874455 suppresses the FGFR activities and the downstream PI3K/AKT/mTOR signaling pathway, leading to the release and activation of autophagy.

Fig. 4

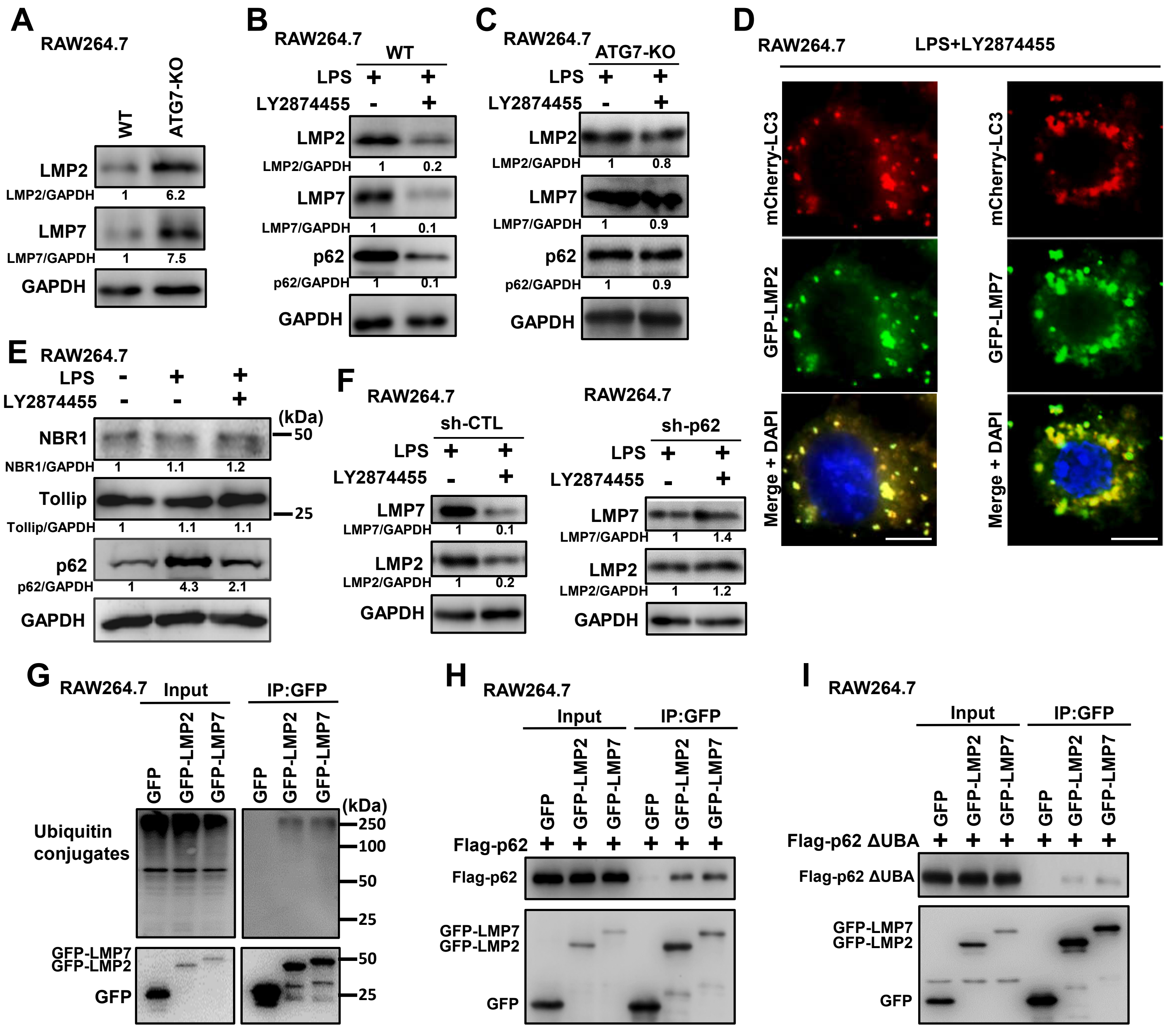

Fig. 4 LY2874455 promoted the degradation of immunoproteasomes through autophagy and selective receptor p62. (A) Autophagy deficiency caused increased levels of immunoproteasome subunits. Protein levels of LMP2, LMP7and GAPDH were detected by western blot in wild type (WT) or ATG7 knock out (ATG7-KO, through CRISPR-CAS9 method) RAW264.7 cells. The blots were quantified with densitometric values and statistical significance was analyzed. **(B-C)** LY2874455 could not reduce the protein levels of immunoproteasome subunits in autophagy deficient macrophages. WT or ATG7-KO RAW264.7 cells were treated with LPS (20ng/ml) alone or together with LY2874455 (2μM) for 24h. Protein levels of LMP2, LMP7, p62 and GAPDH were analyzed by western blot. The blots were quantified with densitometric values and statistical significance was analyzed.

Fig. 4 LY2874455 promoted the degradation of immunoproteasomes through autophagy and selective receptor p62.

(D) Immunoproteasome subunits co-localized with autophagosome marker LC3. RAW264.7 cells expressing mCherry-tagged LC3 and GFP-tagged LMP2 or LMP7 were stimulated with LPS (20ng/ml) and LY2874455 (2μM) for 24h. The co-localization of GFP-LMP2/GFP-LMP7 with mCherry-LC3 was observed by confocal fluorescent microscope. Scale bars: 10μm. **(E)** LY2874455 reduced the protein levels of selective autophagy receptor p62. RAW264.7 cells were treated with LPS (20ng/ml) and LY2874455 (2μM) for 24h, and then the protein levels of NBR1, Tollip, p62 and GAPDH were detected by western blot. The blots were quantified with densitometric values and statistical significance was analyzed. **(F)** Knock down of p62 caused increase of protein levels of immunoproteasome subunits. The protein levels of LMP2, LMP7 and GAPDH were detected by western blot in control shRNA or p62-targeting shRNA transfected RAW264.7 cells treated with LPS (20ng/ml) and LY2874455 (2μM) for 24h. The blots were quantified with densitometric values and statistical significance was analyzed. **(G)** Immunoproteasome subunits were ubiquitinated. RAW264.7 cells expressing GFP-LMP2 or GFP-LMP7 were treated with LY2874455 (2μM) for 24h. Then equal amounts of protein lysates were subject to immunoprecipitation with anti-GFP affinity beads and analyzed by western blot with anti-GFP and anti-ubiquitin antibodies. **(H-I)** p62 interacted with immunoproteasome subunits through its ubiquitin-binding UBA domain. RAW264.7 cells expressing GFP-LMP2, GFP-LMP7 and Flag-tagged full-length p62 or truncated p62 (ubiquitin-binding domain UBA deleted) were subject to immunoprecipitation with anti-GFP affinity beads and then analyzed by western blot with anti-GFP and anti-Flag antibodies.

Supplementary Fig. S4

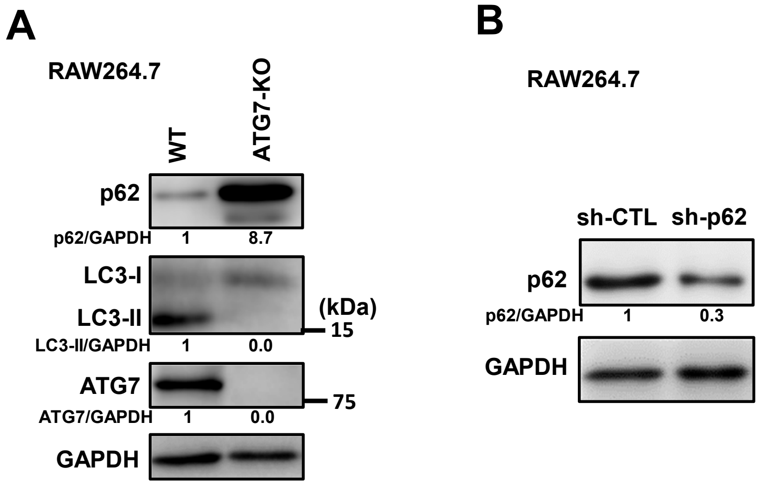

Supplementary Fig. S4 LY287445 induced the degradation of immunoproteasome subunits through autophagy. (A) Confirmation of ATG7 gene knock out in macrophages. ATG7 gene was deleted through CRISPR-CAS9 assay in RAW264.7 cells. Clones of gene deletion cells were cultured and detected for protein levels of p62, LC3, ATG7 and intrinsic control GAPDH. The blots were quantified with densitometric values and statistical significance was analyzed. **(B)** Confirmation of the decrease of p62 protein levels in p62 knock down RAW264.7 cells. The blot was quantified with densitometric values and statistical significance was analyzed.

Fig. 5

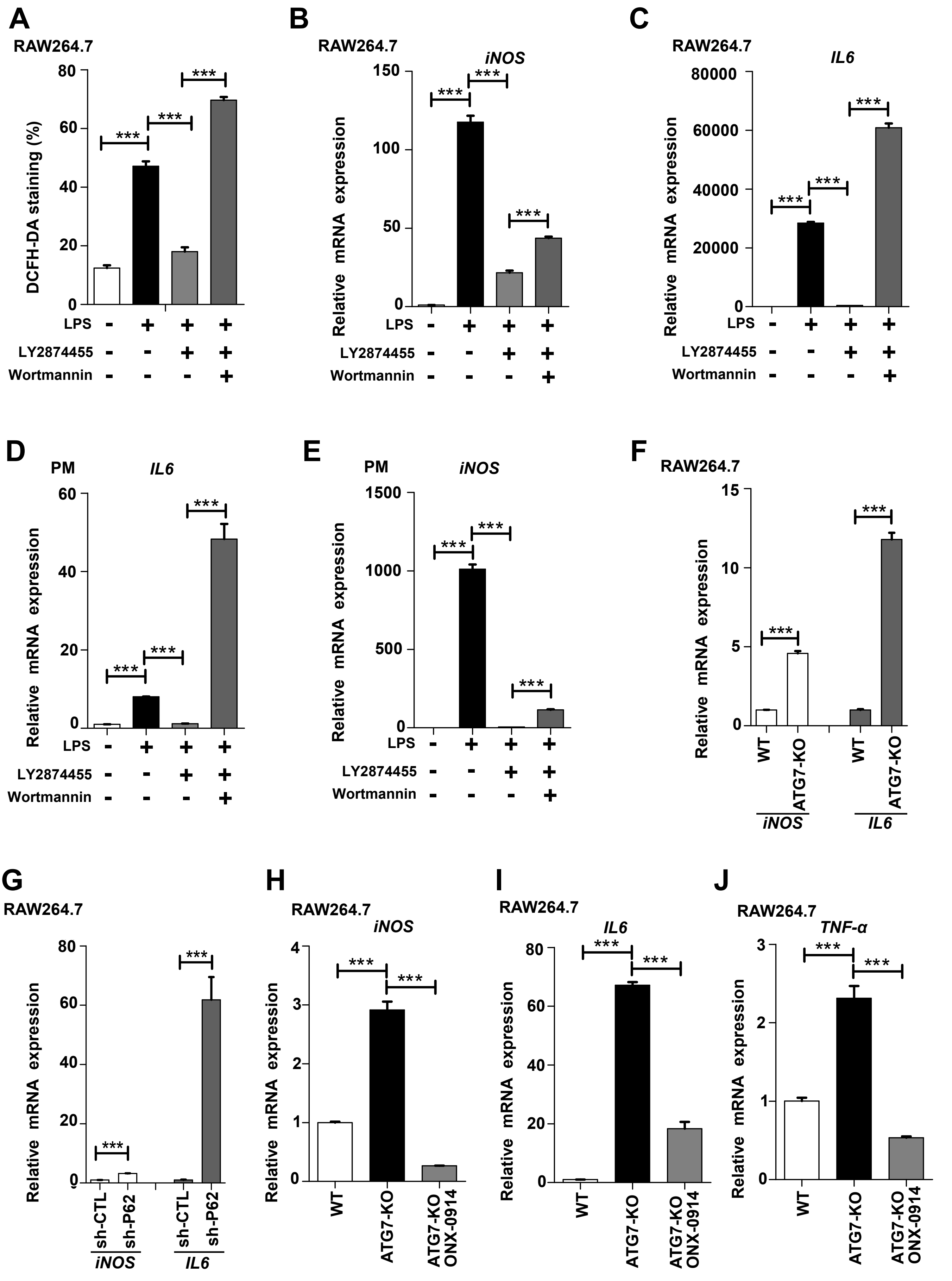

A
RAW264.7
DCFH-DA staining (%)
LPS
LY2874455
Wortmannin
B
RAW264.7
iNOS
Relative mRNA expression
C
RAW264.7
IL6
Relative mRNA expression
D
PM
IL6
Relative mRNA expression
E
PM
iNOS
Relative mRNA expression
F
RAW264.7
Relative mRNA expression
WT
ATG7-KO
iNOS
IL6
G
RAW264.7
Relative mRNA expression
sh-CTL
sh-P62
iNOS
IL6
H
RAW264.7
iNOS
Relative mRNA expression
WT
ATG7-KO
ATG7-KO ONX-0914
I
RAW264.7
IL6
Relative mRNA expression
J
RAW264.7
TNF-α
Relative mRNA expression

Fig. 5 LY2874455 supressed inflammation though autophagy pathway. (A) Autophagy inhibitor reversed the effect of LY2874455 on ROS-reduction in LPS-stimulated macrophages. RAW264.7 cells were stained with ROS-probe DCFH-DA after treatment with LPS (20ng/ml), LY2874455 (2μM) and wortmannin (100nM) as indicated for 24h. Quantitative measurement of cellular ROS was performed by flow cytometry. **(B-C)** Autophagy inhibitor reversed the effect of LY2874455 in LPS-stimulated macrophages. The expression of inflammatory cytokine genes (iNOS and IL-6) was checked by qRT-PCR in RAW264.7 cells as treated as (A). **(D-E)** The expression of inflammatory cytokine IL-6 and iNOS was checked by qRT-PCR in peritoneal macrophages (PM) as treated as in (A). **(F)** Autophagy deficiency reversed the suppression effect of LY2874455 in inflammatory macrophages. Wild type (WT) or ATG7 knock out (ATG7-KO) RAW264.7 cells were treated with LPS (20ng/ml) and LY2874455 (2μM) for 24h. The expression of iNOS and IL-6 was checked by qRT-PCR and expressed as fold changes compared to values in WT cells. **(G)** p62 knock down reversed the suppression effect of LY2874455 in inflammatory macrophages. Control (sh-CTL, control shRNA) or p62 knockdown (sh-p62, p62-targeting shRNA) RAW264.7 cells were treated with LPS (20ng/ml) and LY2874455 (2μM) for 24h. The expression of iNOS and IL-6 was checked by qRT-PCR and expressed as fold changes compared to values in control cells. **(H-J)** The enhanced inflammation in autophagy deficient macrophages was suppressed by immunoproteasome inhibitor ONX-0914. Wild type (WT) or ATG7 knockout (ATG7-KO) RAW264.7 cells treated with LPS (20ng/ml) and ONX-0914 (1μM). The expression of iNOS and inflammatory cytokine genes (IL-6 and TNF-α) was checked by qRT-PCR.

Supplementary Fig. S5

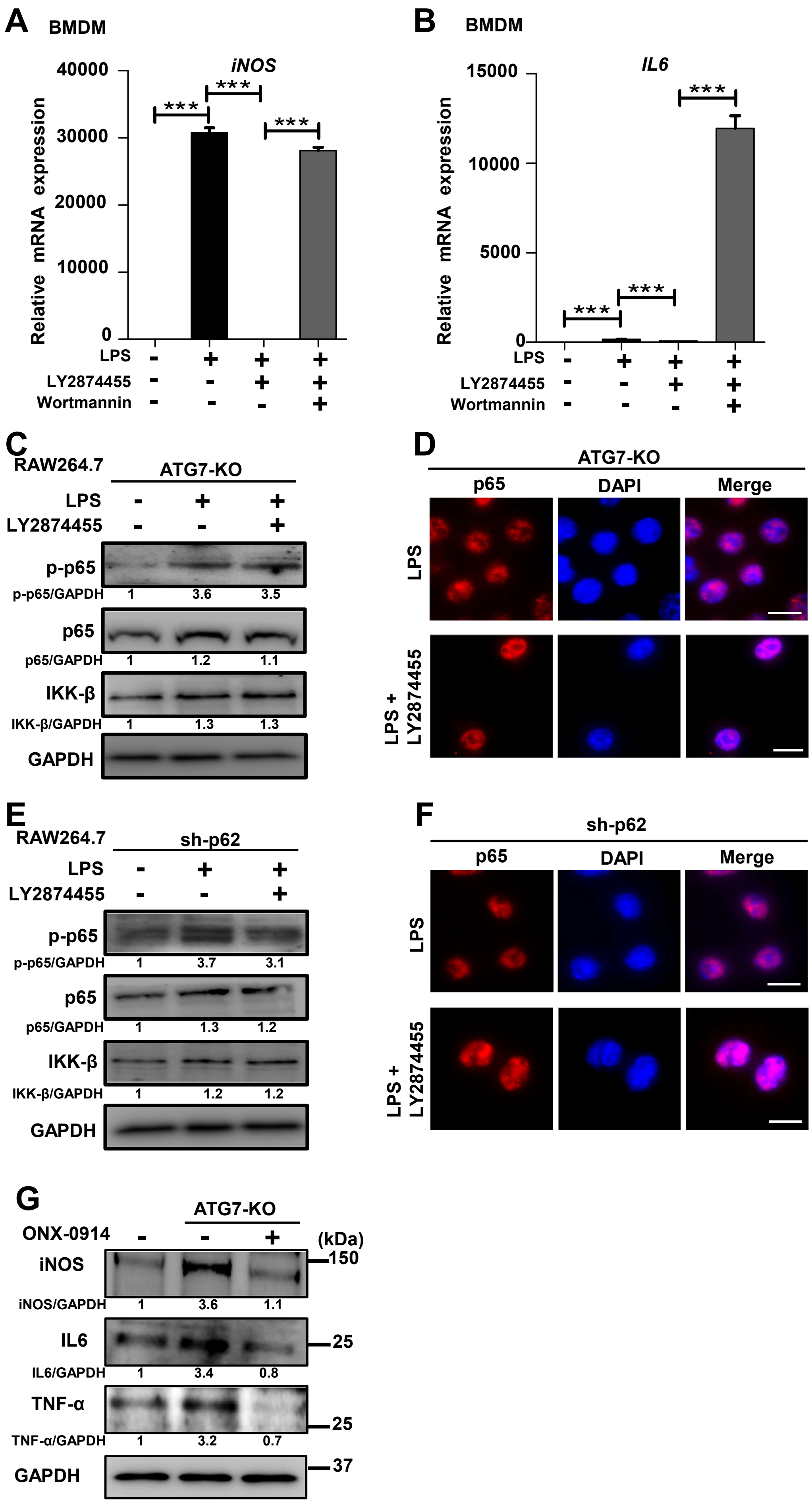

Supplementary Fig. S5 LY287445 suppressed inflammation through autophagy. (A-B) Autophagy inhibitor Wortmannin reversed the effect of LY2874455 on inflammation-suppression in LPS-stimulated macrophages. Bone marrow derived macrophages (BMDM) were treated with LPS (20ng/ml), LY2874455 (2μM) and wortmannin (100nM) as indicated for 24h. The expression of iNOS and inflammatory cytokine genes IL-6 was checked by qRT-PCR. **(C-F)** Deficiency of autophagic ATG7 or receptor p62 caused blockage of LY2874455 function in suppression of NF-κB factor p65 (phosphorylation and nucleus translocation). **(C, E)** Total proteins were extracted from ATG7 knockout or p62 knockdown RAW264.7 cells and used for western blot analysis of protein levels of p65, phosphorylated p65 (p-p65) and IKK-β. The blots were quantified with densitometric values and statistical significance was analyzed. **(D, F)** ATG7 knockout or p62 knockdown RAW264.7 cells were stimulated with LPS (20ng/ml) for 2 h with or without LY2874455 (2μM). Representative images of double immunostaining for p65 and nucleus (DAPI) were shown. Scale bars: 10μm. **(G)** The enhanced inflammation in autophagy deficient macrophages was suppressed by immunoproteasome inhibitor ONX-0914. Wild type (WT) or ATG7 knockout (ATG7-KO) RAW264.7 cells treated with LPS (20ng/ml) and ONX-0914 (1μM). The protein levels of iNOS and inflammatory cytokine genes (IL-6 and TNF-α) were checked. The blots were quantified with densitometric values and statistical significance was analyzed.

Fig. 6

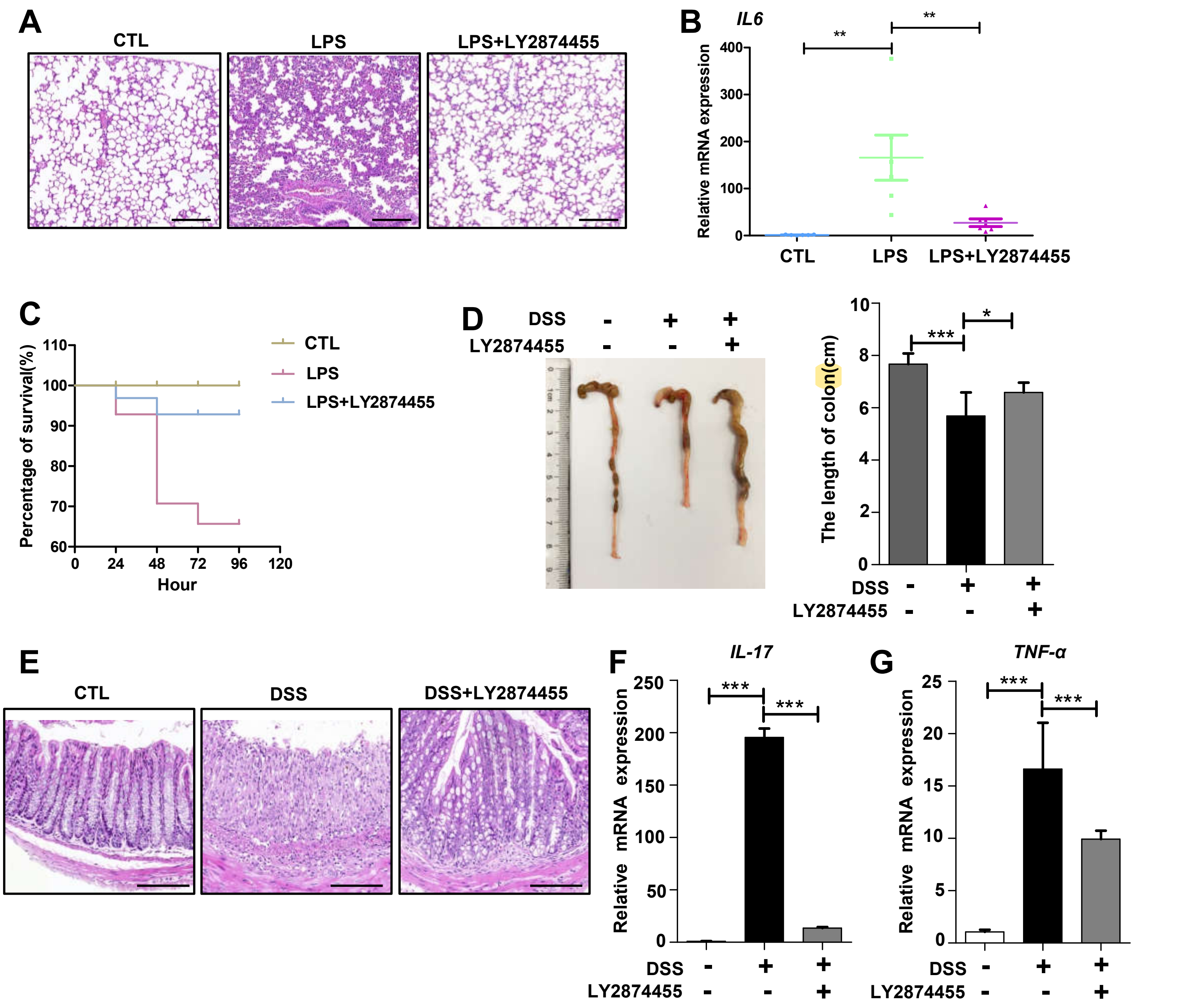

Fig. 6 LY2874455 suppressed lung and intestine inflammation in mouse models. (A) LY2874455 reduced inflammatory injury in lung tissues from acute lung injury mouse models induced by LPS. Two-month-old male C57BL/6J mice were subject to intragastric administration with LY2874455 (1mg/kg) and/or intraperitoneal injection with LPS (10mg/kg) for 6h (n=6). Afterwards, upper lobe of right lungs was collected for HE staining and representative images were displayed. Scale bar: 100μm. **(B)** LY2874455 suppressed the expression of inflammatory cytokine IL-6 in acute lung injury mouse models induced by LPS. Two-month-old male C57BL/6J mice were treated as in (A), and relative mRNA levels of IL-6 in lung tissues (lower lobe of right lungs) were analyzed by qRT-PCR (n=6). **(C)** Two-month-old male C57BL/6J mice were subject to intragastric administration with LY2874455 (1mg/kg) and/or intraperitoneal injection with LPS (35mg/kg) (n=8). The survival test results were presented as Kaplan-Meier survival curves. **(D)** LY2874455 reduced inflammatory injury in intestine tissues from dextran sulfate sodium salt (DSS)-induced inflammatory bowel mouse models. Two-month-old male C57BL/6J mice were subject to intragastric administration with 3% DSS for 7 days and with LY2874455 (1mg/kg) from the third day (n=8). The length of the colons was showed by representative pictures and quantification data. **(E)** LY2874455 reduced intestine injury caused by DSS-induced colitis. Colonic tissues from indicated mice were subject to HE staining. Representative images were shown (n=8). Scale bar: 100μm. **(F-G)** LY2874455 reduced the levels of inflammatory cytokine genes induced in DSS-induced mouse models. The expression of inflammatory cytokine genes IL-17 and TNF-α was checked by qRT-PCR in colons from indicated mice (n=8).

Supplementary Fig. S6

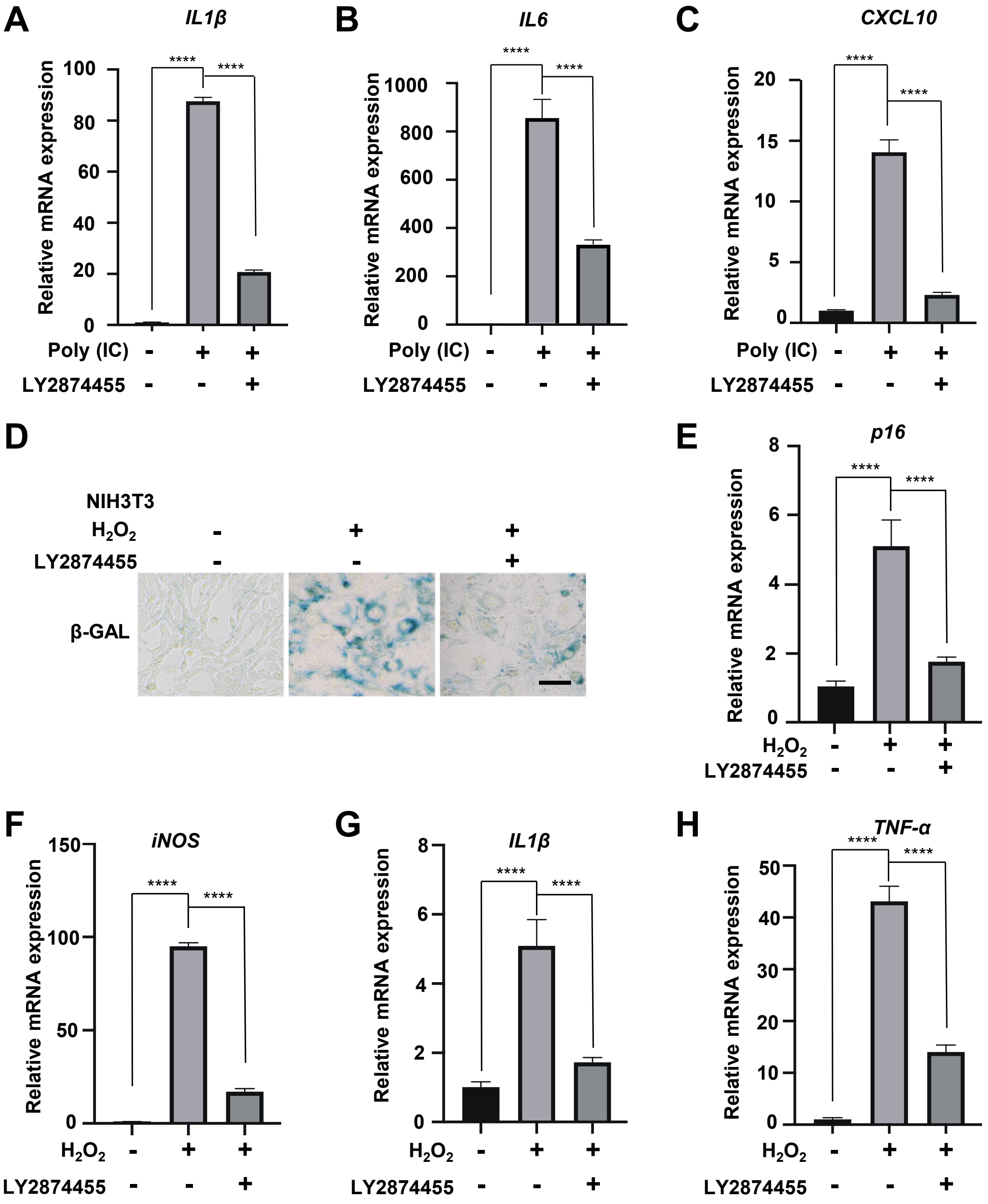

Supplementary Fig. S6 LY287445 suppressed viral infection- and aging-induced inflammation. (A-C) LY2874455 inhibited viral infection-induced inflammation. Polyinosinic-polycytidylic acid (poly(I:C)) (50µg/ml) and LY2874455 (2µM) were applied to BMDM for 24h and expression levels of inflammatory cytokines were analyzed qRT-PCR. **(D-H)** LY2874455 inhibited H_2O_2 induced cell aging. NIH3T3 cells were treated with H_2O_2 (400µM) for induction of cell aging followed by LY2874455 (2µM) treatment. Representative images of β-GAL staining of the cells were shown (D) and the expression levels of p16 (E) and inflammatory cytokines (F-H) were analyzed by qRT-PCR.

Supplementary File

Click here to access/download
Supporting Information
Supplementary tables 1 and 2.docx
