# Supplementary material for: Pharmacological induction of autophagy reduces inflammation in macrophages by degrading immunoproteasome subunits
Source: PLoS Biol. 2024 Mar 6;22(3):e3002537. doi: 10.1371/journal.pbio.3002537 (PMC10917451; doi:10.1371/journal.pbio.3002537)
Supplement: Attachment — Submitted filename: Response to Reviewers-R.pdf [file pbio.3002537.s012.pdf]

Point by point response to the reviewers

All the authors sincerely thank the editor and the reviewers for their time and effort in handling our manuscript (PBIOLOGY-D-23-00322R1). We thank the appreciation of our work from all the three reviewers. The very helpful and detailed suggestions put forth by the reviewers have greatly improved the quality of this work. We are highly touched by the additional careful correction on the text by the reviewer #3.

Following their suggestions, we have substantially revised the manuscript and addressed all issues raised by the reviewers, as shown below.

Reviewer #1

General comments:

"The manuscript by Zhou et al. presents a detailed analysis and interesting findings on the FGFR inhibitor activated autophagy inhibiting inflammation through degradation of immunoproteasomes in macrophages. All the data provided are based on different experimental approaches using macrophage cells and animal models. The experiments are well designed and are clearly presented in this manuscript. The authors first screened and found FGFR inhibitor inhibits inflammation and promotes specific degradation of immunoproteasome in macrophages, then the authors found this inhibitor efficiently activates autophagy, which mediates the effects of this inhibitor in inflammation suppression and immunoproteasome degradation, furthermore, the authors uncovered ubiquitination as the trigger and p62 as the receptor for this selective autophagy of immunoproteasome. With two mouse models, the authors found this inhibitor can function for inflammation suppression in vivo. All in all, the presented data are well documented and very interesting, linking the selective autophagy of immunoproteasome with inflammation suppression in macrophages. I believe that this study could pave the way for new investigations into the connection between autophagy and innate inflammatory signaling and could, therefore, be considered to be published in PLOS Biology after minor revision. Specific points that help improve this manuscript are described below."

Response: We thank the reviewer for the time and all the positive comments such as "All in all, the presented data are well documented and very interesting, linking the selective autophagy of immunoproteasome with inflammation suppression in macrophages.".

The specific comments are addressed as shown below.

Specific comments:

1) "1, The FGFR inhibitor LY2874455 is interesting, can the authors try to detect more other inhibitors of FGFR, to see whether such effects on inflammation suppression, autophagy of immunoproteasome are still conferred by other inhibitors of FGFR? This is important since this will confirm the effects of autophagy of immunoproteasome in inflammation suppression is not only conferred by one inhibitor."

Response: We thank the reviewer for this suggestion. We tried another inhibitor of FGFR, AZD4547, and results showed that this inhibitor also reduced the protein levels of p62, LMP2 and LMP7 (Fig. S4F) and suppressed the inflammation induced by LPS treatment (Fig. S4G-I). This further confirmed the effects of FGFR-regulated autophagy of immunoproteasome in inflammation suppression.

2) "2, The authors focused on macrophages in this study, and data provided are strong enough to support the conclusions. I am curious that whether this FGFR inhibitor can also exert function of autophagy activation in other types of cells, such as fibroblasts?"

Response: We thank the reviewer for this suggestion. We analyzed the effects of FGFR inhibitor LY2874455 on autophagy in NIH3T3 fibroblasts. Results showed that LY2874455 reduced the protein levels of autophagic substrate p62 and increased the protein levels of LC3II (Fig. S4E), indicating that LY2874455 also activates autophagy in fibroblasts.

3) "3, In Fig.1, the authors found FGFR inhibitor, also several other chemicals were found to suppress inflammation such as #8, 10, 19, with less efficiency (Fig.S1A). Can the authors analyze these chemicals to detect their effects on autophagy activation?"

Response: We thank the reviewer for this suggestion. We analyzed the chemicals #8 (Domiphen), #10 (Cetypyridinium) and #19 (Fenticonazole) for their effects on autophagy. Results showed that chemicals #8 (Domiphen) and #19 (Fenticonazole) reduced the protein levels of p62 while chemical #10 (Cetypyridinium) increased the protein levels of p62 (Fig. S4J). This suggested that different chemicals may suppress inflammation through different mechanisms.

4) "4, In Fig.2, the authors analyzed subunits of constituent proteasome, PSMB1, 2, 5, can the authors analyze other subunits? As these three subunits are counterparts of LAMP2, 7, 10, by analyzing other subunits, it will be more convinced that it is the whole constituent proteasome being degraded at the presence of FGFR inhibitor LY2874455."

Response: We thank the reviewer for this suggestion. We analyzed another subunit of constituent proteasome, results showed that LY2874455 did not affect its protein levels (Fig. S3G). This together with previous results (Fig. 3D-G) indicates it is the immunoproteasome but not the constituent proteasome that is degraded in macrophages the presence of FGFR inhibitor LY2874455.

5) "5, In Fig.3F, the p62 blots showed two bands at high levels, is that simply because of the high amount of p62? It is suggested to be repeated this."

Response: As suggested, we repeated this experiment. Results showed that LY2874455 treatment reduced the protein levels of p62 and increased the protein levels of LC3II, which effects were reversed by autophagy inhibitor Bafilomycin A1 (Fig. 4F).

6) "6, The imaging pictures should be organized more carefully, for example, in Fig. 1K and Fig. 2D, the panel pictures are not aligned. Similarly, the WB blots should also be organized more carefully, for example, in Fig. S5G, the upper two panels should be reorganized. I suggest the authors go through all the data to check the format."

Response: We thank the reviewer for this suggestion. We went through all the data and organized the imaging pictures and WB blots.

Reviewer #2: Christian Münz – note that this reviewer has signed his review.

General comments:

"The authors identified (R)-(E)-2-(4-(2-(5-(1-(3,5-dichloropyridin-4-yl) ethoxy)-1H180 indazol-3yl) vinyl)-1H-pyrazol-1-yl) ethanol (LY2874455) as a compound that limits nitric oxide (NO), reactive oxygen species (ROS), TNF and IL-6 production by the mouse macrophage cell line RAW264.7 upon LPS stimulation. A similar phenotype has been observed in mouse peritoneal and bone marrow derived macrophages and the human monocyte cell line THP-1. This compound has previously been reported to be a pan-fibroblast growth factor receptor (FGFR) inhibitor. They demonstrate that LY2874455 stabilizes IKK-beta preventing NF-kappaB translocation to the nucleus and pro-inflammatory mediator production after LPS stimulation. The proteomic analysis of LY2874455 treated RAW264.7 cells revealed depletion of immunoproteasomes and their inhibition with ONX-0914 phenocopies the hypoinflammation upon FGFR inhibition. Furthermore, the authors determine with wortmannin and chloroquine inhibition that immunoproteasomes are turned over by autophagy, and that LY2874455 increases autophagic flux of the mCherry-EGFP-LC3 reporter construct and the p62 autophagy receptor. ATG7 deficiency and RNA silencing of p62 increased immunoproteasome levels. Finally, the authors demonstrate that LY287445 reduces LPS induced lung and DSS induced bowel inflammation, and in supplemental data on polyI:C and age induced inflammmation. Therefore, the authors suggest that FGFR inhibition induces autophagy that degrades immunoproteasomes which in turn are required for NF-kappaB dependent inflammation.

The study is interesting and provides many mechanistic insights into the role of autophagy and immunoproteasomes in inflammation. However, it suffers from not discussing prior literature on autophagic mechanisms that limit inflammation and proteasomal degradation by autophagy, both of which diminish the novelty of the reported findings. Furthermore, the specificity of the FGFR and immunoproteasome inhibitors are not controlled for by respective knock-outs and knock-downs and only surrogate pathogen associated molecular pattern (PAMP) molecules are used to mimic bacterial and viral infections."

Response: We thank the reviewer for the time and the positive comments such as "The study is interesting and provides many mechanistic insights into the role of autophagy and immunoproteasomes in inflammation.".

The comments from the reviewer are specific and very helpful, and they are addressed as shown below.

Major comments:

1) "1. The authors introduce inflammatory tissue damage during COVID-19 and sepsis but then use primarily LPS challenge. It would be interesting if immunoproteasome degradation would influence inflammation upon exposure to bacteria or viruses. Along these lines the authors should also not call polyI:C induced inflammation viral infection and H2O2 exposure age-related inflammation, in the text referring to supplemental figure 6."

Response: We thank the reviewer for these suggestions. We analyzed the inflammation upon exposure to bacteria. E. coli exposure induced inflammation shown by upregulated expression of IL6, TNF-α and iNOS, which were efficiently inhibited by LY2874455 treatment (Fig. S2D-F).

As suggested, we no longer call polyI:C induced inflammation viral infection and H_2O_2 exposure age-related inflammation, in the text referring to supplemental figure 6 (now Fig. S2 A-K).

2) "2. The presented data with the two pharmacological inhibitors for FGFR and immunoproteasomes should be confirmed at least for RAW264.7 produced inflammatory mediators by RNA silencing or knock-out for FGFR or immunoproteasome subunits. Even so LY2874455 and ONX-0914 might be very specific for their targets only the absence of their effects upon FGFR or immunoproteasome deficiency, respectively, would clearly document this."

Response: We thank the reviewer for this suggestion. We knocked down the expression of immunoproteasome subunit LMP7 (Fig. S6L) and analyzed the effects of immunoproteasome inhibitor ONX-0914. Results showed that this inhibitor cannot suppress inflammation in LMP7 knockdown cells (Fig. S6M), indicating that this inhibitor is specific to immunoproteasome. For FGFR inhibitor, we encountered difficulties as the inhibitor LY2874455 is a pan-FGFR

inhibitor and FGFR1-4 were all expressed in macrophages (with FGFR2 and FGFR4 higher expression). Knockdown of both FGFR2 and FGFR4 simultaneously is difficult for us at the moment.

3) "3. Inflammasome regulation by autophagy has been studied by many investigators as mechanism of hyperinflammation upon autophagy deficiency in myeloid cells. Therefore, the authors should assess inflammasome output, such as IL-1beta and IL-18, upon LY2874455 treatment and its likely independence of ONX-0914 mediated immunoproteasome inhibition. This would also confirm specificity of the used inhibitors."

Response: We thank the reviewer for this suggestion. We analyzed the inflammasome output, IL-1beta and IL-18, upon LY2874455 treatment. Results showed that LY2874455 treatment reduced the protein levels of IL-1beta and IL-18 (Fig. S2L). This makes sense as LY2874455 activates autophagy and autophagy restricts inflammasome-mediated inflammation.

4) "4. Some of the immune fluorescence micrographs should be quantified and stated how many experiments were performed, such as the nuclear p65 translocation in Figures 1K, S2A, S5D and S5F, the autophagy reporter in Figure 3D, and co-localization of immunoproteasome subunits with LC3 in Figure 4D."

Response: We thank the reviewer for this suggestion. We quantified the immune fluorescence micrographs Fig. 1K (quantified in S1AF), S2A (now Fig. S3A, quantified in S3M), S5D (now S6F, quantified in S6F), S5F (now S6H, quantified in S6H), 3D (now Fig. 4D, quantified in S4N) and 4D (now Fig. 5D, quantified in S5F). All experiments were independently repeated at least three times with consistent conclusions and representative results were shown. Statistical analyzes were performed in Prism software. Values are expressed as mean ± SD of at least three independent experiments, unless otherwise noted. Images were analyzed with ImageJ. The total intensity in each color channel was counted as the denominators before analyzed for quantification of colocalization ratios. This counting was repeated three times with samples from replicated experiments.

5) “5. The authors identify FGFR inhibition as stimulus to increase autophagic flux. As they discuss, autophagy stimulation would be beneficial in many disease settings and, therefore, well tolerated autophagy inducers are of great interest. However, previous literature has suggested that FGF signaling actually induces autophagy in some cell types, such as chondrocytes (Cinque et al., Nature 2015). Therefore, any insights that the authors can provide on why FGFR inhibition causes autophagy up-regulation in their experimental system, while FGFR signaling stimulates autophagy in other cell types would clarify a broad application of FGFR inhibitors to stimulate autophagy.”

Response: We thank the reviewer for this suggestion. We speculate that in certain types of cells, autophagy is activated under stress conditions, means the normal growth condition (regulated by FGFs and FGFRs) suppresses autophagy. When such cells are subject to inhibition of growth signaling such as FGFR inhibitor treatment, autophagy is then activated. We observed autophagy activation by FGFR inhibitor LY2874455 in macrophages, in fibroblasts (Fig. S4E) and in HEK293T cells and HeLa cells (data not shown).

Autophagy not only functions as a degradation pathway, it also functions to facilitate extracellular secretion. In the study on chondrocytes (Cinque et al., Nature 2015), the authors found that autophagy is induced in growth-plate chondrocytes and regulates the secretion of type II collagen (Col2). We speculate that in chondrocytes at growth conditions, autophagy is activated and necessary for cell secretion of certain factor.

Hence, autophagy functions differently in different types of cells. It is interesting that FGF signaling (FGF18 and FGFR4) activates autophagy in chondrocytes (Cinque et al., Nature 2015) while in our case FGF signaling inhibition (FGFR inhibitor LY2874455 treatment) activates autophagy. It is possible that the main functional downstream kinases of FGF signaling may be different in different types of cells, for example JNK in chondrocytes (Cinque et al., Nature 2015) while AKT and mTOR in macrophages (Fig. 4J and S4D).

This is similar to the fact that autophagy functions differently (promoting or inhibiting) in different types of cancer cells. To realize the different functions, autophagy is activated or inhibited by specific downstream signaling pathways even when the very upstream signaling is the same.

Minor comments:

1) “1. Some typos: line 127, achieved instead of chieved; line 463, Beclin1 instead of Belin1.”

Response: We thank the reviewer for the careful examination. We corrected these mistakes and also went through the whole manuscript for checking.

3) “3. Similarly no previous literature on autophagy of proteasomes is cited. This should be done, e.g. Sengupta et al., EMBO J 2019 and Dengjel et al., Mol Cell Proteomics 2012.”

Response: We thank the reviewer for this suggestion. These previous literatures have been cited.

Reviewer #3: **Anna Katharina Simon - note that this reviewer has signed her review**

General comments:

“The authors demonstrate that the FGFR inhibitor LY2874455 can limit production of pro-inflammatory cytokines by LPS-stimulated macrophages through selective degradation of immunoproteasome subunits via autophagy. This finding is novel and provides an additional explanation for exaggerated inflammatory processes observed upon autophagy-deficiency. The experiments in this study are well designed and executed. Especially, the use of primary murine macrophages and bone-marrow derived macrophages in addition to the RAW264.7 cell line and confirmation of the efficacy of the inhibitor in different in vivo models are major assets of the study. Besides grammar and spelling of the manuscript that have to be improved (some of them have been indicated in the pdf document) and inaccuracies in the method section, we would like to see the following major and minor points addressed in the revised manuscript.”

Response: We thank the reviewer for the time and the positive comments such as “This finding is novel and provides an additional explanation for exaggerated inflammatory processes observed upon autophagy-deficiency. The experiments in this study are well designed and executed.”.

We would like to highly thank the reviewer for the detailed examination on the text and figures of the manuscript.

The comments from the reviewer are very helpful and are addressed as shown below.

Major comments:

1) “Point 1

The authors state, that treatment with LY2874455 did not further repress production of inflammatory cytokines in ONX-0914-treated macrophages in Figure 2I and J (Lines 248-250). However, the graph and the figure legend indicate, that cells were either treated with LY2874455 or ONX-0914, thus making this statement invalid. Furthermore, a combination of these treatments would not prove, that LY2874455 acts through inhibition of the immunoproteasome, as ONX-0914 inhibition already abolishes production of pro-inflammatory cytokines, which cannot thus not be further reduced by LY2874455. We ask the authors to a) correct the description of the aforementioned figure and b) comment on their conclusion.”

Response: We corrected the labeling and legend of figure 2I and J (now Fig. 3I, J) to the experiment setting that LY2874455 and ONX-0914 were used in combination.

We agree with the reviewer that the statement on Y2874455 acting through inhibition of the immunoproteasome is invalid. We speculated that since LY2874455 cannot further suppress the production of pro-inflammatory cytokines at the presence of immunoproteasome inhibitor ONX-0914, LY2874455 might function at the upstream of immunoproteasome. We tuned down the previous conclusion.

2) “Point 2

We are missing a statement on normal distribution of the data in the statistical analysis paragraph (starting in line 744). This concerns all data in the manuscript. Which tests were used to probe for normal distribution of the data? In some graphs, normal distribution of the data is questionable and thus statistical analysis could be incorrect.”

Response: We checked the data with Shapiro-Wilk test and found that some data were not normally distributed. Hence, to ensure the accuracy of the statistical analysis, the Dunn’s nonparametric test (for multiple group comparisons) and the Mann–Whitney test (two group comparisons) were used.

3) “Point 3

Throughout the figure legends, it was mentioned that western blot signals were quantified with densitometric values and statistical significance was analyzed. However, while the ratios of densitometric values of protein of interest and housekeeping protein are provided in the graphs, the results of the statistical analysis are missing. It is also not clear how often the western blot experiments were reproduced with independent replicates. Therefore, we ask the authors to add the quantification and statistical analysis for all western blots displayed and indicate how many replicates were analyzed for all western blots displayed throughout the manuscript.”

Response: We thank the reviewer for this suggestion. The statistical analysis of WB blots is added. All experiments were independently repeated at least three times. This information is added in figure legend (for example, in Fig. 1H legend: The WB were independently repeated at least three times and quantified in Fig. S1W-Y).

4) “Point 4

The method used in Figure 3D is not well explained in the results (line 275-279) or method section or in the figure legend. We assume, that the GFP signal is quenched upon autophagosome-lysosome fusion, but the mCherry signal is not affected. However, this is not clearly stated by the authors and the explanation given in the figure legend is quite confusing. Also, statistical analysis of this

figure is missing and the results are questionable as no lysosomal inhibitor was used, which would reveal if there was indeed stalling of the autophagic process or rather increased LC3 expression. Therefore, we consider the conclusion drawn in lines 286-288 (stating that LPS blocked autophagic flux) invalid. A western blot detecting LC3I and II and the use of a lysosomal fusion inhibitor such as Bafilomycin A would be the appropriate control experiment."

Response: We thank the reviewer for these suggestions. We added information in the manuscript text to explain the results of Fig. 3D (now Fig. 4D). The reviewer is right that the GFP signal is quenched upon autophagosome-lysosome fusion, but the mCherry signal is not affected, therefore the red signal indicates autophagy activation. As suggested, a quantification of this results (Fig. 4D) was shown (Fig. S4N). A western blot detecting LC3I and II and the use of a lysosomal fusion inhibitor Bafilomycin A was performed. Results showed that LY2874455 reduced the protein levels of autophagy receptor/substrate p62 and increased the protein levels of autophagosome marker LC3II, which was reversed by Bafilomycin A (Fig. 4F).

5) "Point 5

The methods proving that autophagy is indeed activated upon treatment with LY2874455 as displayed in figure 3 are not completely sufficient. A) Some of the arrows in figure 3E are not pointing at autophagosomes, but rather lysosomes. Therefore, the quantification presented in Figure S3D is questionable. B) Quantification of LC3I and II in addition to p62 would be needed to actually prove induction of involvement of autophagy.

Finally, wortmannin is not considered the best agent for autophagy inhibition, as it targets other processes. The use of chloroquine, as presented in figure 3B, is preferred and we therefore wonder, why Chloroquine was not used in the experiments presented in Figure 5. Also, use of a more specific autophagy inhibitor such as ULK-1 inhibitor would be a better option."

Response: We thank the reviewer for these suggestions. A) The autophagosomes in Fig. 3E (now Fig. 4E) were reanalyzed and pointed, also the quantification was reperformed (Fig. S4O). B). Quantification of LC3I and II in addition to p62 was done and results indicated that LY2874455 reduced the

protein levels of autophagy receptor/substrate p62 and increased the protein levels of autophagosome marker LC3II, which was reversed by Bafilomycin A (Fig. 4F, quantified in Fig. S4P).

The reviewer is right on wortmannin and chloroquine. The reason we used wortmannin was that we wanted to block autophagy at the upstream step. As suggested, we used ULK-1 inhibitor to confirm the autophagy activation by LY2874455. Results showed that both inhibitors of autophagy, ULK1-IN-2 (moderately) and chloroquine (CQ) (strongly) reversed the suppression of inflammation by LY2874455 (Fig. S6J, K).

6) "Point 6

We have serious doubts about the validity of only using GAPDH (an enzyme in the glycolysis pathway) as a housekeeping gene and/or protein for all qRT-PCR and western blot experiments. It has been demonstrated by various studies, that GAPDH expression is triggered by deletion or inhibition of autophagy, LPS stimulation of macrophages and other treatment of cells performed in this manuscript. Thus, we ask the authors if they have used additional housekeeping proteins / transcripts as a control and if the results obtained using other control proteins / transcripts were similar.

The results could also benefit from additional analysis of cytokine concentrations in the supernatant using ELISA, as this is considered more reliable than mRNA expression, especially for cytokines."

Response: We thank the reviewer for these suggestions. We repeated the qRT-PCR and western blot experiments using another housekeeping gene/protein β-actin (qRT-PCR results in Fig. S1E-G and western blot results in Fig. S1H). Results confirmed that LY2874455 suppressed the LPS-induced expression of inflammatory factors (Fig. S1E-G) and reduced the protein levels of IL6, TNF-α and iNOS (Fig. S1H).

As suggested, we performed additional analysis of cytokine concentrations in the supernatant using ELISA. Results showed that LY2874455 efficiently reduced the amounts of cytokines induced by LPS (Fig. S1U, V).

7) "Point 7

In our opinion, the DSS-induced colitis model is not properly evaluated. A) We wondered whether the authors did not measure mRNA expression or protein expression of IL-6 and IL-1b. B) To properly evaluate this model, other parameters should be provided like a histology scoring of the intestine, as well as changes in body weight of the mice."

Response: We thank the reviewer for these suggestions. A) As suggested, we analyzed the mRNA expression levels of IL-6 and IL-1β in the DSS-induced colitis model. Results showed that LY2874455 suppressed the expression levels of IL-6 (Fig. 2L) and IL-1β (Fig. 2I). Also, the suppression on the expression of IL-17 and TNF-α by LY2874455 was observed (Fig. 6J, K). B) As suggested, we analyzed histology scoring of the intestine, as well as changes in body weight of the mice. Results showed that LY2874455 treatment reduced the intestinal damage (Fig. 2H) and loss of body weight (Fig. 2E) caused by DSS.

8) "Point 8

We would like to suggest to restructure the manuscript in a way, that the in vivo data is mentioned before mechanism."

Response: We thank the reviewer for this suggestion. Indeed, putting the *in vivo* data before mechanism is better. We restructured the manuscript accordingly to first show the suppression of inflammation by LY2874455 in cell models and in mouse models, then show the mechanism.

9) "Point 9

We feel, that the title is overinterpreting the results, as there are not many convincing experiments using autophagy KO or inhibition alone, and the autophagy inducing compound used may affect more processes than autophagy. As an alternative title we suggest: Pharmacological induction of autophagy reduces inflammation in macrophages by degrading immunoproteasome subunits."

Response: We thank the reviewer for this suggestion. The suggested title is nice and better. We used it as the title.

Minor comments:

1) "Point 1

The writing of the manuscript has to be improved. There are a lot of spelling mistakes and grammar errors, which complicate the reading of the manuscript. Some of them are addressed in the commented version of the PDF."

Response: We highly thank the reviewer for the detailed examination on the text and figures of the manuscript. We corrected all these spelling mistakes and grammar errors. Further, we sent the manuscript for professional language editing at AJE company (https://www.aje.cn/, Order number: 9NMK8RX7).

2) "Point 2

Throughout the manuscript, the authors refer to the observation made using macrophage stimulation in vitro to changes in "inflammation". However, this cannot be stated as only expression of pro-inflammatory factors (mostly on mRNA level) was investigated and inflammation rather refers to the biological process in the body. We ask the authors to rephrase the respective sentences."

Response: As suggested, we rephrased the respective sentences from "inflammation" to "expression of pro-inflammatory factors ".

3) "Point 3

The Introduction contains a lot of information which is not needed for understanding the manuscript. Especially the part from line 65 to 106, where regulation of RTKs is explained in detail could be omitted, and instead papers could be cited more in detail that actually deal with immunoproteasome and inflammation."

Response: We thank the reviewer for this suggestion. The description on regulation of RTKs was deleted and more immunoproteasome and inflammation related information was added.

4) "Point 4

Several references are missing throughout the manuscript. Those are indicated in the commented version of the PDF."

Response: We checked the manuscript to make sure the missing references are added.

5) “Point 5

The authors state, that 5618 components were tested for potential inhibition of NO production by LPS-stimulated RAW264.7 macrophages. However, only the results of 27 are shown and there is no comment on the results of the other compounds. We are missing a statement here, whether NO production was unaffected by all other components. Additionally, it would be nice to provide the results for all components tested as a supplementary file to the scientific community.”

Response: Among all the chemicals used for screening, there are many showed potential inhibition of NO production by LPS-stimulated RAW264.7 macrophages. The reason that we did not show the results for all chemical tested was that there are several students in the lab are working on analyzing the effects of inflammation suppression and behind mechanisms. We hope this could get the understanding from the reviewer.

6) “Point 6

Line 270: The results of figures S3A and B are not mentioned in the text.”

Response: We added description of Figures S3A and B (now Fig. S4A, B) in the text.

7) “Point 7

The last three lanes of Figure 3F are not explained in the text (see lines 284-286).”

Response: The results of Figure 3F (now Fig. 4F) were repeated as requested and were explained in detail in the text.

8) “Point 8

The authors state in line 297, that mTOR inhibits autophagy by initiating ULK1 kinase complex. However, this is not true. mTOR targets Ulk1 but inhibits the activation of the complex through inhibitory phosphorylation, as it is correctly stated in the figure legend of Fig. S3. This should be corrected.”

Response: As suggested, we corrected the description to “mTOR targets Ulk1 but inhibits the activation of the complex through inhibitory phosphorylation”.

9) “Point 9

In Figure 4, most of the blots do not contain annotation for molecular weights. Please provide those.”

Response: Molecular weights are labeled to all the blots in Figure 4 (now Fig.5).

10) “Point 10

To us, it is not clear how figure 4G proves ubiquitination of LMP2 and LMP7, as there are no bands around 50 kDa in the right upper panel of Figure 4G. We ask the authors to comment on that.”

Response: The proteins of LMP2 and LMP7 were immunoprecipitated and ubiquitin antibody was used to detected the ubiquitinated forms of LMP2 and LMP7. The higher bands on this blot indicated that LMP2 and LMP7 are extensively ubiquitinated so that they appear as high molecular weight bands above 50 kDa.

11) “Point 11

Please split the axis of Figure S5B to visualize the difference between LPS and LPS+LY2874455.”

Response: The axis of Figure S5B (now Fig. S6B) were reorganized as suggested.

12) “Point 12

The poly (IC) model used in Figure S6A-C is not mentioned or explained in the methods section. Additionally, this is not an actual model of viral infection but rather a model mimicking viral infection. Please add description of the model and amend the statement in line 398.”

Response: As suggested, the poly (IC) model used in Figure S6A-C (now Fig. S2A-C) is mentioned in the methods section. The statement is corrected.

13) “Point 13

The statement in lines 400-402 was not investigated and thus not confirmed in the mouse models mentioned in this paragraph. Therefore, this statement should be deleted or amended.”

Response: We thank the reviewer for this suggestion. This statement is deleted.

14) “Point 14

The statement on immunoproteasome-induced inflammatory processes provided in lines 428 to 430 seems oversimplified and is missing a reference. Please add more specific information and cite studies proving this connection.”

Response: We agree with reviewer. This statement is not appropriate and just a speculation. We deleted this statement.

15) “Point 15

Materials and Reagents paragraph (starting line 523): If it is allowed by the journal guidelines, a table would be much more comprehensive instead of the text written in this paragraph.”

Response: We thank the reviewer for this suggestion. We checked several published papers in the journal, usually the information in Antibodies and Reagents is listed in this way.

16) “Point 16

It is unclear if the statement given on injection of thioglycolate in line 552 means, that the injections took place 3 days prior to the sacrifice, or 3 days in a row followed by the sacrifice of the mice.”

Response: The injections took place 3 days prior to the sacrifice of the mice. The statement is corrected to be: 6-8 weeks aged mice were injected with 3% thioglycolate prior to the sacrifice of the mice and the isolation of BMDM.

17) “Point 17

There are several unclear or missing statements on procedures, composition of media or manufacturers in the methods sections which are marked in the commented version of the PDF.”

Response: We highly thank the reviewer for pointing these unclear or missing statements. We corrected these descriptions accordingly.

18) “Point 18

We wondered whether approval numbers provided by authorities have to be mentioned in the animal experiments section (line 695-698).”

Response: We added the approval number (20220406005) in the animal experiments section.

19) "Point 19
From the description in the methods section (lines 709-715) it is unclear, whether the mice not receiving LY2874455 received a mock injection with e.g. saline as a control. Please add a sentence clarifying this issue."
Response: We added this sentence (The mice not receiving LY2874455 received a mock injection with saline as controls.).

20) "Point 20
A display of wildtype cells in figure S5C-F is missing, which is in our opinion needed to prove that the experiment was successful and p65 does translocate to the nucleus upon LPS treatment in presence of autophagy."
Response: We thank the reviewer for this suggestion. The experiments displayed wildtype cells in figure S5C-F (now Fig. S6D-H) were added. Results showed that in wildtype cells p65 is activated and translocated to the nucleus upon LPS treatment, which is suppressed by LY2874455 (Fig. S6C, E).
