# Supplementary material for: Pharmacological induction of autophagy reduces inflammation in macrophages by degrading immunoproteasome subunits
Source: PLoS Biol. 2024 Mar 6;22(3):e3002537. doi: 10.1371/journal.pbio.3002537 (PMC10917451; doi:10.1371/journal.pbio.3002537)
Supplement: Attachment — Submitted filename: Response to Reviewers.pdf [file pbio.3002537.s013.pdf]

Point by point response to the reviewers

All the authors sincerely thank the editor and the reviewers for their time and efforts in handling our manuscript (PBIOLOGY-D-23-00322R2).

Rev. 1:

"The authors addressed all my questions."

Response: We thank the reviewer for the time and the positive comments.

Rev. 2:

General comments:

"The authors identified (R)-(E)-2-(4-(2-(5-(1-(3,5-dichloropyridin-4-yl) ethoxy)-1H180 indazol-3yl) vinyl)-1H-pyrazol-1-yl) ethanol (LY2874455) as a compound that limits nitric oxide (NO), reactive oxygen species (ROS), TNF and IL-6 production by the mouse macrophage cell line RAW264.7 upon LPS stimulation. A similar phenotype has been observed in mouse peritoneal and bone marrow derived macrophages and the human monocyte cell line THP-1. This compound has previously been reported to be a pan-fibroblast growth factor receptor (FGFR) inhibitor. They demonstrate that LY2874455 stabilizes IKK-beta preventing NF-kappaB translocation to the nucleus and pro-inflammatory mediator production after LPS stimulation. The proteomic analysis of LY2874455 treated RAW264.7 cells revealed depletion of immunoproteasomes and their inhibition with ONX-0914 phenocopies the hypoinflammation upon FGFR inhibition. Furthermore, the authors determine with wortmannin and chloroquine inhibition that immunoproteasomes are turned over by autophagy, and that LY2874455 increases autophagic flux of the mCherry-EGFP-LC3 reporter construct and the p62 autophagy receptor. ATG7 deficiency and RNA silencing of p62 increased immunoproteasome levels. Finally, the authors demonstrate that LY287445 reduces LPS induced lung and DSS induced bowel

inflammation, and in supplemental data on polyI:C and age induced inflammmation. Therefore, the authors suggest that FGFR inhibition induces autophagy that degrades immunoproteasomes which in turn are required for NF-kappaB dependent inflammation.

In their revised manuscript, the authors have addressed most of my major concerns, namely used E. coli exposure in addition to LPS and toned down the interpretation of polyI:C and H2O2. Furthermore, they performed specificity controls for their inhibitors and observe also reduced inflammasome activity in addition to immunoproteasome dependent NF-kappaB activity upon autophagy stimulation. They have now also quantified the immune fluorescence microscopy images.

The authors have also addressed two of my three minor concerns but somehow have not answered to my minor point #2 in the point by point. Therefore, I would like them to acknowledge the previous literature on autophagic suppression of inflammation and the respective mechanisms to clarify that their mechanism is one of many and that their study is not the first that documents hypoinflammation upon autophagy stimulation."

Response: We thank the reviewer for the time and the positive comments on our revised manuscript.

We are very sorry for forgetting to answer the previous minor point #2. Since this point was raised in the followed Minor comment, we addressed it there.

Minor comment:

1) "There is quite some literature on mechanisms by which autophagy restricts inflammation, regulating inflammasomes, STING and MAVS for cytosolic pathogen associated molecular pattern (PAMP) recognition. Therefore, the statement in lines 401 to 402 should be corrected and these previously described mechanisms acknowledged."

Response: We deleted the statement in lines 401 to 402 “Hypotheses on the precise regulation of inflammation by selective autophagy are fascinating; however, the underlying mechanism has been elusive.”.

New description is added (lines 401-406): It has been found that disruption of autophagy leads to diseases with inflammatory components, including infections, autoimmunity and metabolic disorders[149-153]. Autophagy restricts inflammation through various mechanisms by targeting inflammatory mediators including inflammasomes, STING and MAVS for cytosolic pathogen associated molecular pattern (PAMP) recognition[154-162].
